# How to diagnose TB in migrants? A systematic review of reviews and decision tree analytical modelling exercise to evaluate properties for single and combined tuberculosis screening tests

**DOI:** 10.1183/13993003.02000-2024

**Published:** 2025-07-24

**Authors:** Dominik Zenner, Hassan Haghparast-Bidgoli, Tahreem Chaudhry, Ibrahim Abubakar, Frank Cobelens

**Affiliations:** 1 Wolfson Institute of Population Health, Queen Mary University of London, London, UK; 2 Institute for Global Health, University College London, London, UK; 3 Amsterdam University Medical Centers, location Universiteit of Amsterdam, Department of Global Health, Amsterdam Institute for Global Health and Development, Amsterdam, the Netherlands; 4 Queen Mary and Barts Health Tuberculosis Centre, Faculty of Medicine and Dentistry, Queen Mary University of London, London, UK

## Abstract

**Background:**

Optimising tuberculosis disease testing algorithms is fundamental to ensuring the effectiveness and cost-effectiveness of migrant screening programmes, including better understanding of individual and combined screening test properties. The aim of our study was to estimate pooled tuberculosis test properties from the literature and combine them in decision analytical modelling with a focus on whether tests used for the diagnosis of tuberculosis infection might add value to these algorithms.

**Methods:**

We performed a systematic review of reviews of diagnostic tests for active tuberculosis, searching PubMed, Embase, Web of Science and Cochrane library, and pooled test properties extracted from original papers included in reviews. We used these pooled results in a decision tree analysis to estimate test properties for common migrant screening algorithms.

**Results:**

We retrieved 1477 records and included 32 reviews, including data from 437 original studies for 18 tuberculosis tests, providing pooled results for 13. Our modelling showed that algorithms with interferon-γ release assays had the highest diagnostic odds ratios (dORs) (*e.g.* QuantiFERON/chest X-ray (for tuberculosis abnormalities)/Xpert dOR 24 670, 95% CI 11 630–52 328) and high positive predictive values. Best sensitivities were achieved for combinations with parallel cough/chest X‑ray screening followed by Xpert (0.88, 95% CI 0.86–0.90) or Ultra (0.92, 95% CI 0.90–0.94) as well as by T-Spot.TB followed by parallel symptom/chest X-ray screening and Ultra (0.81, 95% CI 0.78–0.83) or Xpert (0.77, 95% CI 0.75–0.80).

**Conclusions:**

The significant test accuracy benefit of adding interferon-γ release assays to an active tuberculosis screening pathway will help inform clinicians and policy-makers on the most effective screening algorithms.

## Shareable abstract

**There is a significant test accuracy benefit of adding IGRAs to an active TB screening pathway, which will help inform clinicians and policy-makers on the most effective screening algorithms** https://bit.ly/3EgtJ4R

## Graphical abstract

**Figure:**
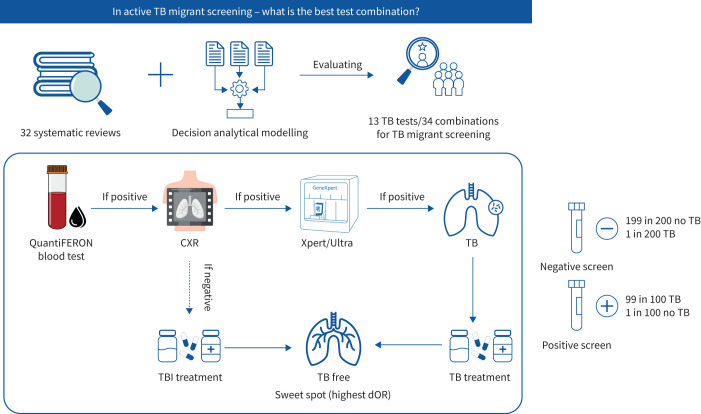
Overview of the study. TB: tuberculosis; CXR: chest X-ray; dOR: diagnosis odds ratio.

## Introduction

Tuberculosis (TB) remains one of the most important and deadly infectious diseases globally, with 10.8 million new cases and 1.25 million deaths in 2023, and its global distribution is highly heterogenous [1]. The World Health Organization (WHO) has developed the End TB Strategy [2] and related TB (pre-)elimination frameworks [3] to support its ambitious targets in reducing TB incidence and mortality, emphasising systematic prevention and screening efforts [4] in addition to a wide range of other TB control tools.

There has been a long history of migrant TB screening, using a wide variety of different tests and algorithms. Numerous screening tests for TB are available, but most do not have high sensitivity or specificity, and in screening practice they are often combined. Most available algorithms are based on symptom screens and chest X-rays (CXRs), often followed by sputum smear or culture and, more recently, molecular tests such as GeneXpert (Cepheid, Sunnyvale, CA, USA). However, practice remains highly heterogenous [5], largely because of scarce evidence [6], policy priorities and differing migrant population flows. Therefore, screening programmes vary widely in their scope, target population, point of screening and tests and algorithms used.

Migrant screening for (active) TB is only conditionally WHO-recommended [7]. Associated screening tools, which include “using a symptom screen, chest X-ray or molecular WHO-recommended rapid diagnostic tests, alone or in combination” [7] are also conditionally recommended by the WHO owing to the very low certainty of evidence.

There is a significant and evolving body of literature, including several systematic reviews [8, 9], evaluating test properties for common screening tests and new evidence has become available since the last guideline iteration [7]. Sensitivity tends to be lower for symptom screens than for CXR abnormalities. Specificity for symptom screens also tends to be lower than for CXRs [9]. Single tests have suboptimal receiver operating characteristic (ROC) curves; therefore, significant trade-offs between high sensitivity/low specificity tests and low sensitivity/high specificity tests (*e.g.* CXR: any abnormality *versus* TB-specific abnormality) are apparent.

Previous studies have estimated test properties resulting from combinations of tests, seeking to optimise algorithms. Algorithms with CXR (any abnormality or TB-specific abnormalities) followed by Xpert MTB/RIF (used to identify *Mycobacterium tuberculosis* complex (MTB) and rifampicin (RIF) resistance) had the highest TB yields and lowest numbers needed to screen, whereas algorithms combining symptom screens (prolonged cough) with sputum smear or Xpert MTB/RIF had the lowest yields [8]. Setting is important in this context because of test availability and logistics and because TB prevalence among those screened will determine yield and positive predictive value (PPV) [8].

Traditionally, tuberculin skin tests (TSTs) and interferon-γ release assays (IGRAs), which measure the TB-specific immune response *via* delayed hypersensitivity or *via* enzyme-linked immunosorbent assay (ELISA) or spot tests (ELISPOT), were designed to diagnose TB infection (TBI). Their use for diagnosing TB disease has been limited, partly owing to concerns about false negatives, *e.g.* because of decreased immunocompetence. However, their role in establishing a TB diagnosis has been widely explored in the literature, particularly for diagnostically challenging forms of extrapulmonary TB, such as pleural or osteoarticular TB [10, 11] or paediatric TB [12]. Many of the original studies evaluated test properties of TST and IGRA among people with active TB as the reference group [13] because TBI is not directly measurable. In these studies, IGRAs have reasonably high sensitivities and specificities, which are lower among people living with HIV but still with reasonable diagnostic values [14].

We hypothesised that these “TBI tests” could add value if used in an algorithm of migrant TB disease screening. Additionally, there might be advantages in including them as an initial screening tool, thereby limiting the number of persons requiring CXRs and allowing TB and TBI screening programmes to be combined. In current screening practice, TBI test use is not uncommon, either as an early screening test for TB or in a combined TBI/TB programme, such as in Sweden [15] or Italy [16].

Significant TB reduction or even elimination [2] critically depends on optimising screening strategies for high-risk population, including migrants. Our study analyses TB screening algorithms for migrants from high to low TB incidence countries (*e.g.* Europe, North America, Australia), with a specific focus on how TBI tests could add value to these algorithms, by reviewing test properties and combining them in decision analytical modelling.

## Methods

In our two-part study, we first performed an umbrella review (systematic review of reviews (RoR)) to summarise and pool the evidence for the sensitivity and specificity of all relevant and commercially available tests used in TB screening to detect prevalent TB disease. Second, we estimated results for common test combinations using decision tree analysis.

### Review of reviews

We performed a systematic search of PubMed, Embase, Web of Science and the Cochrane Library as well as relevant grey literature, including conference abstracts and systematic review registers. We checked articles’ reference lists to ensure inclusion of all relevant articles. EPPI-Reviewer software [17] was used for screening and full-text review.

We included only systematic reviews (SRs) with extractable relevant test property values for all screening or confirmatory tests allowing detection of prevalent TB disease. Limiting inclusion to SRs allowed the simultaneous review of multiple tests and greater precision in updating pooled test properties. Given the migrant screening context, inclusion was restricted to an immunocompetent general population of all age groups (excluding children-only studies). We excluded studies with high HIV-positive populations (prevalence >30%) for the main analysis but assessed the effect of including these studies on test properties in sensitivity analyses.

Screening and confirmatory tests for prevalent TB disease included those commonly used and available, such as CXR or symptom screen, as well as IGRAs and TSTs. A full list of included tests is shown in the supplementary material.

No language restrictions were applied, but papers had to be indexed in English from 1 January 1966 to 1 September 2023. Full search terms and inclusion criteria are included in the supplementary material. Two researchers (D. Zenner and T. Chaudhry) independently screened titles and abstracts (stage 1), then full papers (stage 2), and discussed and agreed inclusion. Two other researchers (F. Cobelens and I. Abubakar) acted as arbiters in case no consensus was reached.

The outcome was pulmonary TB disease. Mostly, this was bacteriologically confirmed with little variation in the outcome definition in the original studies. SRs had to at least present either true positives (TPs) and false negatives (FNs) or true negatives (TNs) and false positives (FPs) for each underlying study to calculate sensitivity and specificity, respectively. If not available, we calculated these from sensitivity/specificity, if relevant denominators TP+FN or TN+FP were given or could be calculated (*e.g.* from positivity). We excluded SRs where these individual study test properties were not presented or could not be calculated (*e.g.* if only pooled results were given). We also excluded those SRs with double zero counts for the TP/FN or TN/FP pairs as noninformative. We extracted basic information from original papers, including study setting and population and quality information. Data extraction to a standardised template was carried out by either D. Zenner or T. Chaudhry and all extractions were cross-checked for accuracy by the other researcher. F. Cobelens and I. Abubakar acted as arbiters.

The original study quality was assessed by the SR authors, almost always using the QUADAS-2 tool [18] and we did not repeat a quality assessment of underlying studies. We only included SRs with clear and transparent search strategies that conformed to standard reporting guidelines.

Data were imported into Stata 16 (StataCorp, College Station, TX, USA), cleaned and deduplicated. We pooled test property data using slope/random intercept meta-analysis models for binomial distributions (Stata Metadta) [19]. Data are presented as summarised tables of sensitivities, specificities and diagnostic odds ratios (dORs) and graphically as forest plots and summary ROC curves. To assess unexpected between-study heterogeneity, we calculated I^2^ values as proposed for diagnostic studies by Zhou and Dendukuri [20] to allow for correlation between sensitivity and specificity.

Several tests have evolved during the observation time, *e.g.* there have been several generations of the QuantiFERON test (QIAGEN, Hilden, Germany). Although we present pooled test properties (sensitivity and specificity) for each of the diagnostic test results as the main outcome, we explored the analysis further through stratification. The RoR was registered on PROSPERO (CRD42023393129).

### Diagnostic pathway modelling

The second part of the study analysed the TB screening test algorithm in the context of migrant screening. We explored test properties across a range of practical and feasible two- or three-test combination screening pathway scenarios in decision analytical models considering a hypothetical population of 100 000 migrants, using the pooled results from the RoR. We simulated outcomes in terms of combined test properties, as well as the numbers of TB cases correctly identified through screening (TPs), TB cases missed by screening (FNs), people without TB who would be incorrectly diagnosed (FPs) and those correctly identified as not having TB (TNs). In addition, we calculated PPVs and negative predictive values for a plausible range of TB prevalence. The PPV of the test is a key prevalence-dependent statistic, which accounts for FPs and its inverse is a measure of efficiency for the algorithm, providing an estimate of the amount of diagnostic work-up to diagnose TB cases, reflecting potential costs.

Finally, we compared the different test combinations by calculating their positive and negative likelihood ratios and dORs. We summarise key outcomes of interest as 1) the proportion of true TB cases in the cohort detected (sensitivity) and 2) the number of individuals who require diagnostic work-up for TB to diagnose one true TB case (1/PPV). We also present 3) dORs (odds of diagnosis in patients with a positive test result over the odds of diagnosis in patients with a negative test result) as a measure of accuracy for the algorithms.

We explored the effect of parameter uncertainties on outcome measures through deterministic one-way sensitivity analyses, assuming these parameters to take a value at the extreme of their uncertainty intervals. In addition, we performed sensitivity analyses for test properties in settings with high and low HIV prevalence, for studies which included a high proportion of children, and for bacille Calmette–Guérin (BCG) status of the tested population. We also explored the effect on outcomes by varying all parameter values probabilistically using Monte Carlo simulation of repeated random sampling in a hypothetical cohort of 100 000 people to create a probabilistic random distribution for TP, FP, TN and FN values for each test combination. From this distribution we estimated the mean, standard deviation (sd), upper and lower bounds of 95% confidence intervals (CIs) and the minimum and maximum values for these. Using these estimates, we then calculated best- and worst-case scenarios for sensitivity, specificity and dORs to estimate the robustness of our results. We mathematically replaced zeros with small positive input values (*e.g.* for FN); therefore, estimates are conservative. For this modelling we assumed conditional independence of tests. Lastly, to mitigate the theoretical concern that TBI test specificity could be decreased among migrants from high TBI positivity areas (*i.e.* by having TBI not TB), we carried out an additional sensitivity analysis artificially decreasing specificity for all TBI tests by 16% (TBI positivity taken from the migrant TBI screening programme in England) [21].

Decision analytical modelling and calculation of dORs was performed with MS Excel (Microsoft Corp., Redmond, WA, USA) and Stata 16 (StataCorp).

## Results

### Review of reviews

**FIGURE 1:**
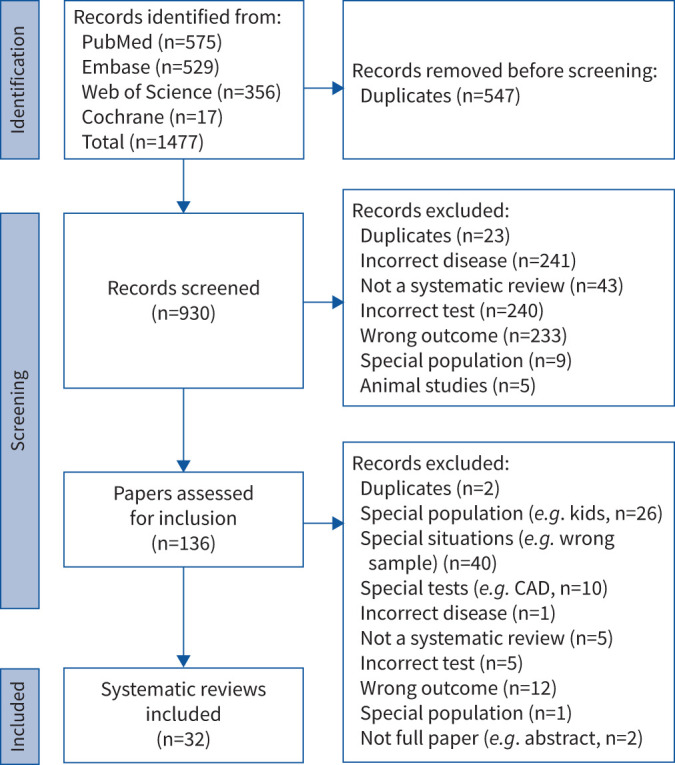
Preferred Reporting Items for Systematic Reviews and Meta-Analyses (PRISMA) chart: included and excluded studies from the review of reviews. CAD: computer-assisted diagnosis.

Our database search yielded 1477 records from the published literature. The search of grey literature and the hand-search of reference lists did not reveal additional records. We identified and removed duplicate records at different levels, using three-item combinations in EndNote (Philadelphia, PA, USA) before screening (n=547), manual removal during screening (n=23) and full-text review (n=2). We screened titles and abstracts of 930 papers and performed full-text review of 136 papers. We included 32 papers [9, 13, 22–51] for analysis (figure 1).

For the meta-analysis, data from underlying papers were extracted from 27 SRs [9, 13, 23, 25–29, 31–34, 36, 38–51]. Four SRs [22, 24, 30, 35] were excluded because data could not be extracted from individual studies and one SR was excluded [37] because it was superseded and largely overlapped with a newer SR from the same authors [13]. After deduplication, we included original data from 437 studies on test properties of 18 different tests, generating 692 unique rows of test property information. For the baseline analysis we excluded studies among high HIV prevalence populations (n=59).

We pooled data from all original studies included in SRs to estimate sensitivity and specificity, wherever possible (n=13 tests). Sensitivities range between 0.41 (95% CI 0.35–0.46) for prolonged (>2 weeks) cough and 1.00 (0.98–1.00) for parallel cough and CXR screening. Specificities ranged between 0.79 (95% CI 0.67–0.88) for the TST and 0.98 (95% CI 0.98–0.99) for Xpert (table 1).

**TABLE 1:** Pooled test property estimates

	Studies (n)	Estimate (95% CI)	I^2^
**Sensitivity**			
Cough >2 weeks	42	0.41 (0.35–0.46)	72.33
Any cough	22	0.50 (0.42–0.58)	73.92
CXR (any abnormality)	24	0.94 (0.91–0.96)	52.26
CXR (TB abnormality)	19	0.87 (0.78–0.92)	67.93
Cough and CXR (parallel)^#^	24	1.00 (0.98–1.00)	5.32
Any TB symptom	32	0.71 (0.63–0.78)	81.33
Xpert	139	0.88 (0.85–0.90)	70.37
Ultra	8	0.92 (0.83–0.97)	69.83
LAMP	14	0.92 (0.86–0.95)	82.93
SAT	4	0.98 (0.88–1.00)	52.59
TST	35	0.67 (0.58–0.75)	87.65
QuantiFERON	64	0.83 (0.79–0.86)	69.83
T-Spot.TB	46	0.88 (0.85–0.90)	66.46
**Specificity**			
Cough >2 weeks	42	0.95 (0.93–0.96)	97.91
Any cough	22	0.88 (0.82–0.92)	98.81
CXR (any abnormality)	24	0.89 (0.86–0.92)	99.57
CXR (TB abnormality)	19	0.96 (0.93–0.97)	92.5
Cough and CXR (parallel)^#^	24	0.84 (0.81–0.87)	99.38
Any TB symptom	32	0.68 (0.57–0.78)	99.54
Xpert	139	0.98 (0.98–0.99)	42.62
Ultra	8	0.96 (0.89–0.98)	69.82
LAMP	14	0.95 (0.91–0.97)	71
SAT	4	0.90 (0.79–0.96)	84.47
TST	35	0.72 (0.64–0.79)	91.05
QuantiFERON	64	0.90 (0.84–0.93)	69.79
T-Spot.TB	46	0.83 (0.78–0.87)	81.9

CI: confidence interval; CXR: chest X-ray; TB: tuberculosis; Xpert: Gene Xpert; Ultra: Gene Xpert Ultra; LAMP: loop-mediated isothermal amplification; SAT: simultaneous amplification and testing; TST: tuberculin skin test. ^#^: parallel cough and CXR screening requires both tests to be positive to lead to confirmatory tests. Parallel testing pooled studies with CXR (any abnormality) and CXR (TB suggestive abnormality).

For QuantiFERON we also provide a pooled estimate. However, there are several generations of tests available, therefore we undertook stratified analysis, providing pooled analysis for QuantiFERON Gold, QuantiFERON Plus and all others (table 2). Individual study results ordered by test, including forest plots and summary ROC plots, are included as supplementary figures S1–S26.

**TABLE 2:** Stratified test property estimates for different QuantiFERON generations

	Studies (n)	Estimate (95% CI)	I^2^
**QFT sensitivity**			
QFT Plus	8	0.94 (0.90–0.96)	16.88
QFT Gold	48	0.82 (0.78–0.86)	76.41
QFT (other)	19	0.79 (0.72–0.85)	74.00
**QFT specificity**			
QFT Plus	8	0.99 (0.95–1.00)	9.04
QFT Gold	48	0.87 (0.81–0.92)	80.11
QFT (other)	19	0.93 (0.85–0.97)	71.35

CI: confidence interval; QFT: QuantiFERON.

Heterogeneity, particularly for specificity, was relatively high. We therefore stratified the analysis by BCG vaccination status (for TST, table 3), HIV prevalence and TB incidence in the tested population (supplementary tables S2–S4). This led to lower heterogeneities for pooled specificities in TST, QuantiFERON, T-Spot.TB, LAMP, Xpert and Ultra, but not for clinical and radiological screening tests.

**TABLE 3:** Stratified test property estimates for TST by BCG coverage of the populations

	Studies (n)	Estimate (95% CI)	I^2^
**TST sensitivity**			
BCG positive^#^	28	0.62 (0.53–0.71)	86.79
BCG negative^¶^	2	0.82 (0.75–0.88)	N/A
All	35	0.67 (0.58–0.75)	87.65
**TST specificity**			
BCG positive	28	0.71 (0.63–0.78)	88.24
BCG negative^¶^	2	0.98 (0.97–0.99)	N/A
All	35	0.72 (0.64–0.79)	91.05

CI: confidence interval; TST: tuberculin skin test; BCG: vaccination with Bacille Camille-Guérin; N/A: not applicable. ^#^: ≥30% vaccinated; ^¶^: <30% vaccinated; excluded if information not provided.

Funnel plots and Deeks’ funnel plot asymmetry tests showed no evidence of publication bias for any test, except for T-SPOT.TB (p<0.001, supplementary figures S27–S39).

### Diagnostic pathway modelling

**FIGURE 2:**
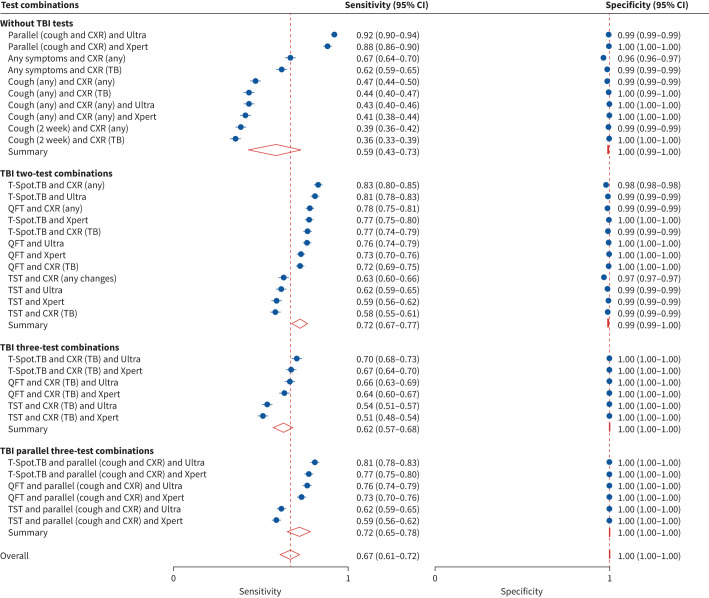
Forest plot of sensitivity and specificity (with 95% confidence intervals), overall and stratified for two-test and three-test combinations with or without tuberculosis infection (TBI) test. CXR: chest X-ray; Ultra: Gene Xpert Ultra; Xpert: Gene Xpert; TB: tuberculosis; QFT: QuantiFERON; TST: tuberculin skin test.

Model outputs of test properties for common and feasible two-test or three-test combinations (n=34) are summarised in figure 2.

Most combinations are sequential (to mirror current programmes), except for the parallel CXR/symptom (cough) screen, which can be combined with follow-up tests. Almost all algorithms achieved high specificities, apart from those containing “any TB symptom” or “any CXR abnormality”. However, algorithms varied widely in their sensitivity (their ability to correctly detect true cases). Algorithms with IGRAs and those with a parallel CXR/symptom screen as the initial screening test had the highest sensitivities (figure 2). Examples of these were combinations with parallel (cough/CXR) screening followed by Xpert (0.88, 95% CI 0.86–0.90) or Ultra (0.92, 95% CI 0.90–0.94) as well as T-Spot.TB followed by CXR (0.83, 95% CI 0.80–0.85) or Ultra (0.81, 95% CI 0.78–0.83) and T-Spot.TB followed by parallel symptom/CXR screening and either Ultra (0.81, 95% CI 0.78–0.83) or Xpert (0.77, 95% CI 0.75–0.80).

**FIGURE 3:**
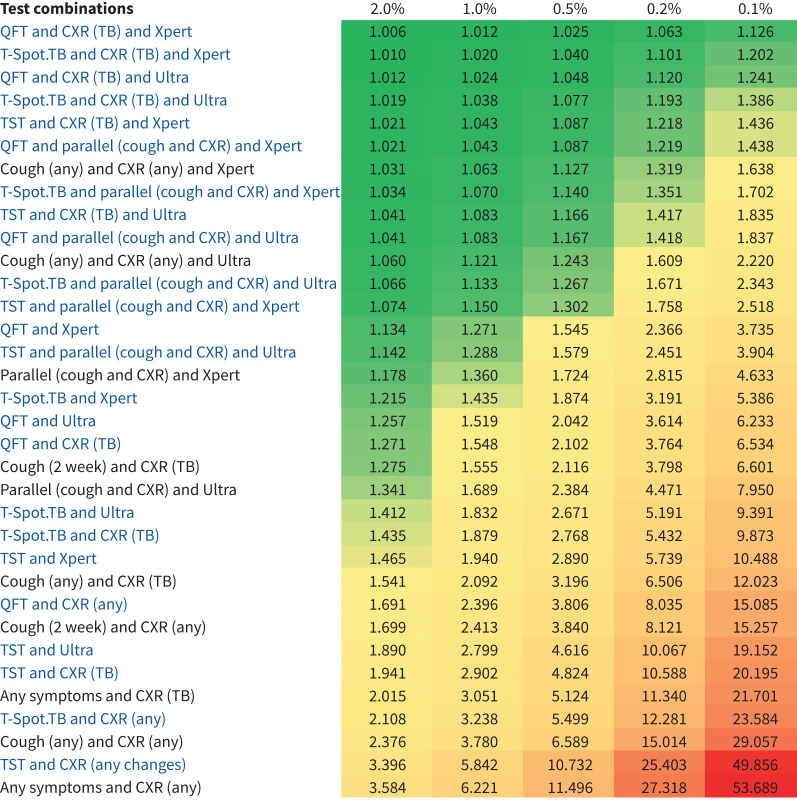
Inverse positive predictive value (iPPV) for different prevalence scenarios. The iPPV can be interpreted as the number of individuals who require diagnostic work-up for tuberculosis (TB) to diagnose one true TB case. Chart and legend ordered by iPPV at a prevalence of 0.1% (100 per 100 000) and display iPPV according to prevalence categories of (from left to right) 2000, 1000, 500, 200 and 100 per 100 000. Tests with a TBI test are in blue font. QFT: QuantiFERON; CXR: chest X-ray; Xpert: Gene Xpert; Ultra: Gene Xpert Ultra; TST: tuberculin skin test.

The top six combinations with the highest PPVs (or lowest inverse PPV) included a TBI test as the initial step (figure 3). Although all PPV estimates were highly prevalence-dependent, variation was less pronounced for TBI-containing algorithms than for those without TBI testing (supplementary figure S41 and table S7).

We calculated positive and negative likelihood ratios and resulting dORs, which allow direct comparison of algorithms accuracies. We compared dORs (figure 4) and plotted summary operating characteristics for all tests (supplementary figures S1–S27). Three-test combinations had significantly higher dORs than two-test combinations. Very high dORs were estimated for the scenarios based on an IGRA confirmed by CXR and Xpert.

**FIGURE 4:**
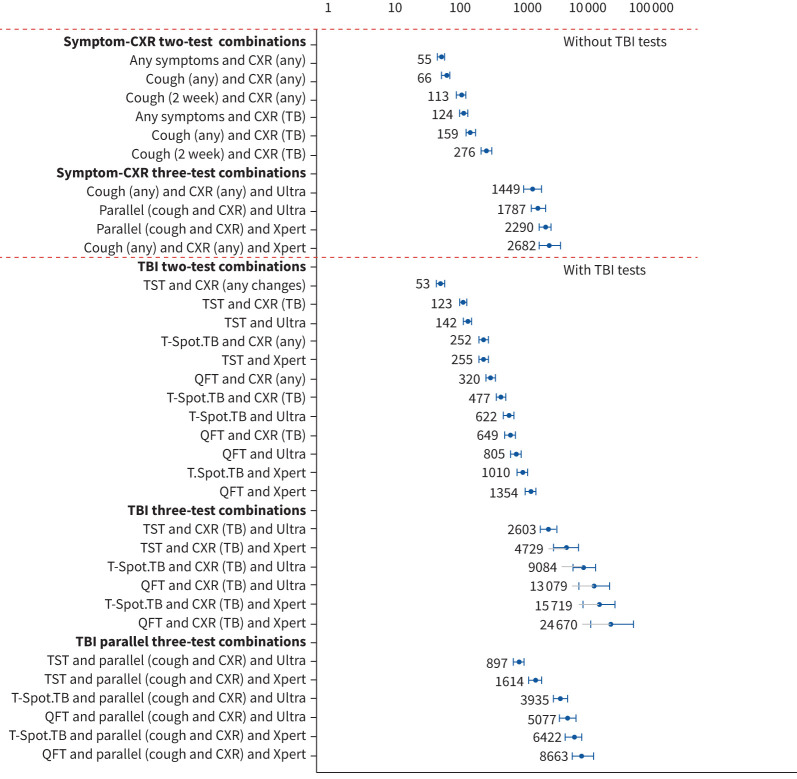
Diagnostic odds ratios (dORs) for two-test and three-test combinations with 95% confidence intervals. Stratified by algorithms by number and type of test, with and without tuberculosis infection (TBI) tests. Ordered by dOR within categories. Logarithmic scale. CXR: chest X-ray; TB: tuberculosis; Ultra: Gene Xpert Ultra; Xpert: Gene Xpert; TST: tuberculin skin test; QFT: QuantiFERON.

Our probabilistic sensitivity analysis demonstrated that using worst- and best-case assumptions, sensitivity will vary by around 4.5% for high sensitivity tests (*e.g.* parallel cough/CXR plus Ultra), estimated at 92% (varying between 89.5% and 94.1%), and by less for low sensitivity tests (supplementary tables S9 and S10). Our additional TBI-focused sensitivity analysis decreased dOR estimates considerably, but not our overall finding that TBI tests add value to the algorithm (supplementary table S11).

## Discussion

We used a RoR to estimate properties of 13 tests to diagnose active TB. A decision analytical modelling approach for 34 two- and three-test algorithms using these estimates demonstrated that adding TBI tests to two- or three-test algorithms significantly improved overall test performance with high sensitivities and increased PPVs, reducing the number of people screened who require diagnostic work-up for TB in a non-immunocompromised population. All top nine combinations by dOR and the six combinations with highest sensitivity included a TBI test (usually IGRA), followed by a CXR and a molecular test (Xpert or Ultra). The combinations of QuantiFERON or T.Spot.TB followed by CXR (any changes) and Xpert had the highest dORs and PPVs and the lowest numbers of FPs. Based on our analysis, inclusion of an IGRA as an initial test in screening algorithms to detect TB in migrants increased dORs significantly and for some combinations exponentially.

The excellent sensitivities of TBI two-test combinations are worth noting; however, these combinations yield a higher number of FPs, leading to lower dORs and PPVs. The resulting higher costs and inconvenience to migrants from additional investigations and treatment in three-test combinations need to be balanced against practicalities and cost-savings from these less complex algorithms.

There is a surprising scarcity of literature evaluating the accuracy of TB test algorithms, particularly in the context of systematic migrant screening; this could explain the current lack of specificity and confidence around WHO test recommendations [7]. Conceptual early work emphasised the importance of ensuring high test sensitivity to establish low numbers needed to screen, and high specificity in follow-up tests to avoid FNs [52]. Van't Hoog *et al.* [8] considered a range of algorithms, mostly combining a symptom or CXR screen with confirmation by smear or Xpert, and found significant variation and uncertainty around estimates for numbers needed to screen and PPV. Our work expands on this by including IGRA tests and newer generation molecular testing, which has not yet been addressed in the literature. A protocol for inclusion of TBI testing in a network meta-analysis on TB screening algorithms in the prison setting has been published, but results were not yet available at time of writing [53].

There have been two important recent developments, which were outside the scope of this review. New-generation TSTs have been developed, including the Cy-Tb (Serum Institute of India, https://mylabglobal.com/cy-tb/) and the Diaskintest (Central Tuberculosis Research Institute, Moscow, Russia) [54]. The commercial availability and, hence, programme application of these tests is currently limited and regional, a key reason for not including these. Based on the same antigens used in IGRAs (ESAT-6 and CFP10), these tests are expected to have very similar properties to IGRAs; a recent systematic review confirms high agreement between these tests [54]. The modelled algorithm properties of novel skin tests will be similar to IGRA tests, and the decision regarding the test will depend on other context/setting-specific factors, *e.g.* available resources and infrastructure. Depending on these logistic considerations, novel skin tests could therefore also be included in our algorithms as initial screening tests, similar to IGRAs.

The other important development is computer-aided detection for TB (CAD-TB). Whilst potentially exciting from a pragmatic and resource perspective [55], CAD-TB diagnostic studies are limited by high variability in design and outcome (resulting in high heterogeneity), can vary by settings and populations [56], and overall diagnostic accuracy remains inferior to standard CXR reading. The initially inferior diagnostic accuracy of CAD-TB [57] has shown improvement over the years [58], with sensitivities now similar to human readings but with trade-offs in specificity. Indeed, a recent individual patient data meta-analysis demonstrated that, at developer-recommended thresholds, sensitivities for tested products were similar to human reading (84–87.7%) but with considerably worse specificities (59.2–69.1%) [59]. Exploring significant trade-offs in ROC curves, they estimated specificities to range between 54.1% and 60.5% if holding sensitivity at 90%. The use of CAD-TB in migrant screening will therefore be setting-specific with important choices around resource allocation for FPs. However, this is a fast-evolving field with applied machine-learning having potentially significant benefits for the future [60].

The choice of screening test and algorithm depends on many factors, including TB prevalence, the screening population and setting, test availability and pricing, and available resources, among others, some of which we explore elsewhere [61]. We included a range of prevalence scenarios in our paper, but we did not include cost or cost-effectiveness considerations or those relating to acceptability or feasibility in specific settings. Costs of TBI tests can vary widely and depend on many factors, including local material and staff costs, as well demonstrated in a novel costing framework [62]. Increasingly, decision aids for local policy-makers are available [63], and despite usually limited scope, they help in visualising choices. Adding our evidence to update these tools would expand the range of available screening tools and allow modelling of additional TBI screening. For example, our model could be added to the ScreenTB tool [63] with a box to add local cost and disease prevalence data and predict cost-effectiveness based on dORs and PPVs.

Our study has a number of strengths and limitations. Our RoR methodology allowed the vast amount of literature on 18 tests to be reviewed, and our individual study extraction minimised the effect of inter-study duplication. It is possible that studies not included in the SRs may have been omitted, and the limited number of studies for some tests (*e.g.* smears) affected our ability to provide pooled results. However, it is unlikely that point estimates of pooled results have been significantly affected by this, given the vast number of individuals these are based on. Our modelling does not directly account for prevalence-related changes in test properties (*e.g.* IGRA specificity); however, our extensive sensitivity analysis provides a range of reasonable worst- and best-case scenarios. Study quality had been assessed by the SRs; however, we did not perform sensitivity analysis using this information.

We present stratified analyses by HIV prevalence, BCG status and TB incidence, improving study heterogeneity; however, there was residual heterogeneity for specificity estimates of symptom and CXR tests. This well-described issue [9] is likely the result of pooling test property estimates from TB prevalence surveys from a wide range of contexts and there were no specific characteristics allowing for further stratification. For example, there could be ascertainment bias in data from prevalence surveys, potentially leading to overestimated pooled sensitivity of parallel symptom/CXR screening (100%). Our data stem from diagnostic accuracy studies, which can overestimate test properties compared to routine practice. Lastly, there was some variation in outcome definitions of underlying studies (supplementary table S1); however, additional analysis limiting studies to culture-confirmed TB produced compatible effect estimates. RoRs and SRs are always limited by underlying studies, and we explored the effect of varying test property estimates in probabilistic sensitivity analysis.

In conclusion, our updated TB test property estimates and our modelling ranked and quantified combined test properties in common screening algorithms. We demonstrated a significant test accuracy benefit of adding IGRAs early to an active TB screening pathway by improving specificity and PPV without compromising sensitivity, by increasing efficiency (*i.e.* fewer migrants may need additional tests), and with the additional advantage of optionally adding TBI screening to the algorithm. Such early IGRA inclusion is consistent with the new WHO TBI guidance for the TB preventive treatment algorithm [64], updating the International Union Against Tuberculosis and Lung Disease clinical standards with *a priori* TB disease exclusion [65]. TBI tests could also support earlier diagnosis of harder to diagnose TB, including extrapulmonary TB or TB in children. Routine use of TBI tests in migrant screening will depend on operational, logistical and resource considerations; to facilitate these decisions, this could be added to a pragmatic tool to inform policy-makers [63]. Although the landscape of TB tests is constantly changing, and new evidence may change the values, our approach and methodology will allow the rapid recalculation of values and could therefore be adapted for new tests and pathways in the future.

## Shareable PDF

10.1183/13993003.02000-2024.Shareable1 This PDF extract can be shared freely online.
Shareable PDF

ERJ-02000-2024.Shareable

## Supplementary material

**Please note:** supplementary material is not edited by the Editorial Office, and is uploaded as it has been supplied by the author. Supplementary material ERJ-02000-2024.Supplement
