# Supplementary material for: How to diagnose TB in migrants? A systematic review of reviews and decision tree analytical modelling exercise to evaluate properties for single and combined tuberculosis screening tests
Source: Eur Respir J. 2025 Jul 24;66(1):2402000. doi: 10.1183/13993003.02000-2024 (PMC12287609; doi:10.1183/13993003.02000-2024)
Supplement: Supplementary file 1 [file ERJ-02000-2024.Shareable.pdf]

EUROPEAN RESPIRATORY JOURNAL
REVIEW


# How to diagnose TB in migrants? A systematic review of reviews and decision tree analytical modelling exercise to evaluate properties for single and combined tuberculosis screening tests

Dominik Zenner, Hassan Haghparast-Bidgoli, Tahreem Chaudhry, Ibrahim Abubakar and Frank Cobelens

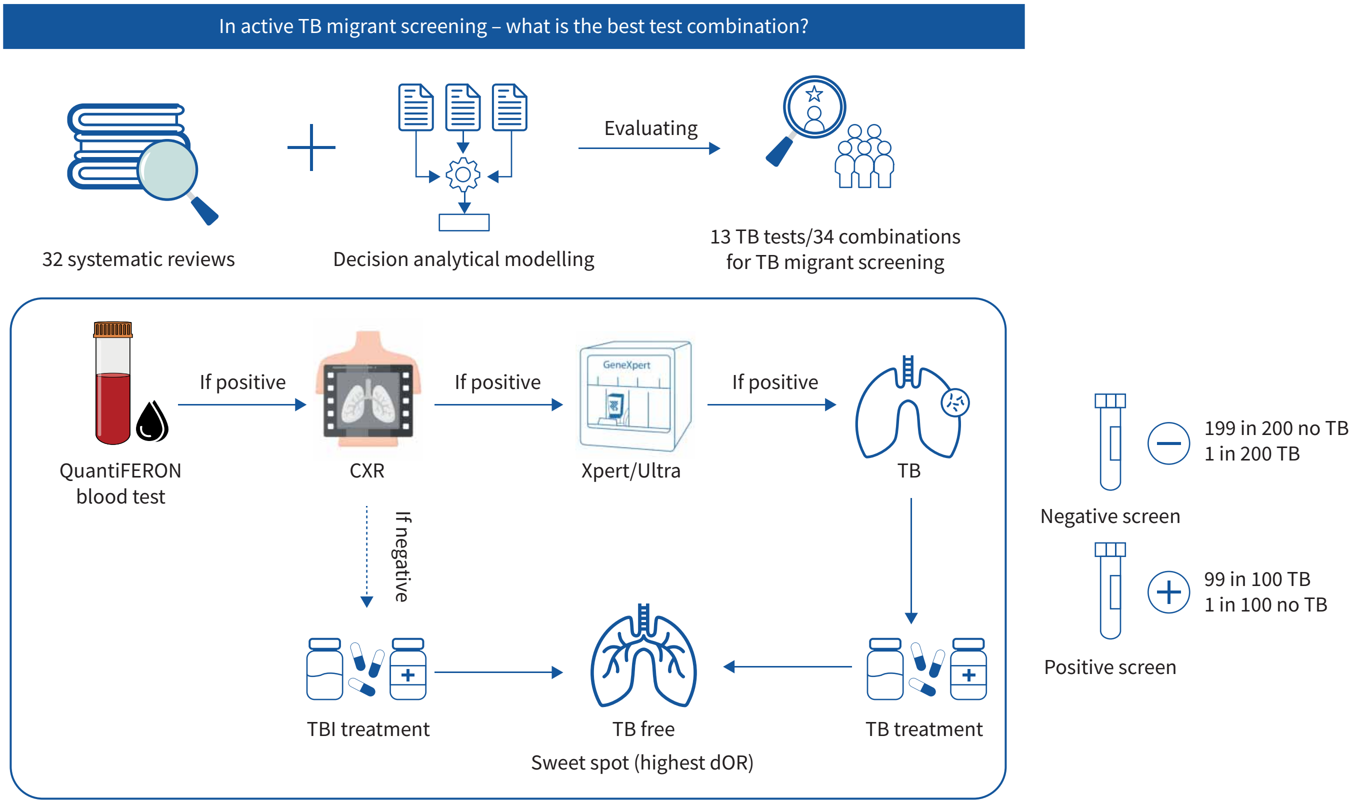


**GRAPHICAL ABSTRACT** Overview of the study. TB: tuberculosis; CXR: chest X-ray; dOR: diagnosis odds ratio.

SHAREABLE PDF

# How to diagnose TB in migrants? A systematic review of reviews and decision tree analytical modelling exercise to evaluate properties for single and combined tuberculosis screening tests

Dominik Zenner[1,2,3,4], Hassan Haghparast-Bidgoli[2], Tahreem Chaudhry[1], Ibrahim Abubakar[2] and Frank Cobelens[3]

[1]Wolfson Institute of Population Health, Queen Mary University of London, London, UK. [2]Institute for Global Health, University College London, London, UK. [3]Amsterdam University Medical Centers, location Universiteit of Amsterdam, Department of Global Health, Amsterdam Institute for Global Health and Development, Amsterdam, the Netherlands. [4]Queen Mary and Barts Health Tuberculosis Centre, Faculty of Medicine and Dentistry, Queen Mary University of London, London, UK.

Corresponding author: Dominik Zenner (d.zenner@qmul.ac.uk)

Check for updates

Shareable abstract (@ERSpublications)
**There is a significant test accuracy benefit of adding IGRAs to an active TB screening pathway, which will help inform clinicians and policy-makers on the most effective screening algorithms** https://bit.ly/3EgtJ4R

**Cite this article as:** Zenner D, Haghparast-Bidgoli H, Chaudhry T, *et al*. How to diagnose TB in migrants? A systematic review of reviews and decision tree analytical modelling exercise to evaluate properties for single and combined tuberculosis screening tests. *Eur Respir J* 2025; 66: 2402000 [DOI: 10.1183/13993003.02000-2024].

This PDF extract can be shared freely online.

Copyright ©The authors 2025.

This version is distributed under the terms of the Creative Commons Attribution Licence 4.0.

This article has an editorial commentary: https://doi.org/10.1183/13993003.00750-2025

Received: 8 Oct 2024
Accepted: 11 March 2025

## Abstract

***Background*** Optimising tuberculosis disease testing algorithms is fundamental to ensuring the effectiveness and cost-effectiveness of migrant screening programmes, including better understanding of individual and combined screening test properties. The aim of our study was to estimate pooled tuberculosis test properties from the literature and combine them in decision analytical modelling with a focus on whether tests used for the diagnosis of tuberculosis infection might add value to these algorithms.

***Methods*** We performed a systematic review of reviews of diagnostic tests for active tuberculosis, searching PubMed, Embase, Web of Science and Cochrane library, and pooled test properties extracted from original papers included in reviews. We used these pooled results in a decision tree analysis to estimate test properties for common migrant screening algorithms.

***Results*** We retrieved 1477 records and included 32 reviews, including data from 437 original studies for 18 tuberculosis tests, providing pooled results for 13. Our modelling showed that algorithms with interferon-γ release assays had the highest diagnostic odds ratios (dORs) (*e.g.* QuantiFERON/chest X-ray (for tuberculosis abnormalities)/Xpert dOR 24 670, 95% CI 11 630–52 328) and high positive predictive values. Best sensitivities were achieved for combinations with parallel cough/chest X-ray screening followed by Xpert (0.88, 95% CI 0.86–0.90) or Ultra (0.92, 95% CI 0.90–0.94) as well as by T-Spot.TB followed by parallel symptom/chest X-ray screening and Ultra (0.81, 95% CI 0.78–0.83) or Xpert (0.77, 95% CI 0.75–0.80).

***Conclusions*** The significant test accuracy benefit of adding interferon-γ release assays to an active tuberculosis screening pathway will help inform clinicians and policy-makers on the most effective screening algorithms.
