# Supplementary material for: How to diagnose TB in migrants? A systematic review of reviews and decision tree analytical modelling exercise to evaluate properties for single and combined tuberculosis screening tests
Source: Eur Respir J. 2025 Jul 24;66(1):2402000. doi: 10.1183/13993003.02000-2024 (PMC12287609; doi:10.1183/13993003.02000-2024)
Supplement: Supplementary file 2 [file ERJ-02000-2024.Supplement.pdf]

Supplement

Table of Contents

Summary table of included systematic reviews (Table 1) 2
Definitions 6
Tests to detect TB disease 7
Search strategy 8
Additional information regarding meta-analysis 10
Forest plots and Summary receiver operating characteristic plots 11
Funnel plots by screening test 37
Pooled Test results – stratified analysis...44
Summary receiver operating characteristic for test combinations. 47
Combined Test properties 48
PPVs and NNS for different prevalence scenarios 50
Probabilistic Sensitivity analysis 53

Summary table of included systematic reviews (Table S1)

author	year	outcome	TB incidence setting	age groups	HIV status	test	quality assessment	population
**Aggarwal**	2022	no extractable data						
**Assefa**	2019	Confirmed PTB (culture or mWRD)	high	mixed including adults and children	mixed including low and high	cough_2wk, cough_any, tbsx_any, cxr_any, cxr_tb,	QUADAS2	wide inclusion, but only cross-sectional methodology
**Chang**	2010	no extractable data						
**Dai**	2012	PTB (bacteriological)	mixed, mostly low	mixed including adults and children	mixed including low and high	qft, tspot	none	wide inclusion criteria, not specifically screening
**De Kyser**	2014	TB*	mixed low and high	mixed including adults and children	low	qft, tspot and tst	none	wide inclusion criteria, not specifically screening. Mainly case control studies
**Diel**	2011	PTB (culture or mWRD)	mixed, mostly low	mixed including adults and children	low	tspot	QUADAS2	wide inclusion criteria, not specifically screening.
**Horne**	2019	PTB (culture)	mixed, mostly high	adults (15+)	mixed including low and high	xpert	QUADAS2	wide inclusion criteria

**Huang**	2016	culture neg PTB	high (China only)	adults	low	tspot	QUADAS2	only Chinese studies
**Kahvati**	2016	no extractable data					USPSTF Quality Assessment tool	
**Kaur**	2016	PTB (bacteriological)	mixed, mostly high	mixed including adults and children	mixed including low and high	xpert	Risk of Bias	wide inclusion, mostly cross sectional
**Li**	2017	PTB (culture or composite reference standard)	mixed, mostly high	mixed including adults and children	mixed including low and high	xpert	QUADAS2	wide inclusion
**Lu**	2016	TB (author defined)	mixed, low and high	adults	mixed including low and high	qft, tspot and tst	Risk of bias	wide inclusion
**Metcalfe**	2011	no extractable data						
**Oh**	2020	TB (Culture, mWRD or predefined clinical)	mixed, mostly low	Predominantly adults	low	qft, tspot and tst	QUADAS2	wide, but more specific for specificity
**Pai**	2008	PTB (culture)	mixed, few high, majority low	mixed mostly adults, some children	low	qft, tspot and tst	none?	wide but excluding immuno-compromised
**Pai**	2004	superseded by Pai 2008			low			
**Petnak**	2022	smear neg TB	mixed, most high	15+	low	qft, tspot	QADAS2	wide inclusion

**Pourakbari**	2019	TB (culture or composite ref standard)	low	adults	low	qft	QADAS2	wide inclusion
**Rahmati**	2022	TB (culture)	low	where recorded, adults	low	smear and PCR	STARD checklist	only Iranian studies
**Sester**	2011	PTB (culture, mWRD)	mixed, low and high (SA most represented	mixed mostly adults, some children	low	qft, tspot and tst	QADAS2	wide inclusion
**Shapiro**	2021	PTB (culture)	high	mixed mostly adults, some children	mixed, mostly low	xpert	QADAS2	wide inclusion
**Shete**	2019	PTB (culture)	high	adults	mixed including low and high	LAMP	QADAS2	only high incidence
**Sotgiu**	2019	TB (culture or clinical confirmed)	low	where recorded, adults	low	qft	QADAS2	wide inclusion
**Steingart**	2014	culture confirmed PTB	mixed low and high	adults	low	xpert	QADAS2	wide inclusion
**van't Hoog**	2022	PTB (bacteriologically confirmed)	mixed low and high	Predominantly adults	low	cough_any, cough_2wk, tb_sx_any, parallel_cxr_cough, cxr_any, cxr_tb	QADAS2	wide inclusion
**Walusimbi**	2013	culture confirmed smear neg TB	mixed low and high	mixed mostly adults, some children	low	xpert	QADAS2	wide, only smear neg

**Wang**	2019	TB (culture or author defined composite standard)	mixed low and high	mixed mostly adults, some children	low	abott_rt	QADAS2	wide inclusion
**Yan**	2016	culture positive PTB	mixed, most high	mixed mostly adults, some children	low	xpert, LAMP, SAT	QADAS2	wide inclusion
**Zhang**	2023	culture positive PTB	mixed, most low	mixed, mainly adults, some adolescents	low	qft, tspot	QADAS2	wide inclusion
**Zhang**	2020	PTB (well-defined ref standard)	mixed, low and high	adults	low	xpert, ultra	QADAS2	wide inclusion
**Zifodya**	2021	PTB (culture confirmed or composite reference standard)	high	mixed mostly adults, some children	low	xpert, ultra	QADAS2	wide inclusion

*not further defined)

Definitions

Test properties: This includes the sensitivity, specificity, positive and negative predictive values of a given screening test. The reference standard for the meta-analysis is pulmonary Tuberculosis (TB), usually microbiologically or bacteriologically confirmed TB. The negative reference standard (controls) is healthy individuals without suspicion or confirmation of TB.
Sensitivity: probability that individuals with TB are correctly identified as such. Denoted as TP/(TP+FN)
Specificity: probability that individuals without TB are correctly identified as such. Denoted as TN/(TN+FP)
Positive Predictive Value: probability that individual with positive test has TB. Denoted as TP/(TP+FP)
Negative Predictive Value: probability that individual with negative test doesn't have TB. Denoted as TN/(TN+FN)
Diagnostic Odds Ratio (dOR): measure of effectiveness for diagnostic tests, comparing the odds of positive test among those with disease with odds of negative test among those without the disease. Denoted as (TP*TN)/(FP*FN) or LR+/LR-
Positive Likelihood Ratio (LR+): Probability of a positive test in a person with TB divided by the probability of a positive test in a person without TB. Denoted as Sens./(1-Spec.)
Negative Likelihood Ratio (LR-): Probability of a negative test in a person with TB divided by the probability of a negative test in a person without TB. Denoted as (1-Sens.)/Spec.
TBI tests: Tests for TB infection – this includes QantiFERON T-Spot.TB and Tuberculin Skin test (TST).
TST: Tuberculin Skin Test: A intradermally applied TBI test stimulating a delayed hypersensitive reaction with purified protein derivative from mycobacterial antigens. The test is "read" after 48-72 hours by measuring the size of the induration.
IGRAs: interferon gamma release assays. These are blood tests which detect the Interferon Gamma IFN-γ released if cells are stimulated with the ESAT-6 and antigens CFP-10 antigens from mycobacterium tuberculosis. They include different generations of QantiFERON and T-Spot.TB wich detect IFN-γ by enzyme-linked immunosorbent assay (ELISA) or enzyme-linked immunosorbent spot (ELISpot) respectively and are normally used to detect TB infection (TBI)
Xpert MTB/RIF (abbreviated Xpert): An automated, portable nucleic amplification test for rapid TB diagnosis
Xpert Ultra (abbreviated Ultra) A newer generation automated, portable nucleic amplification test for rapid TB diagnosis
mWRD: molecular WHO-recommended rapid diagnostic test (includes Xpert and Ultra).
Microbiologically confirmed: TB confirmed by microbiological culture
Bacteriologically confirmed: TB confirmed by microbiological culture or positive smear

Tests to detect TB disease

Classic tests used to detect TB disease

- Chest X-Ray (CXR)
  - any CXR abnormality
  - TB-specific CXR abnormality
- Symptoms
  - Any TB symptoms
  - Prolonged cough
- ZN/ Auramine Smear
- Xpert MTB/RIF
- Xpert MTB/RIF Ultra

Tests traditionally used for TB infection

- Tuberculin Skin Test (TST)
  - Stratification by different cut-off levels
- Interferon Gamma Release Assays
  - QuantiFERON (stratification by different generations)
  - T-Spot.TB

Search strategy

PubMed

Search: **(diagnost* OR Quanti* OR T-Spot OR TST OR Tuberculin OR xpert OR ultra OR CXR OR Chest X-Ray OR symptom OR cough) AND (Tuberculosis OR TB) AND (test propert* OR sensitivity OR specificity) NOT (immunosuppress*)** Filters: **Systematic Review**
((("diagnost*"[All Fields] OR "quanti*"[All Fields] OR "T-Spot"[All Fields] OR "TST"[All Fields] OR ("tuberculin"[MeSH Terms] OR "tuberculin"[All Fields] OR "tuberculination"[All Fields] OR "tuberculine"[All Fields] OR "tuberculinic"[All Fields] OR "tuberculins"[All Fields]) OR "xpert"[All Fields] OR "ultra"[All Fields] OR "CXR"[All Fields] OR (("chested"[All Fields] OR "thorax"[MeSH Terms] OR "thorax"[All Fields] OR "chest"[All Fields] OR "chests"[All Fields]) AND ("diagnostic imaging"[MeSH Subheading] OR ("diagnostic"[All Fields] AND "imaging"[All Fields]) OR "diagnostic imaging"[All Fields] OR "x ray"[All Fields] OR "x rays"[MeSH Terms] OR "x rays"[All Fields])) OR ("diagnosis"[MeSH Subheading] OR "diagnosis"[All Fields] OR "symptoms"[All Fields] OR "diagnosis"[MeSH Terms] OR "symptom"[All Fields] OR "symptom s"[All Fields] OR "symptomes"[All Fields]) OR ("cough"[MeSH Terms] OR "cough"[All Fields] OR "coughing"[All Fields] OR "coughs"[All Fields] OR "coughed"[All Fields])) AND ("tuberculosi"[All Fields] OR "tuberculosis"[MeSH Terms] OR "tuberculosis"[All Fields] OR "tuberculoses"[All Fields] OR "tuberculosis s"[All Fields] OR "TB"[All Fields]) AND ((("research design"[MeSH Terms] OR ("research"[All Fields] AND "design"[All Fields]) OR "research design"[All Fields] OR "test"[All Fields]) AND "propert*"[All Fields]) OR ("sensitive"[All Fields] OR "sensitively"[All Fields] OR "sensitives"[All Fields] OR "sensitivities"[All Fields] OR "sensitivity and specificity"[MeSH Terms] OR ("sensitivity"[All Fields] AND "specificity"[All Fields]) OR "sensitivity and specificity"[All Fields] OR "sensitivity"[All Fields]) OR ("sensitivity and specificity"[MeSH Terms] OR ("sensitivity"[All Fields] AND "specificity"[All Fields]) OR "sensitivity and specificity"[All Fields] OR "specificity"[All Fields] OR "specific"[All Fields] OR "specifically"[All Fields] OR "specification"[All Fields] OR "specifications"[All Fields] OR "specificities"[All Fields] OR "specifics"[All Fields] OR "specifities"[All Fields] OR "specifity"[All Fields]))) NOT "immunosuppress*"[All Fields]) AND (systematicreview[Filter])
Translations
Tuberculin: "tuberculin"[MeSH Terms] OR "tuberculin"[All Fields] OR "tuberculination"[All Fields] OR "tuberculine"[All Fields] OR "tuberculinic"[All Fields] OR "tuberculins"[All Fields]
Chest: "chested"[All Fields] OR "thorax"[MeSH Terms] OR "thorax"[All Fields] OR "chest"[All Fields] OR "chests"[All Fields]
X-Ray: "diagnostic imaging"[Subheading] OR ("diagnostic"[All Fields] AND "imaging"[All Fields]) OR "diagnostic imaging"[All Fields] OR "x ray"[All Fields] OR "x-rays"[MeSH Terms] OR "x-rays"[All Fields]
symptom: "diagnosis"[Subheading] OR "diagnosis"[All Fields] OR "symptoms"[All Fields] OR "diagnosis"[MeSH Terms] OR "symptom"[All Fields] OR "symptom's"[All Fields] OR "symptomes"[All Fields]
cough: "cough"[MeSH Terms] OR "cough"[All Fields] OR "coughing"[All Fields] OR "coughs"[All Fields] OR "coughed"[All Fields]
Tuberculosis: "tuberculosi"[All Fields] OR "tuberculosis"[MeSH Terms] OR "tuberculosis"[All Fields] OR "tuberculoses"[All Fields] OR "tuberculosis's"[All Fields]

test: "research design"[MeSH Terms] OR ("research"[All Fields] AND "design"[All Fields]) OR "research design"[All Fields] OR "test"[All Fields]
sensitivity: "sensitive"[All Fields] OR "sensitively"[All Fields] OR "sensitives"[All Fields] OR "sensitivities"[All Fields] OR "sensitivity and specificity"[MeSH Terms] OR ("sensitivity"[All Fields] AND "specificity"[All Fields]) OR "sensitivity and specificity"[All Fields] OR "sensitivity"[All Fields]
specificity: "sensitivity and specificity"[MeSH Terms] OR ("sensitivity"[All Fields] AND "specificity"[All Fields]) OR "sensitivity and specificity"[All Fields] OR "specificity"[All Fields] OR "specific"[All Fields] OR "specifically"[All Fields] OR "specification"[All Fields] OR "specifications"[All Fields] OR "specificities"[All Fields] OR "specifics"[All Fields] OR "specifities"[All Fields] OR "specifity"[All Fields]

575 unique records

Embase

#1 ((diagnostic OR ‘quantiferon’ OR 't spot'/exp OR 't spot' OR tst OR 'tuberculin'/exp OR tuberculin OR 'xpert'/exp OR xpert OR 'ultra'/exp OR ultra OR cxr OR 'chest x-ray'/exp OR 'chest x-ray') OR 'symptom'/exp OR symptom OR 'cough'/exp OR cough) AND ('tuberculosis'/exp OR tuberculosis OR 'tb'/exp OR tb) AND ('sensitivity'/exp OR sensitivity OR 'specificity'/exp OR specificity) NOT immunosuppress

#2 #1 AND ‘systematic review’

529 unique records

WoS

ALL=(diagnostics OR Quantiferon OR T-spot OR TST OR Tuberculin OR xpert OR ultra OR CXR OR Chest X-Ray OR symptom OR cough) AND ALL=(Tuberculosis OR TB) AND ALL=(test property OR sensitivity OR specificity) NOT ALL=(immunosuppress) AND ALL=(systematic review)

356 unique records

Cochrane
(diagnostics OR Quantiferon OR T-spot OR TST OR Tuberculin OR xpert OR ultra OR CXR OR Chest X-Ray OR symptom OR cough) AND (Tuberculosis OR TB) AND (test property OR sensitivity OR specificity) NOT (immunosuppress)

17 unique records

Altogether 1477
Deduplication 547 records
Remaining 930

Additional information regarding meta-analysis

Test accuracy data usually follows a binomial distribution. However, there are several challenges for diagnostic accuracy meta-analyses, not least because they simultaneously analyse two outcome measures – sensitivity and specificity, which can auto-correlate with each other and of course they usually inversely correlate. They are therefore potentially affected by diagnostic trade-offs between sensitivity and specificity which in practice can generate a "threshold effect", e.g., different cut-off points for positivity across studies as well as increased study heterogeneity[1] amongst other issues. Authors have made a number of suggestions how to overcome this, including generalised linear models or hierarchical models, however these can be overly complex[2].

Separate pooling of sensitivity and specificity with fixed or random effects models have been used and found valid[1], although random-effects is preferred since high heterogeneity is common in diagnostic accuracy studies and this we followed in our study. There are a few specialised and validated STATA add-ons and commands to support diagnostic test accuracy now. For our analysis we utilised the "midas" and "metadta" commands to produce bivariate random effects meta-analysis models as well as the accompanying graphical forest plots and summary receiver operating characteristics outputs[2]. Calculation of heterogeneity is also complex and there is evidence that the classic I^2 statistic from Higgins and Thompson underestimates the expected within study variance and provides artificially high estimates. We therefore used the Zhou and Dendukuri[3] I^2 statistic to account for this mean-variance relationship between studies.

Forest plots and Summary receiver operating characteristic plots

Figure S1 – Forest plot of TST studies

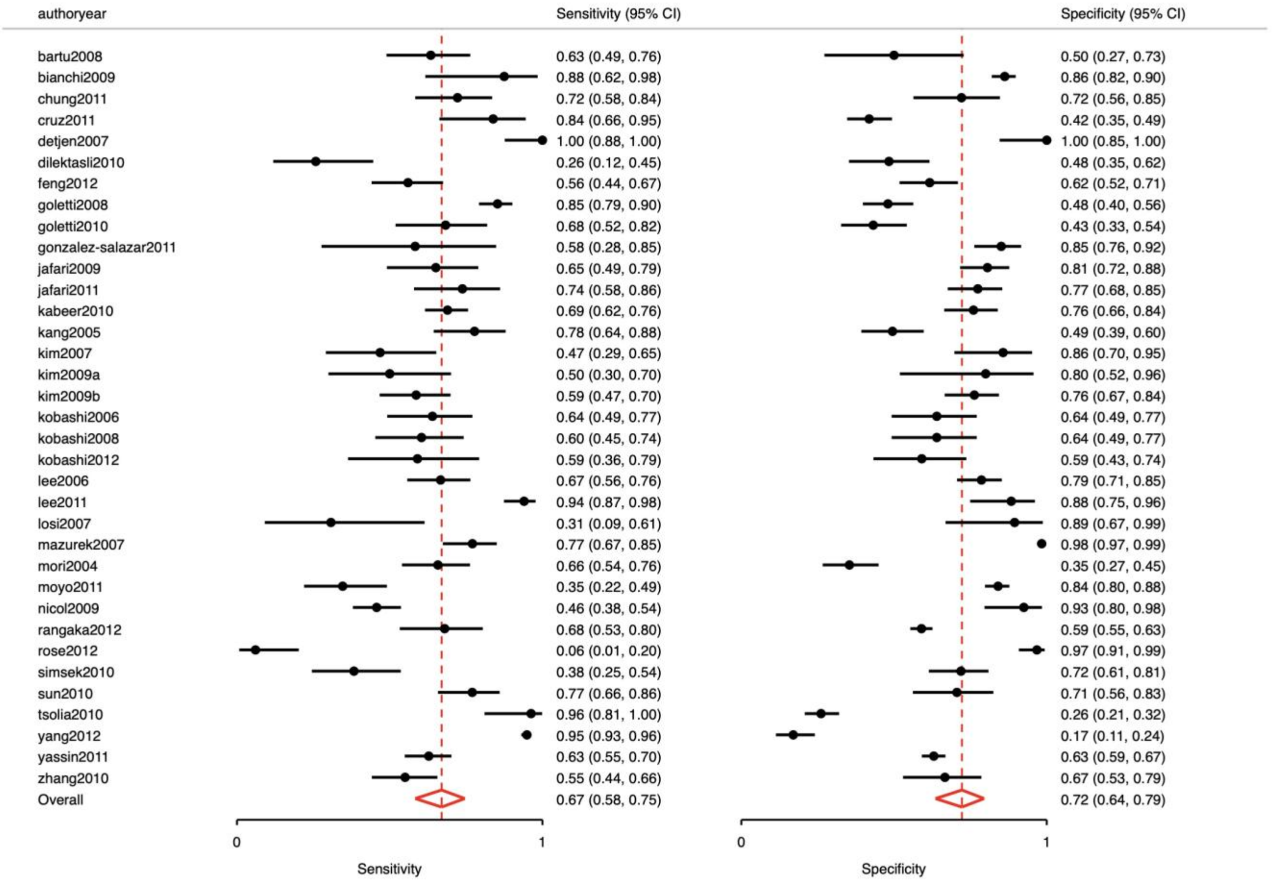

Figure S2. Summary receiver operating characteristic plot of TST studies

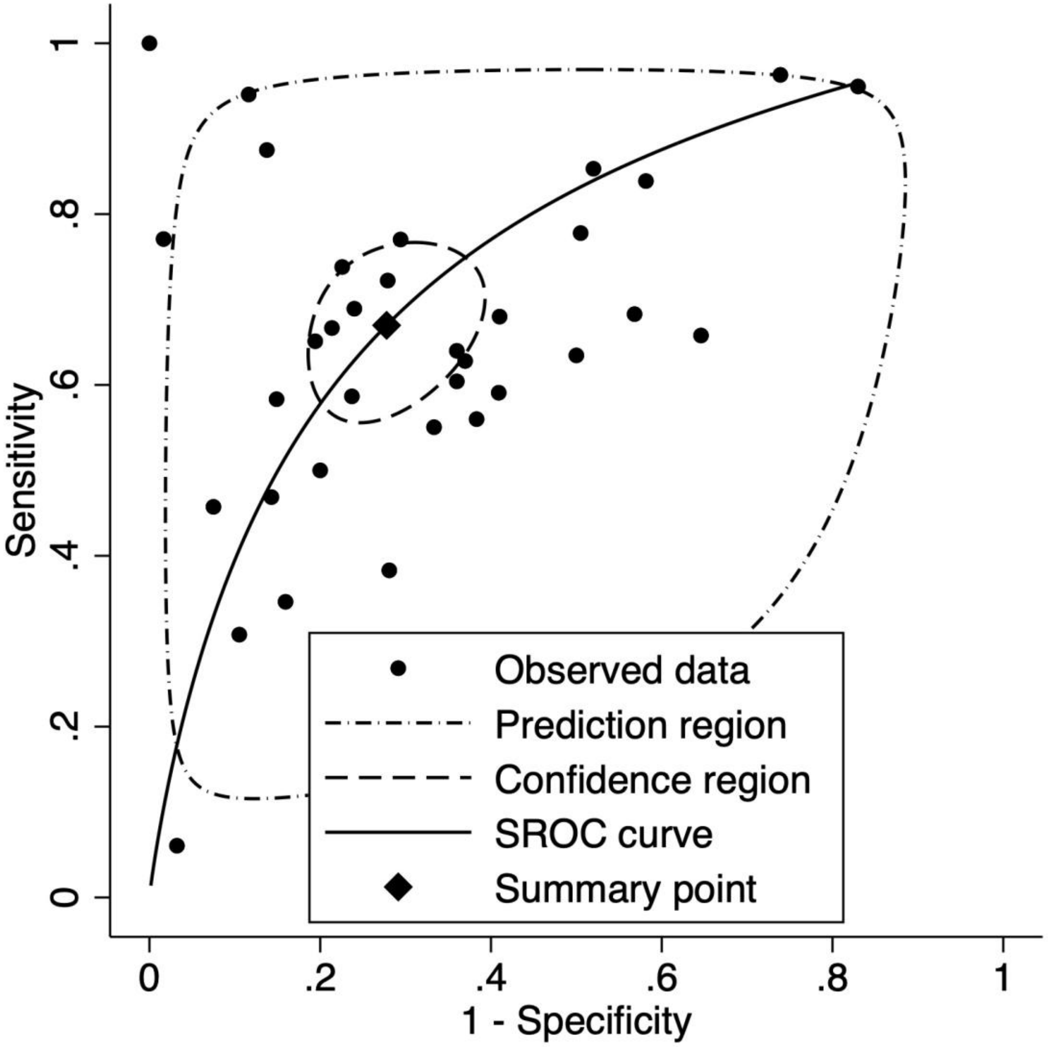

Figure S3. Forest plot of QuantiFERON studies

*NB – author names may appear more than once due to different QFT generation pooled here

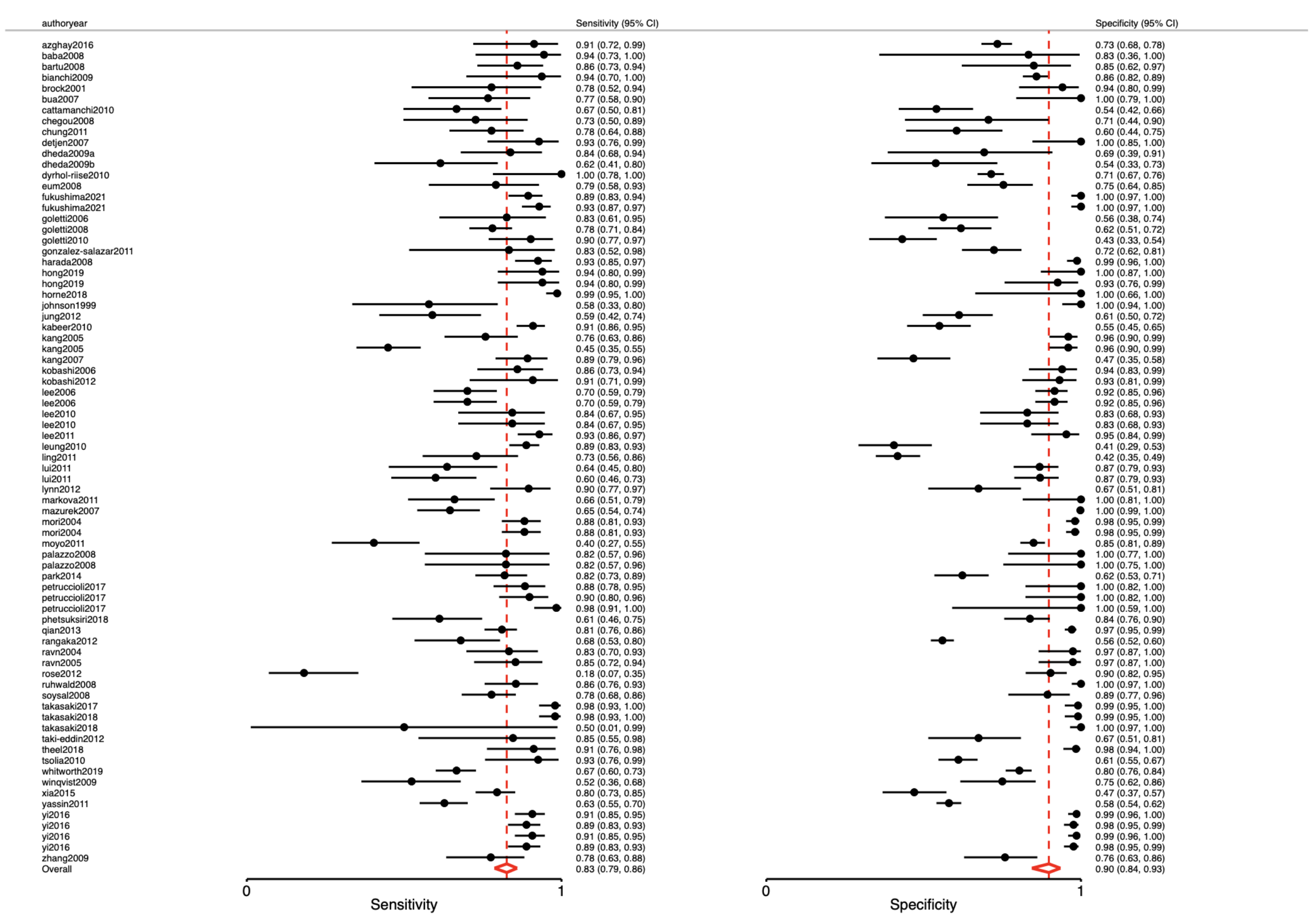

Figure S4. Summary receiver operating characteristic plot of QuantiFERON studies

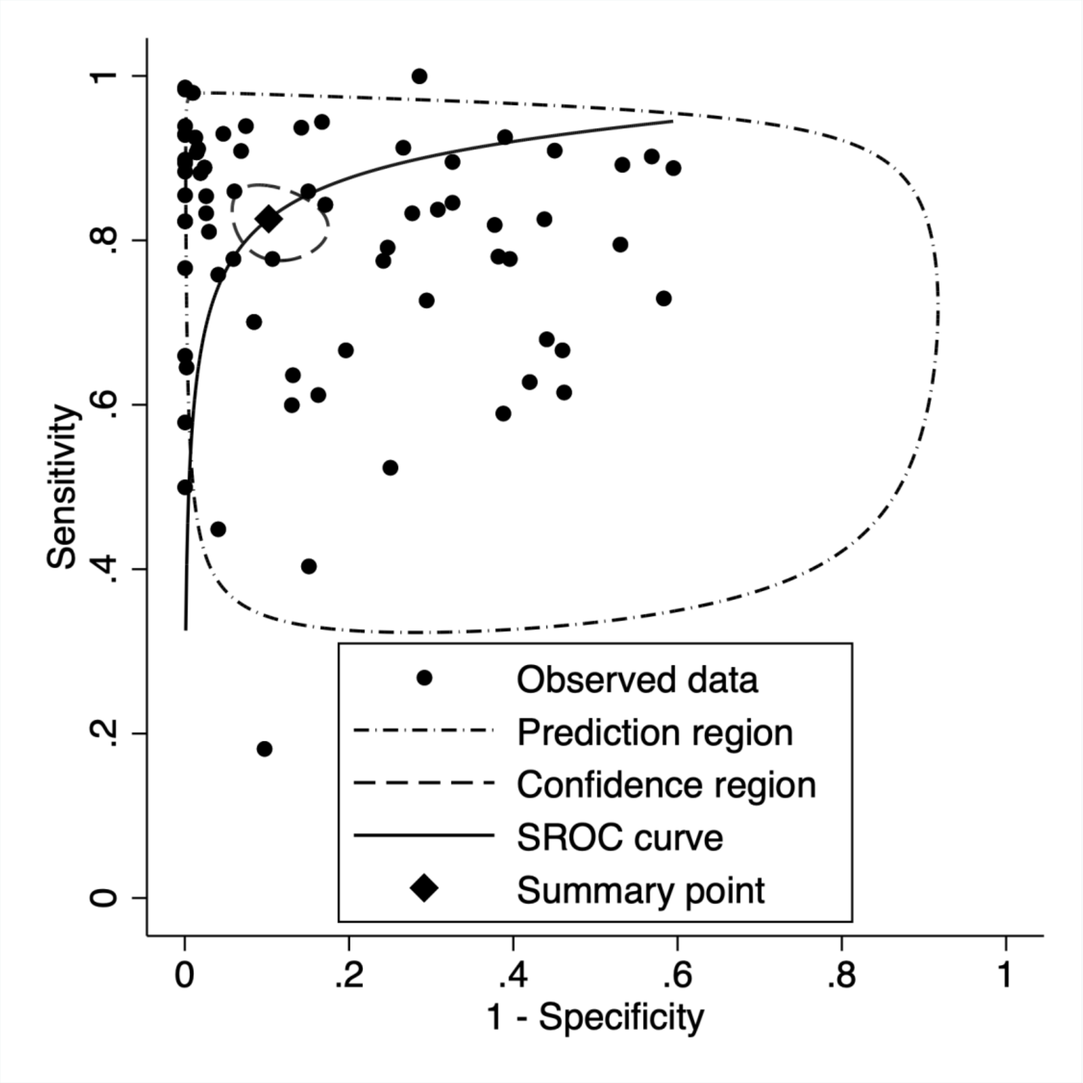
Sensitivity
1 - Specificity
0
.2
.4
.6
.8
1
Observed data
Prediction region
Confidence region
SROC curve
Summary point

Figure S5. Forest plot of T-Spot.TB studies

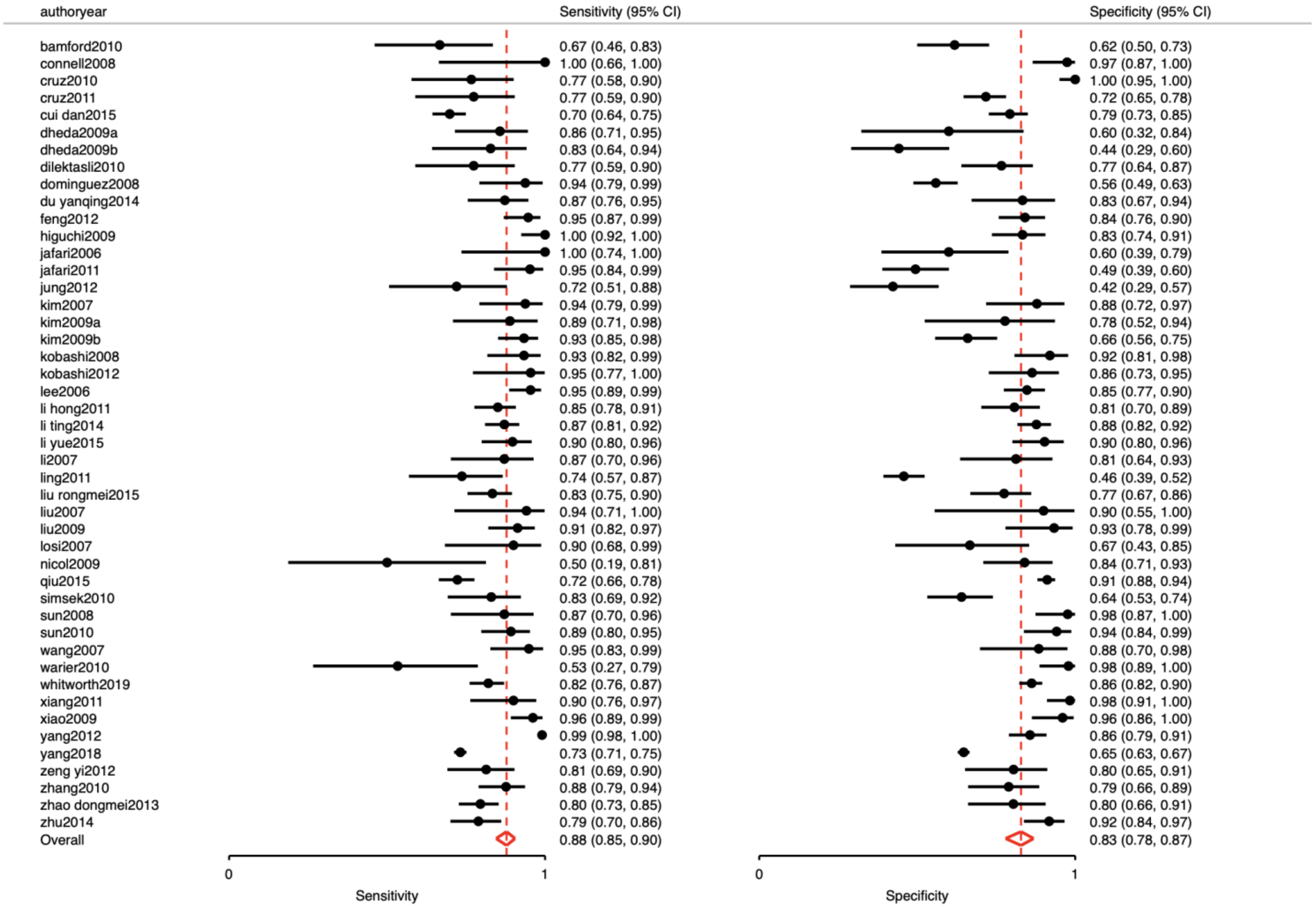

Figure S6. Summary receiver operating characteristic plot of T-Spot.TB studies

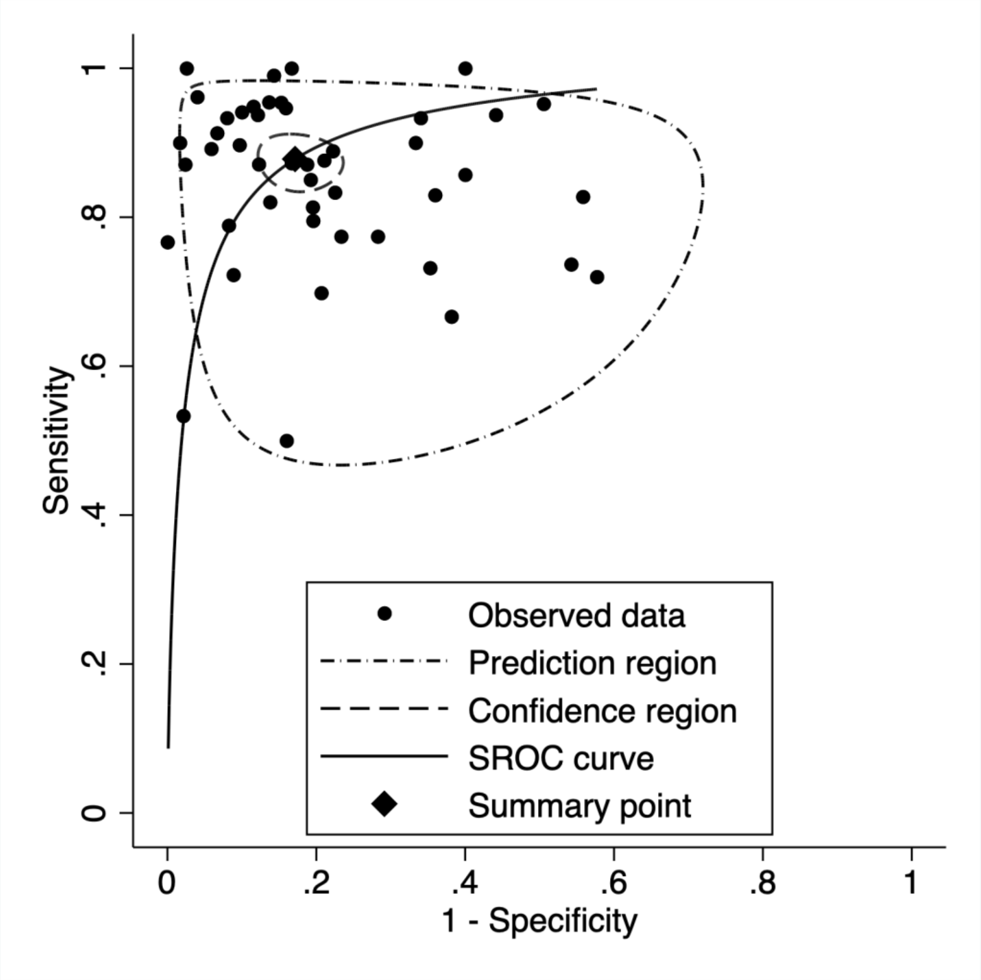

Figure S7. Forest plot of Xpert studies

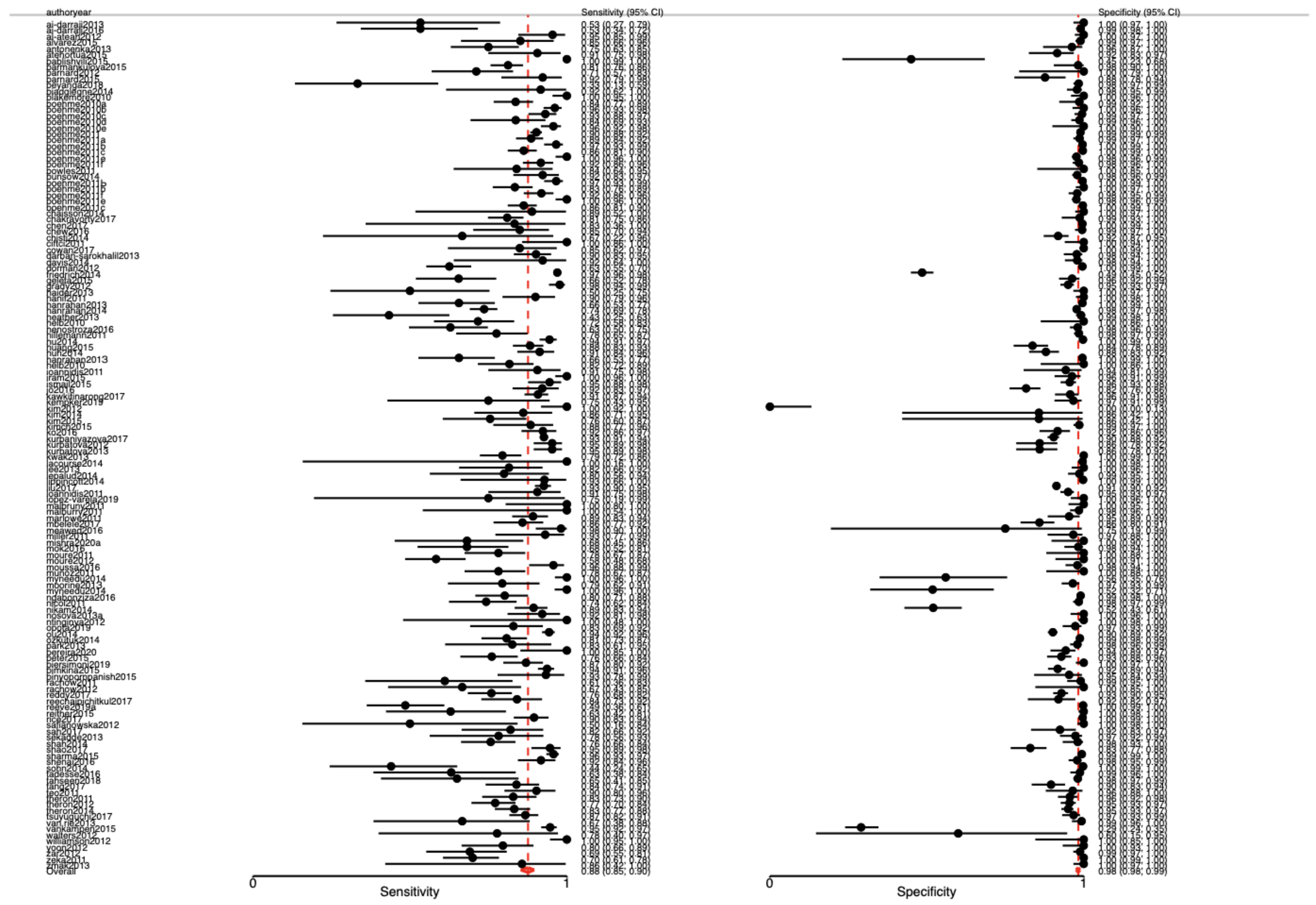

Figure S8. Summary receiver operating characteristic plot of Xpert studies

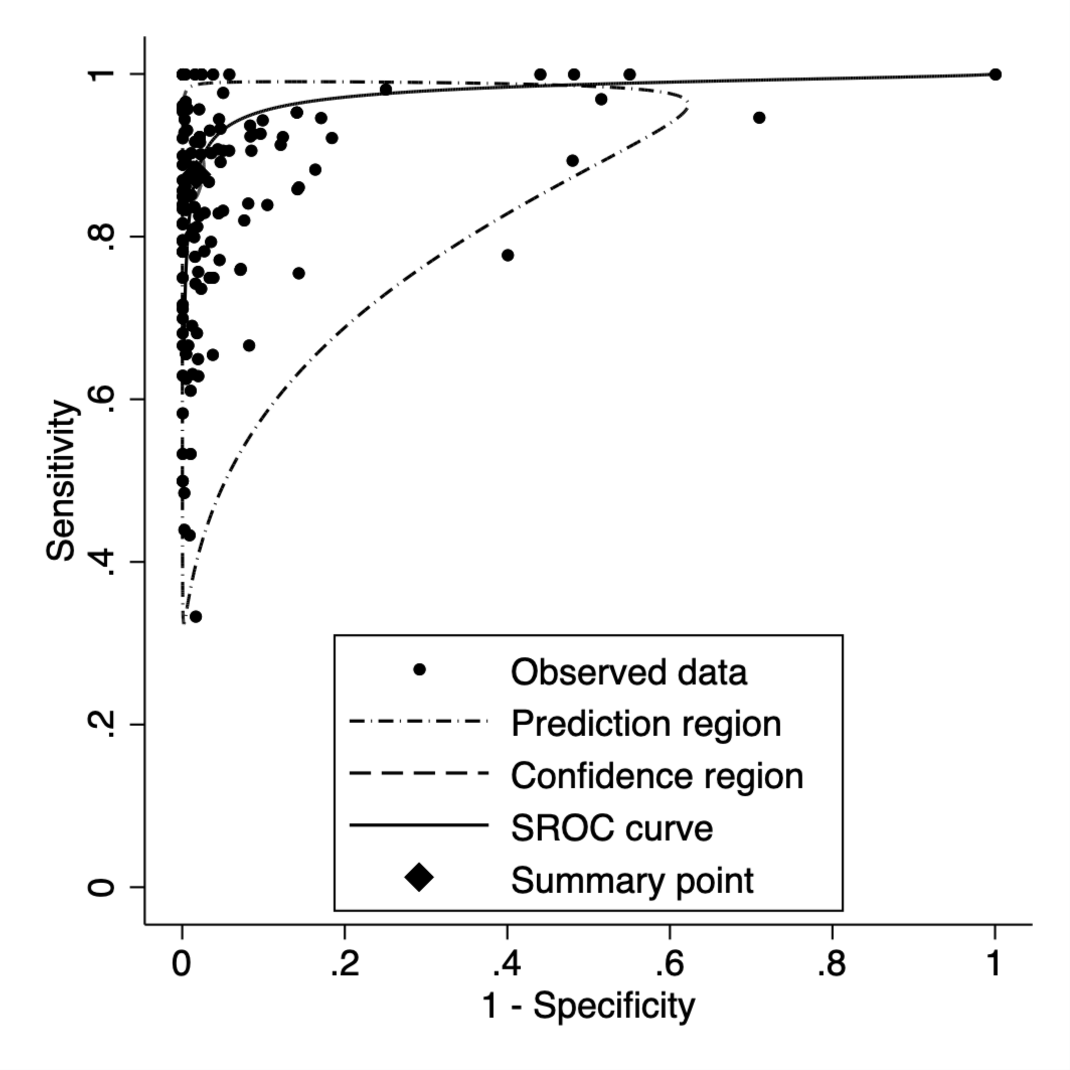

Figure S9. Forest plot of Ultra studies

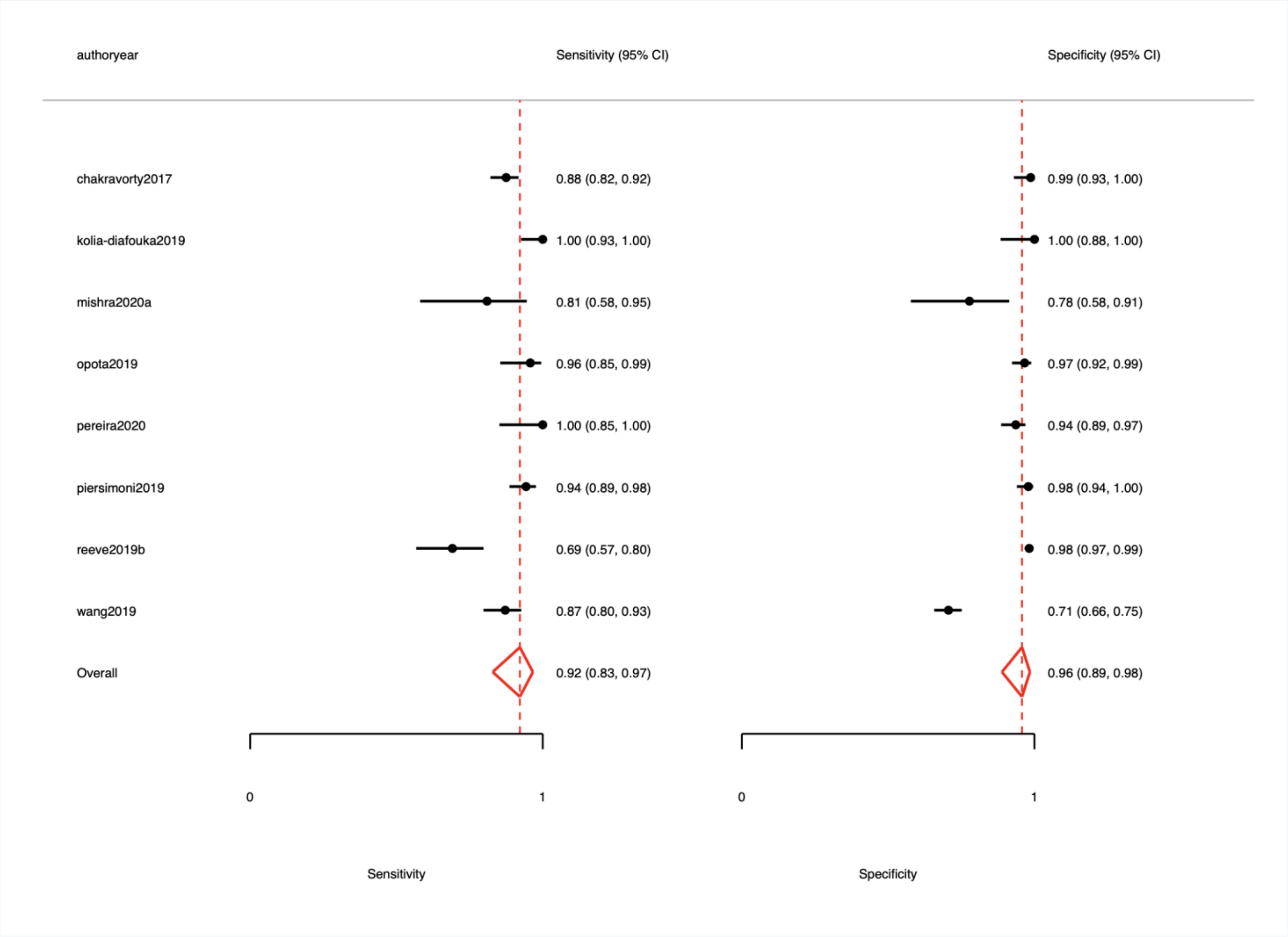

Figure S10. Summary receiver operating characteristic plot of Ultra studies

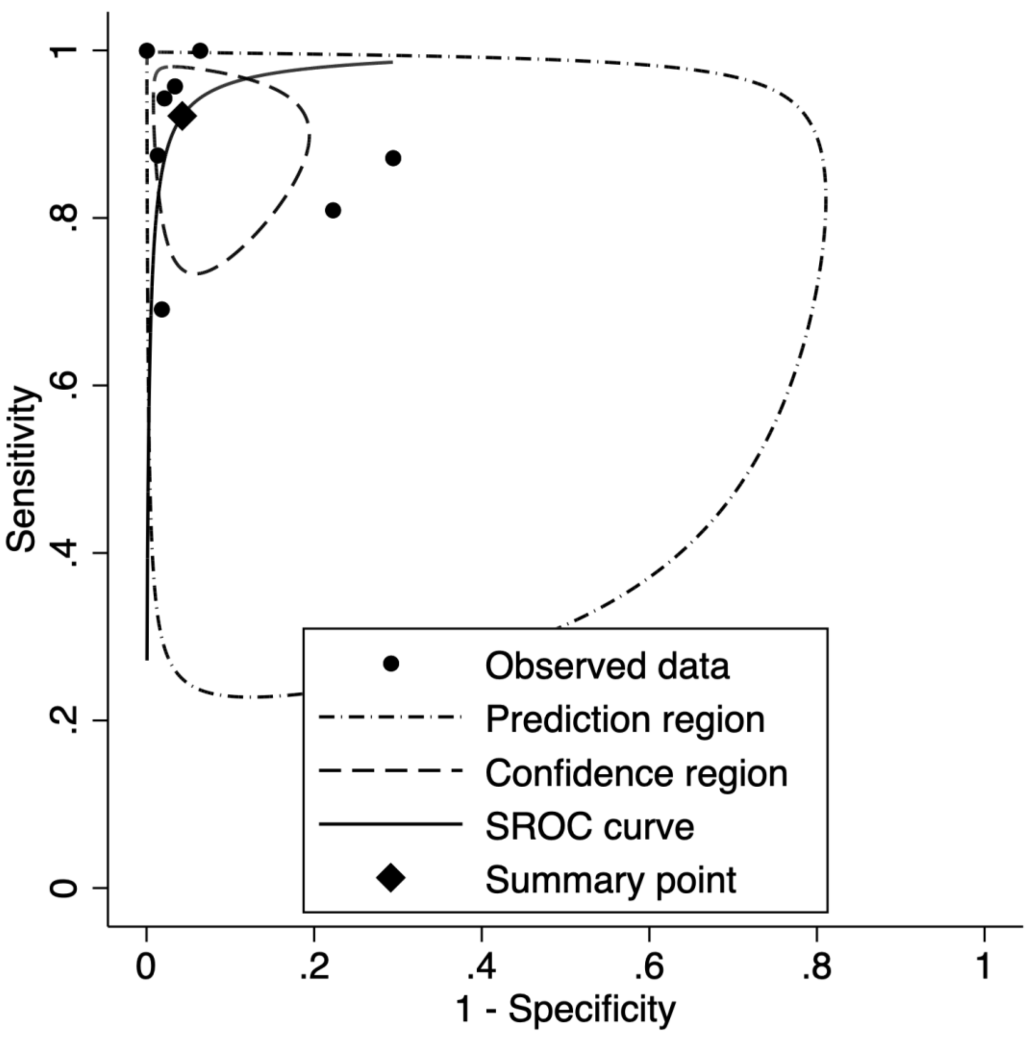

Figure S11. Forest plot of LAMP studies

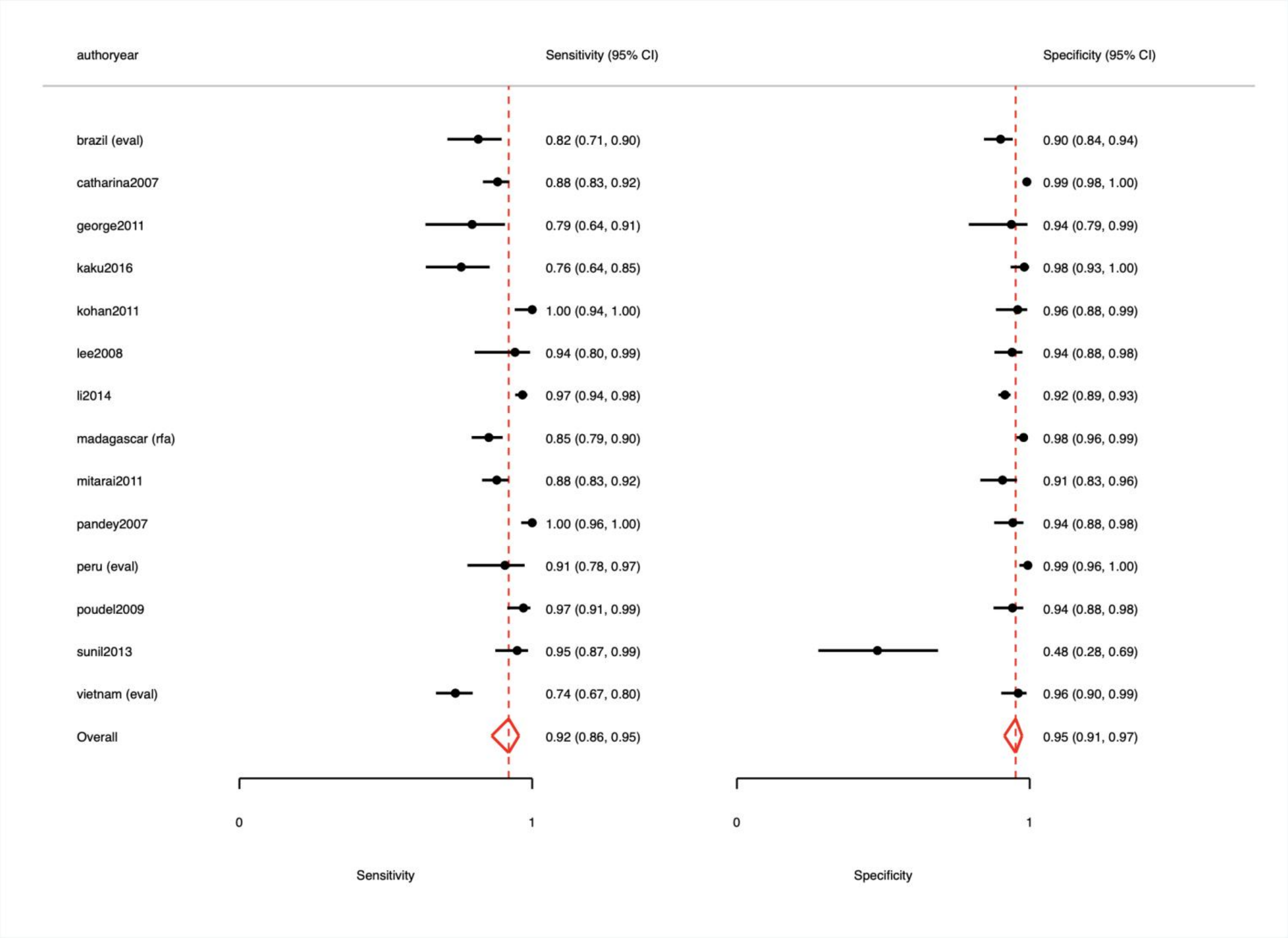

Figure S12. Summary receiver operating characteristic plot of LAMP studies

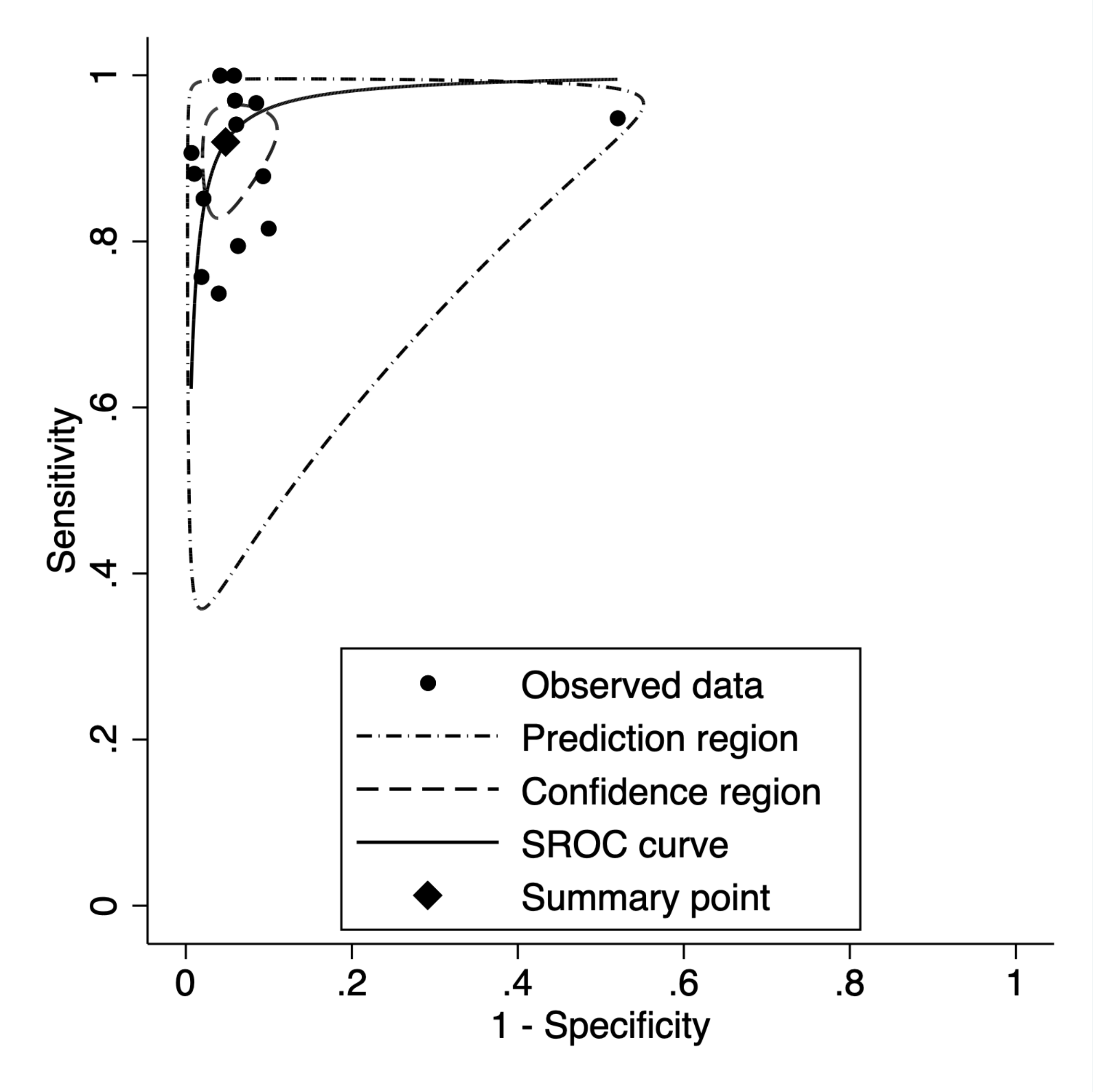

Figure S13. Forest plot of SAT studies

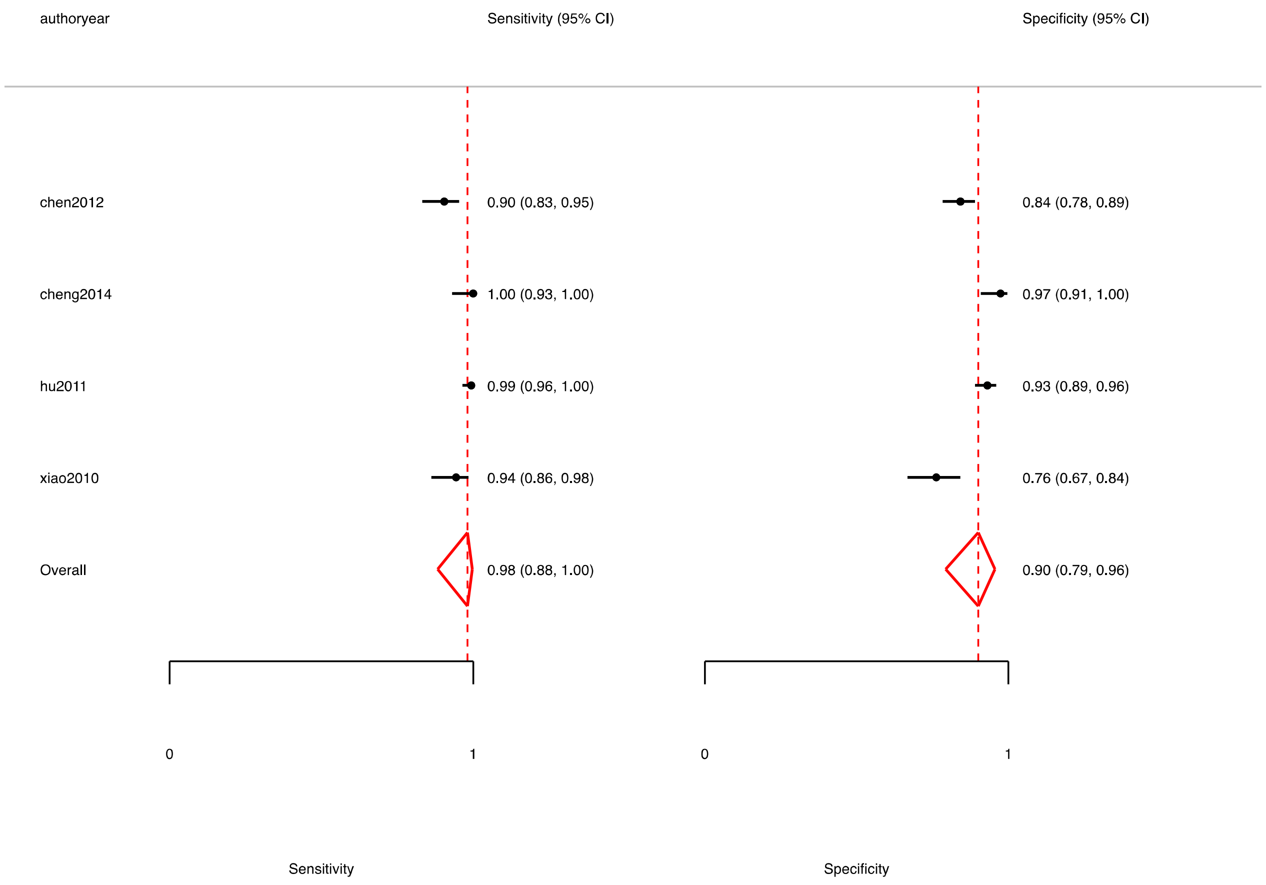

Figure S14. Summary receiver operating characteristic plot of SAT studies

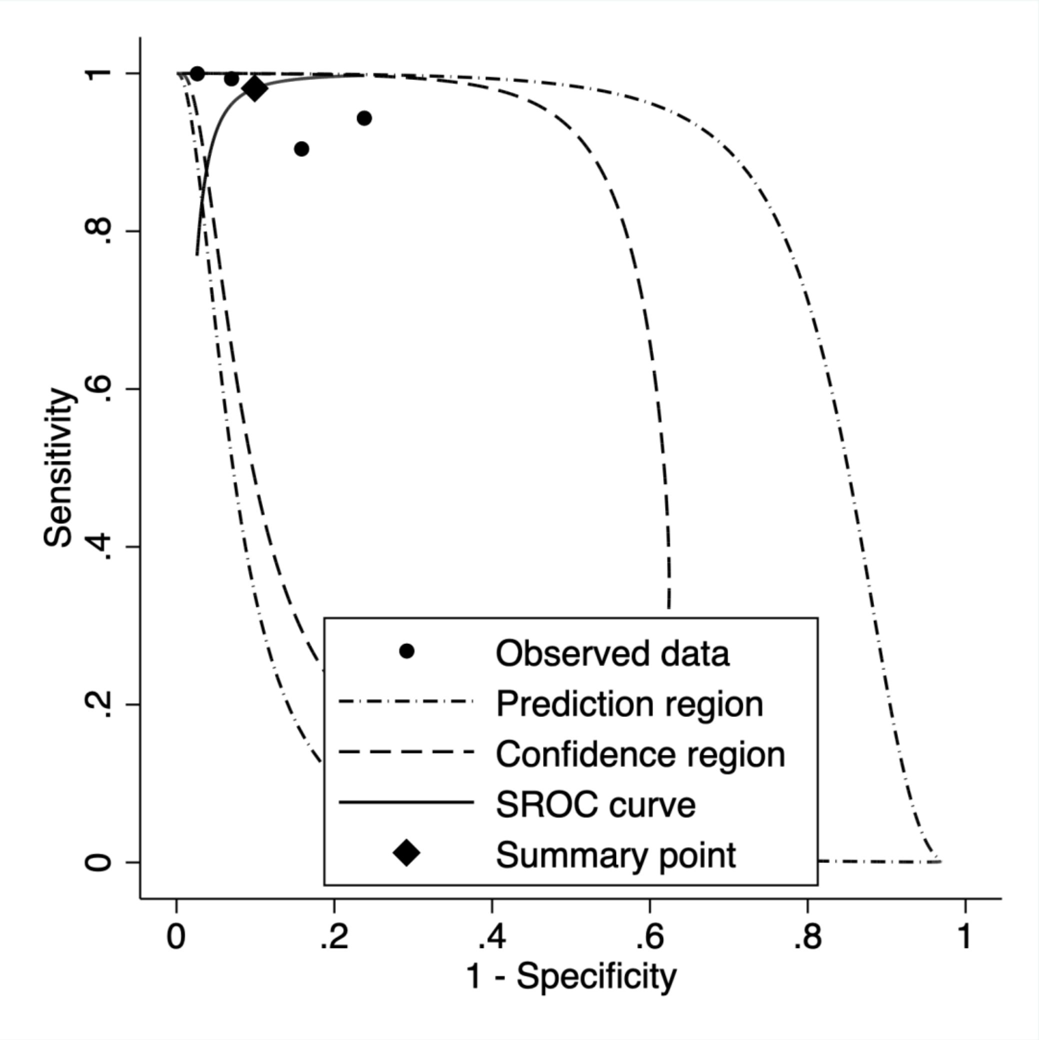

Figure S15. Forest plot of 2week cough studies

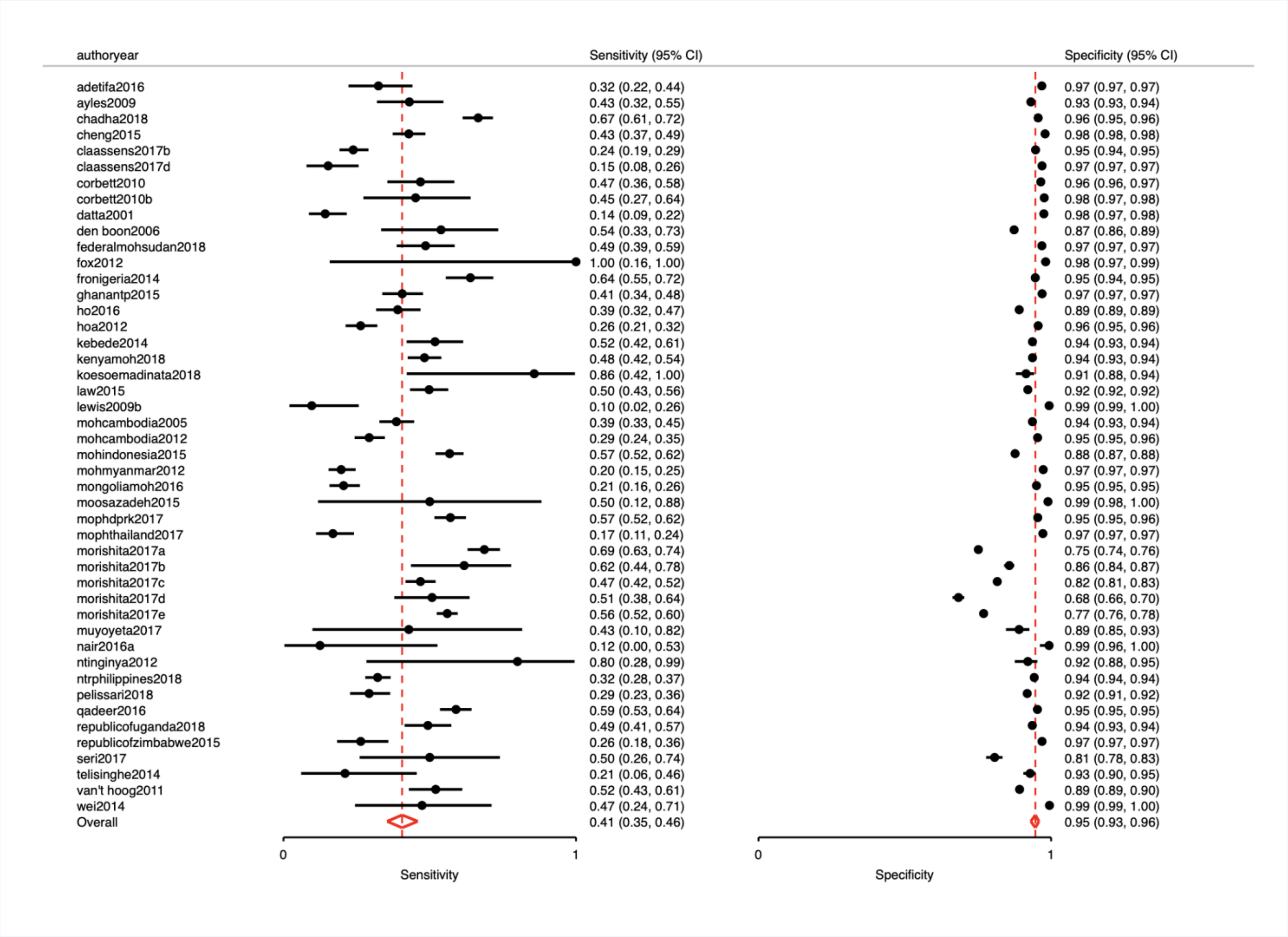

Figure S16. Summary receiver operating characteristic plot of 2week cough studies

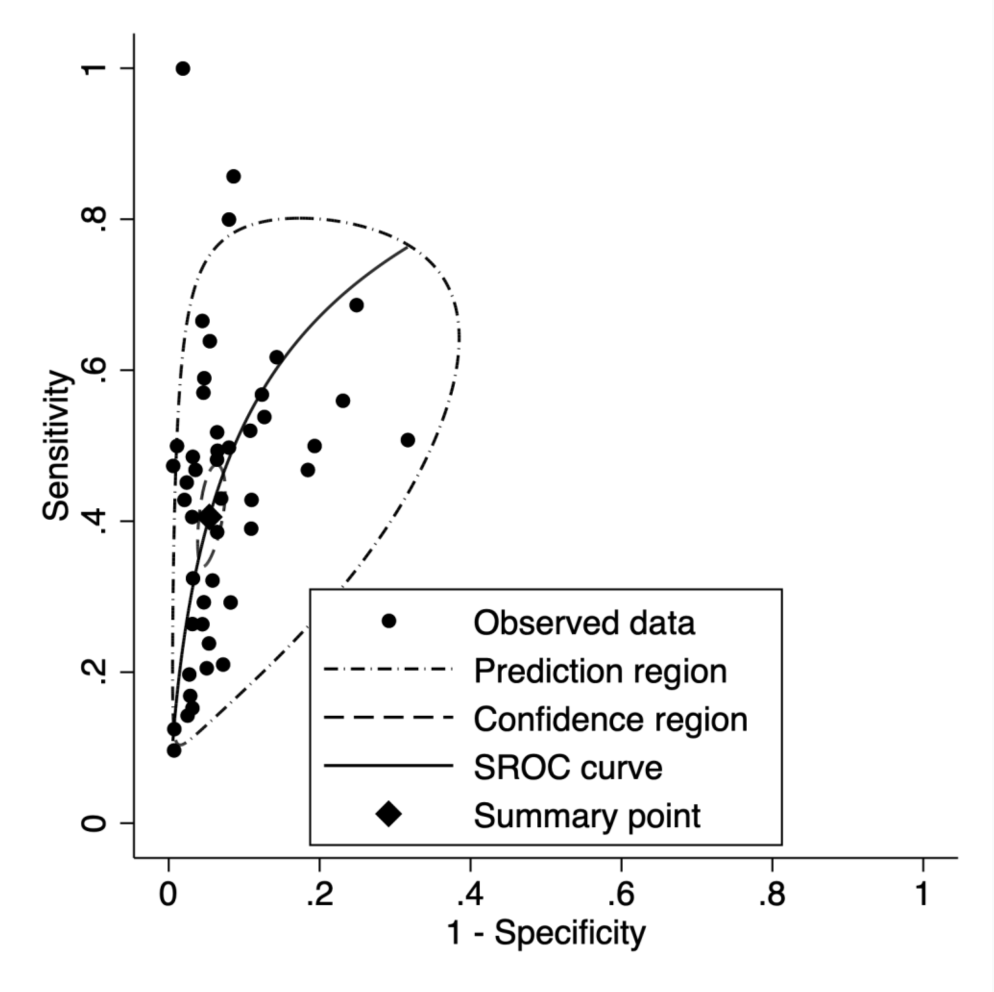

Figure S17. Forest plot of any cough studies

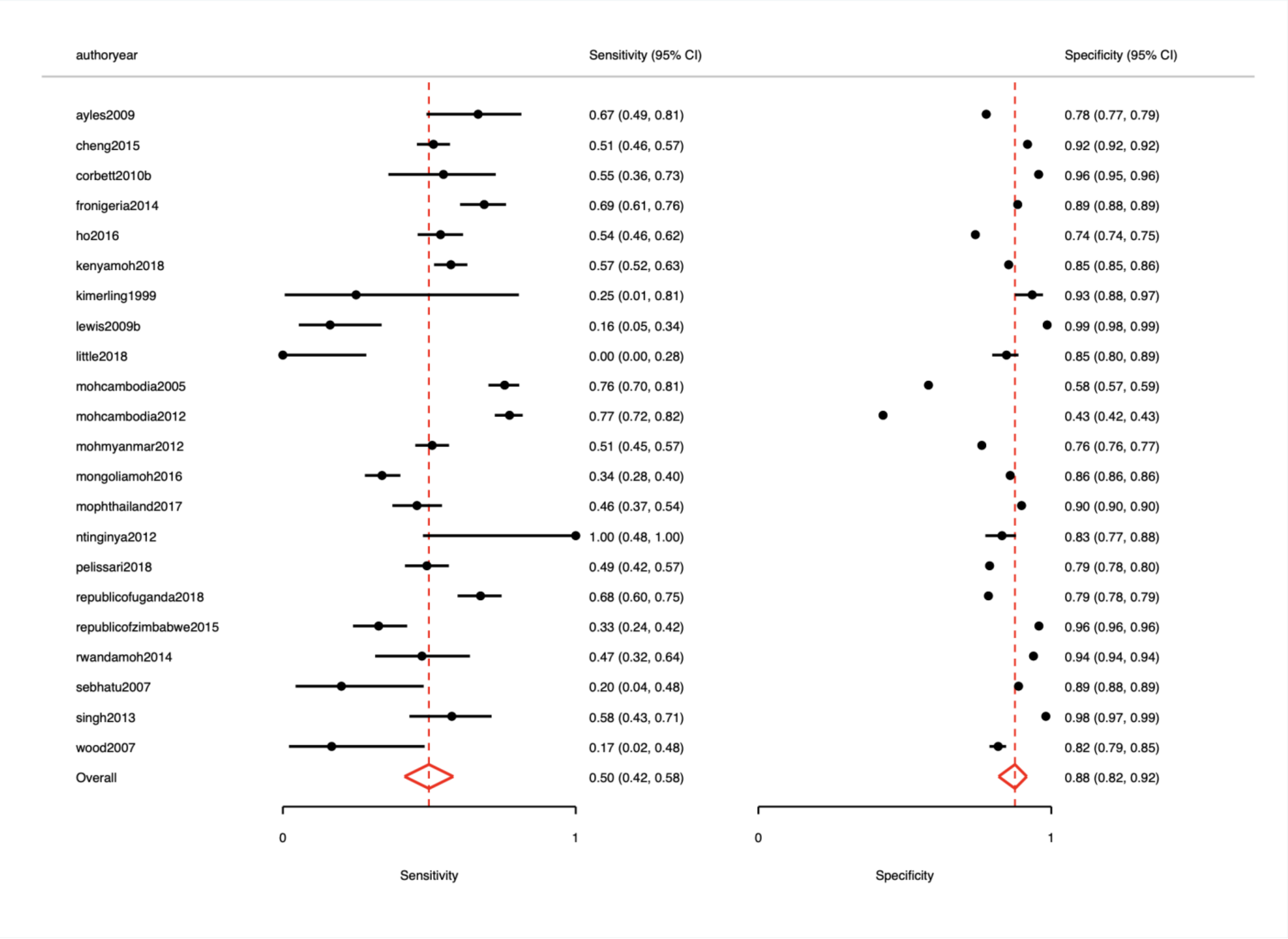

Figure S18. Summary receiver operating characteristic plot of any cough studies

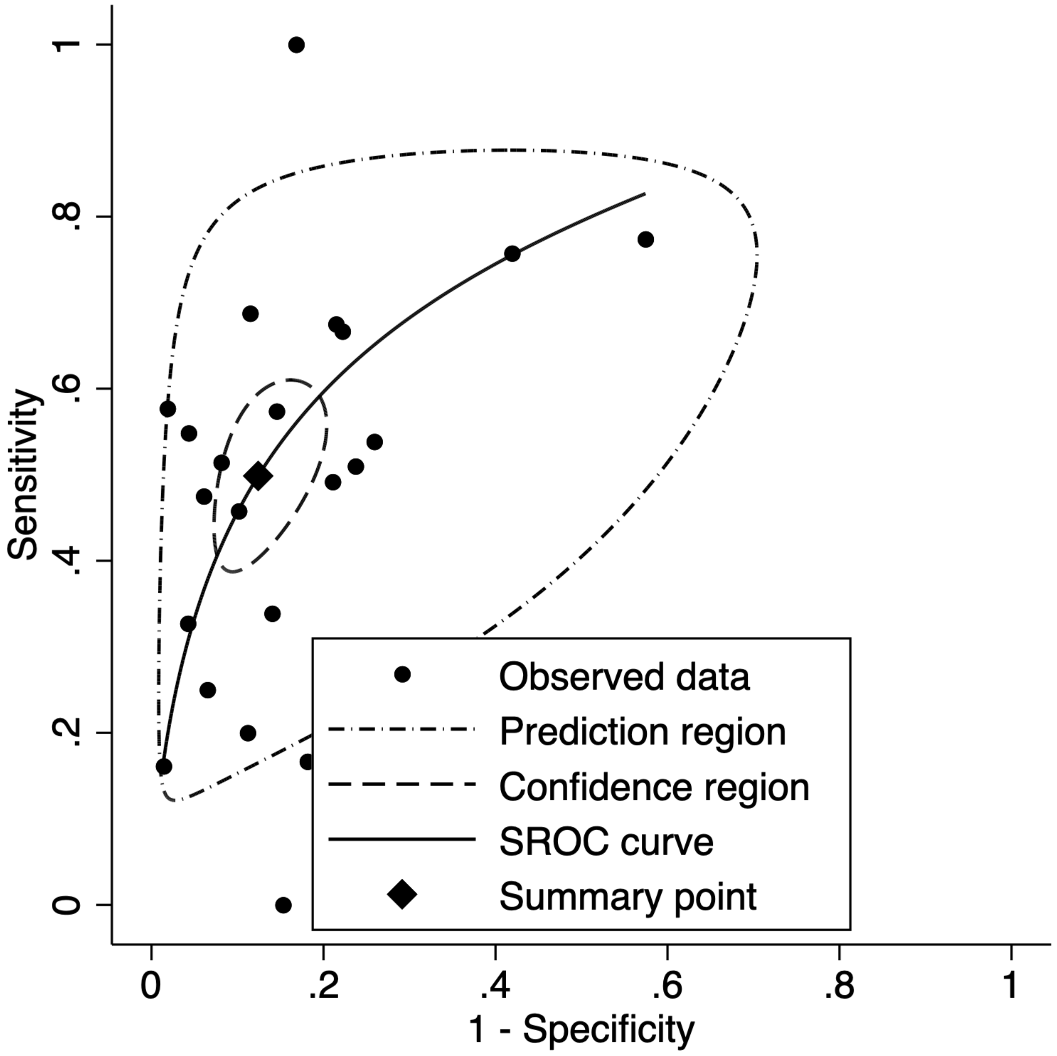

Figure S18. Forest plot of any CXR abnormality studies

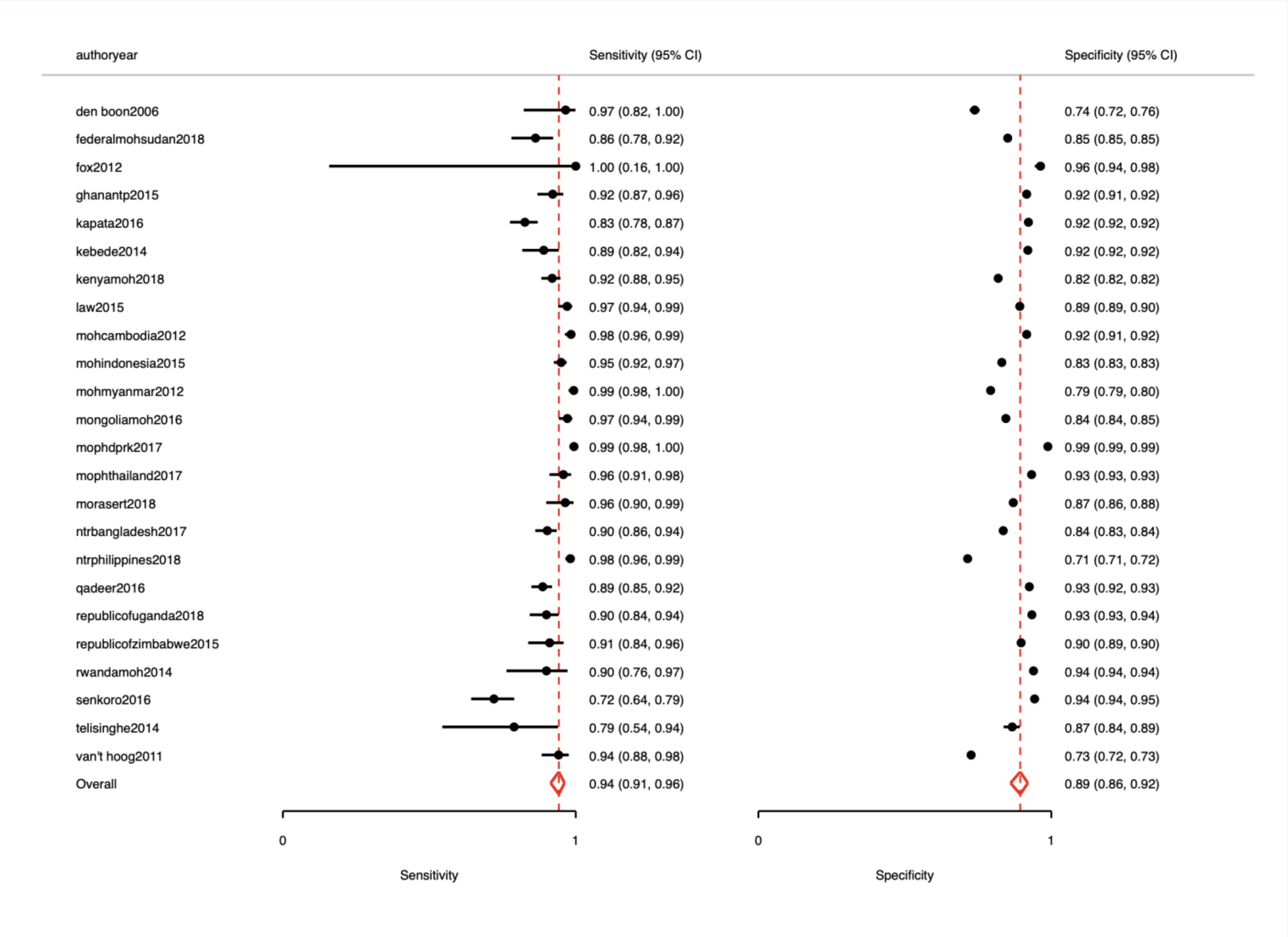

Figure S20. Summary receiver operating characteristic plot of any CXR abnormality studies

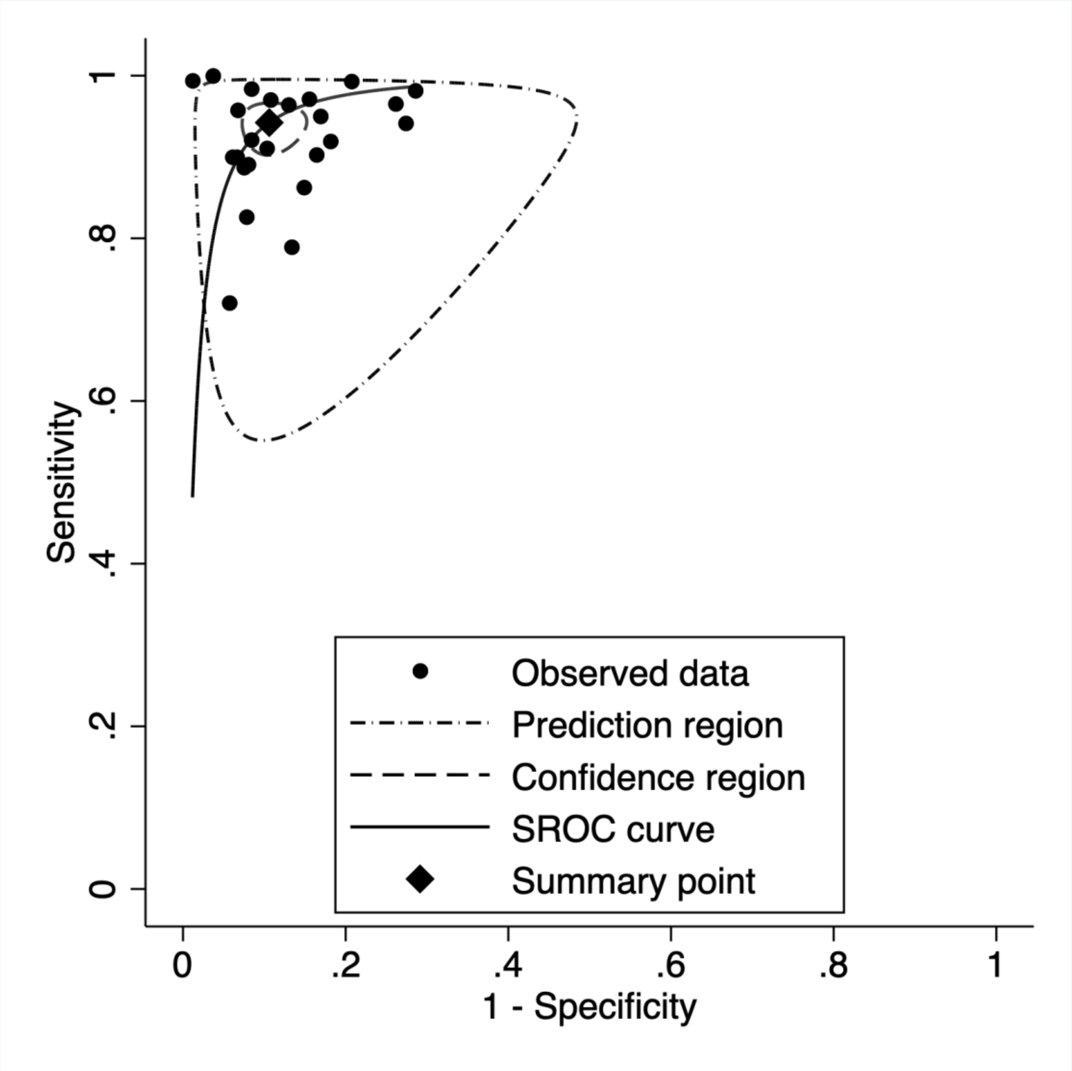

Figure S21. Forest plot of CXR TB abnormality studies

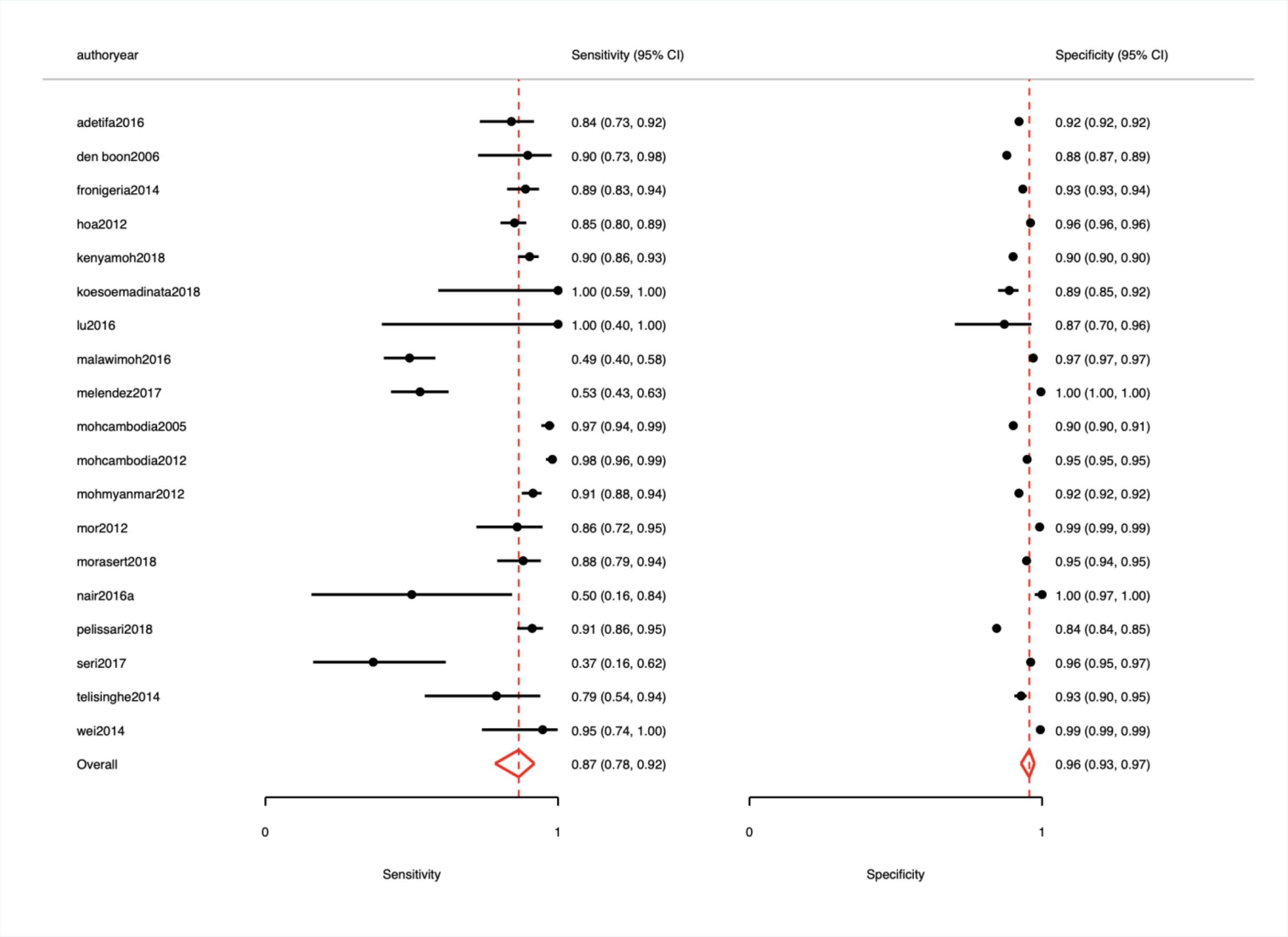

Figure S22. Summary receiver operating characteristic plot of CXR TB abnormality studies

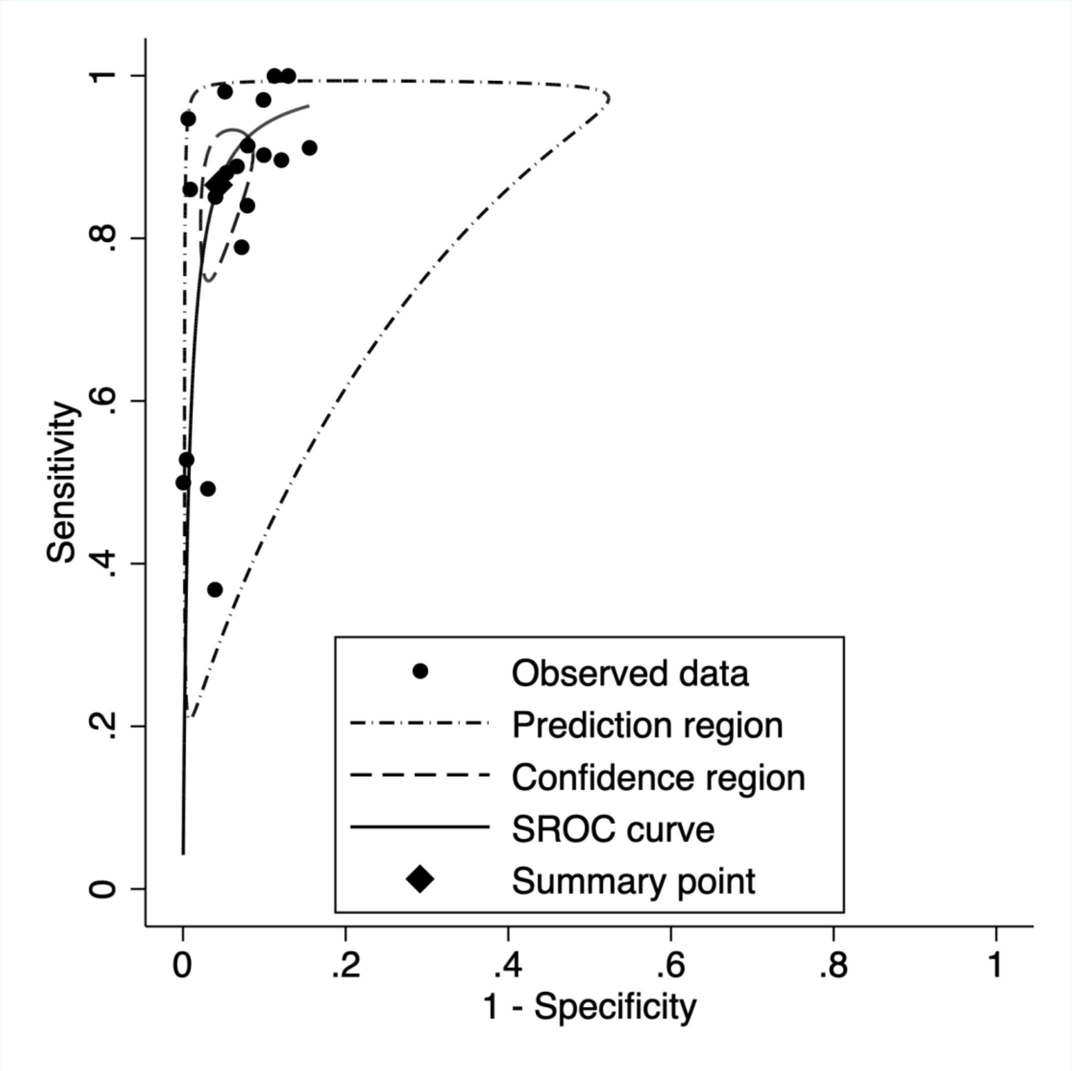

Figure S23. Forest plot of parallel symptom (cough)/ CXR studies

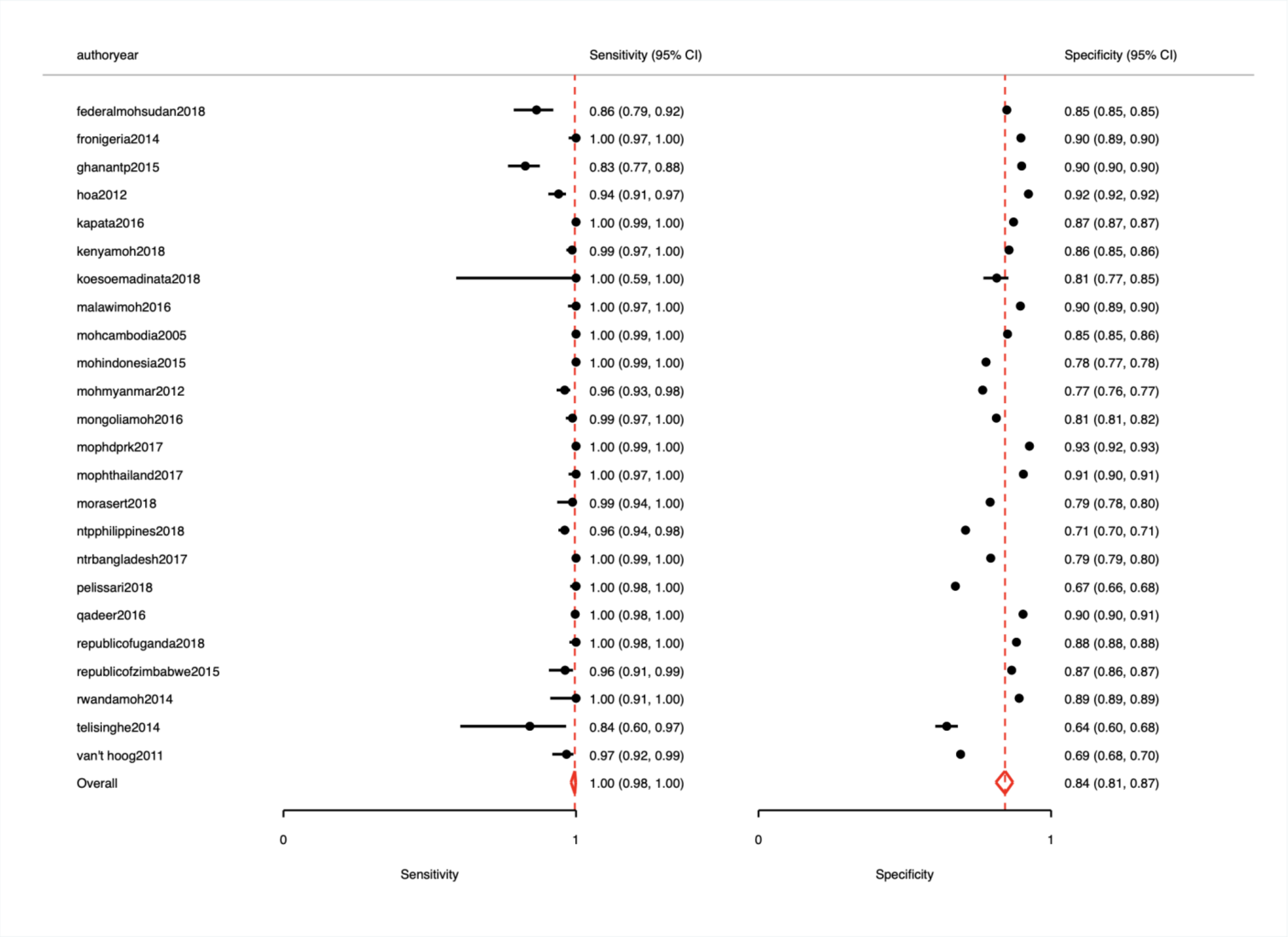

Figure S24. Summary receiver operating characteristic plot of parallel symptom (cough)/ CXR studies

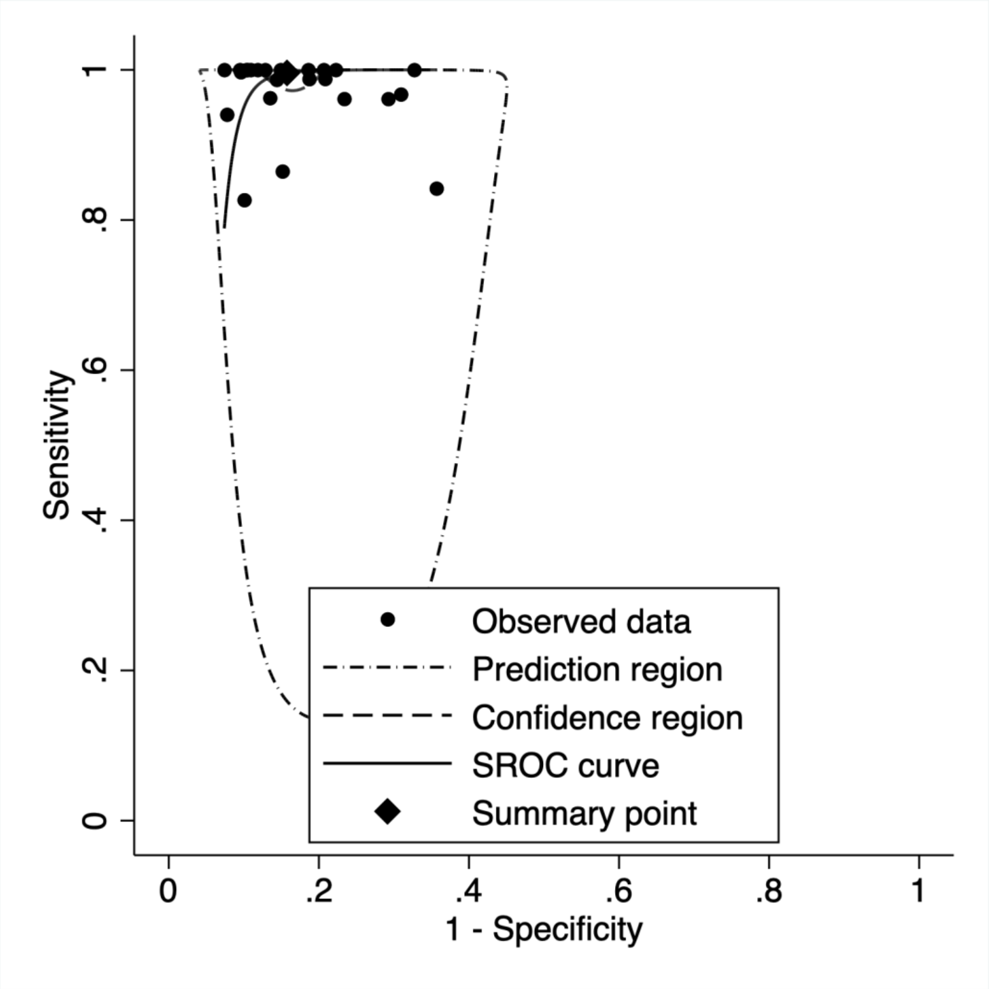

Figure S25. Forest plot of any TB symptom studies

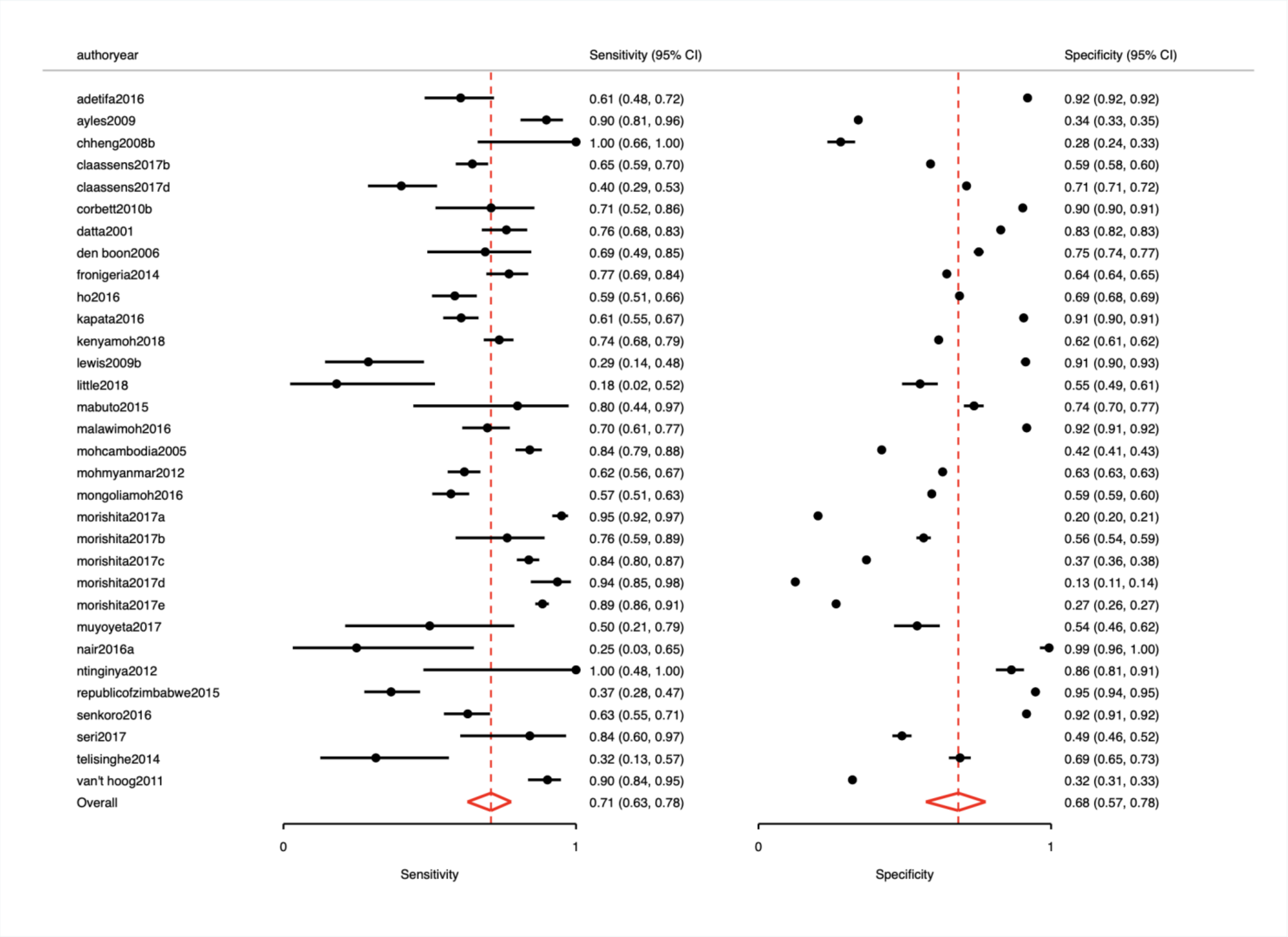

Figure S26. Summary receiver operating characteristic plot of any TB symptom studies

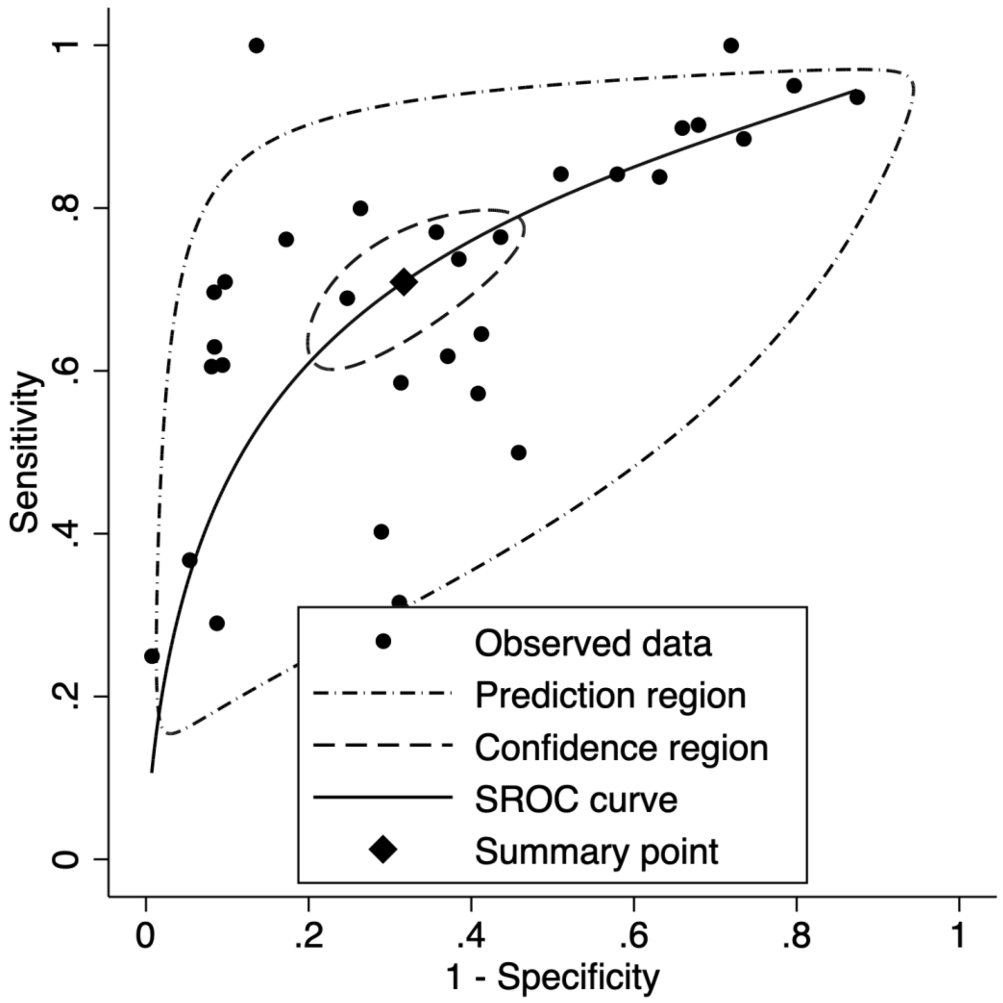

Funnel plots by screening test

Figure S27. Publication bias - TST

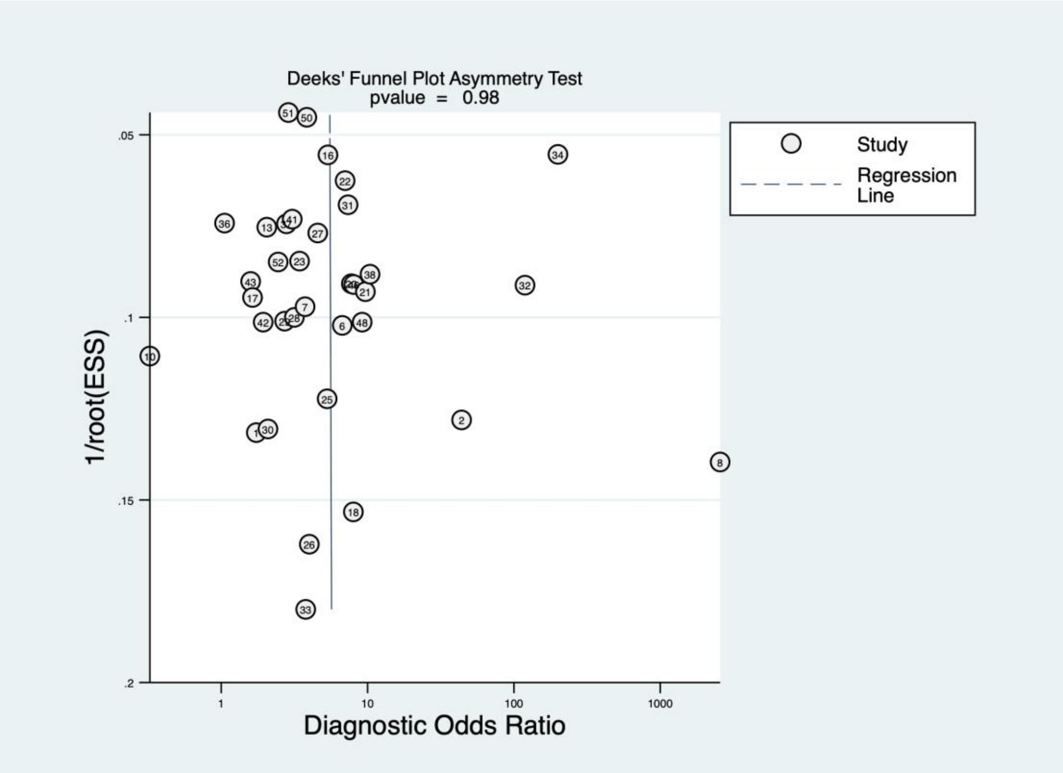

Figure S28. Publication bias - QuantiFERON

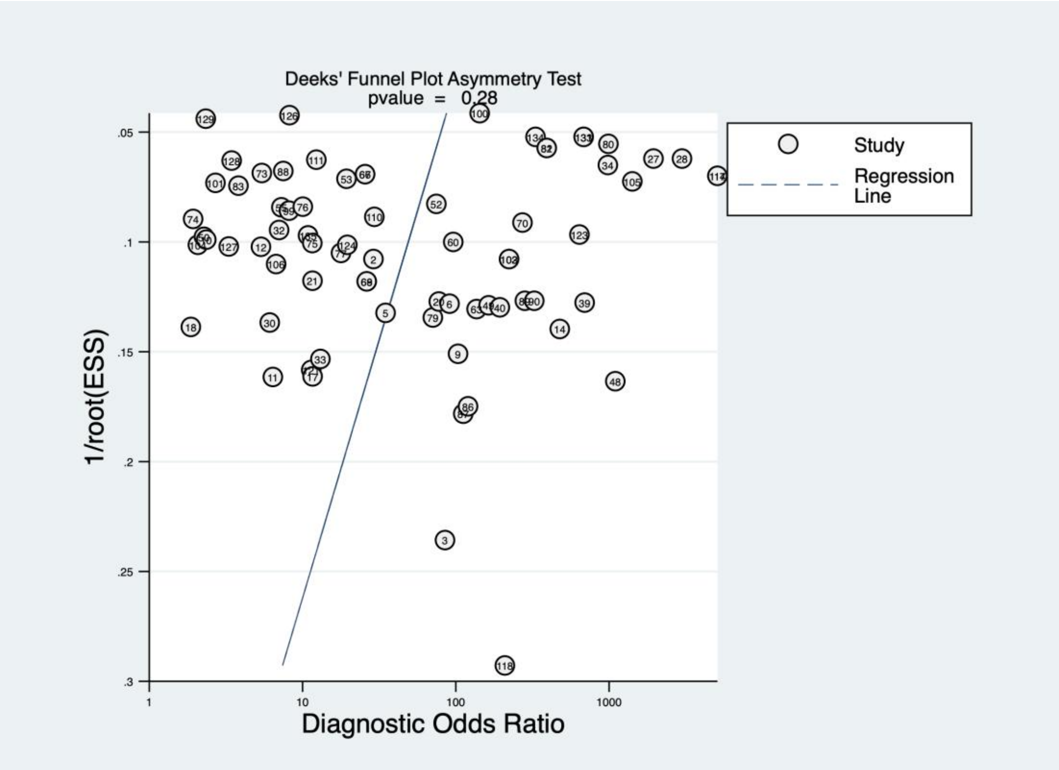

Figure S29. Publication bias – T-Spot.TB

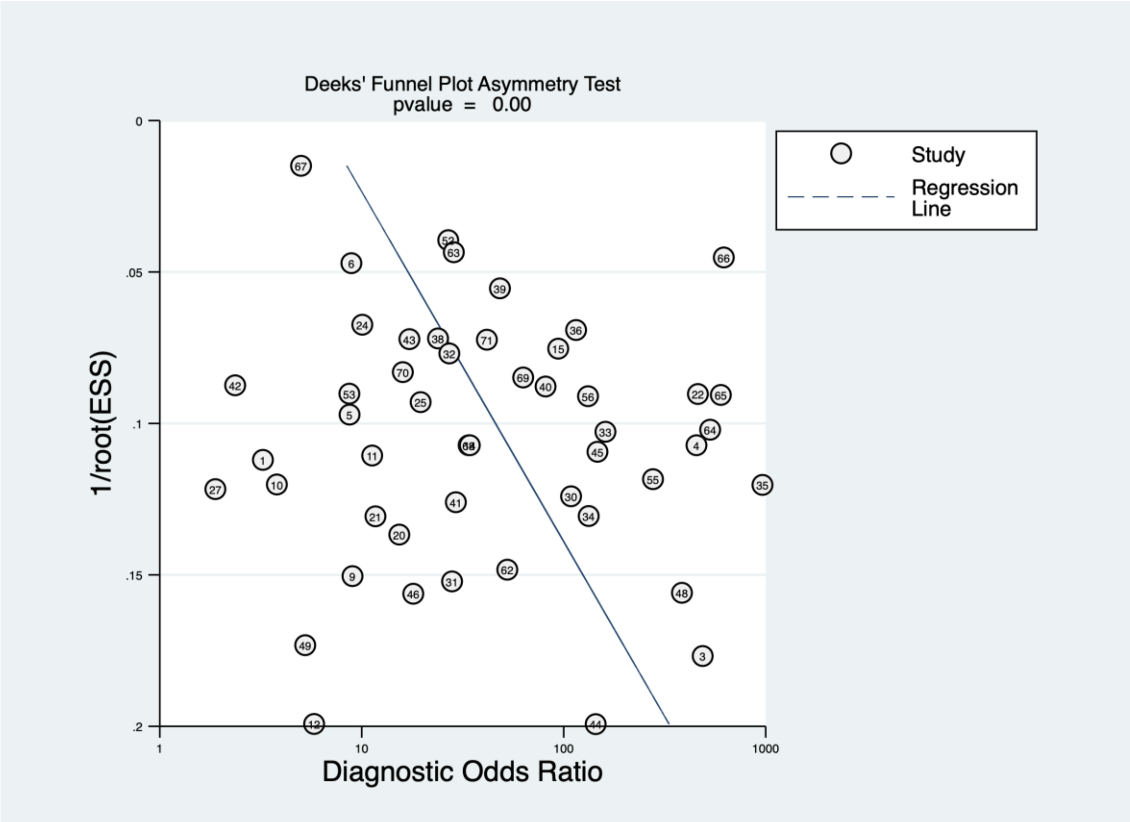

Figure S30. Publication bias - Xpert

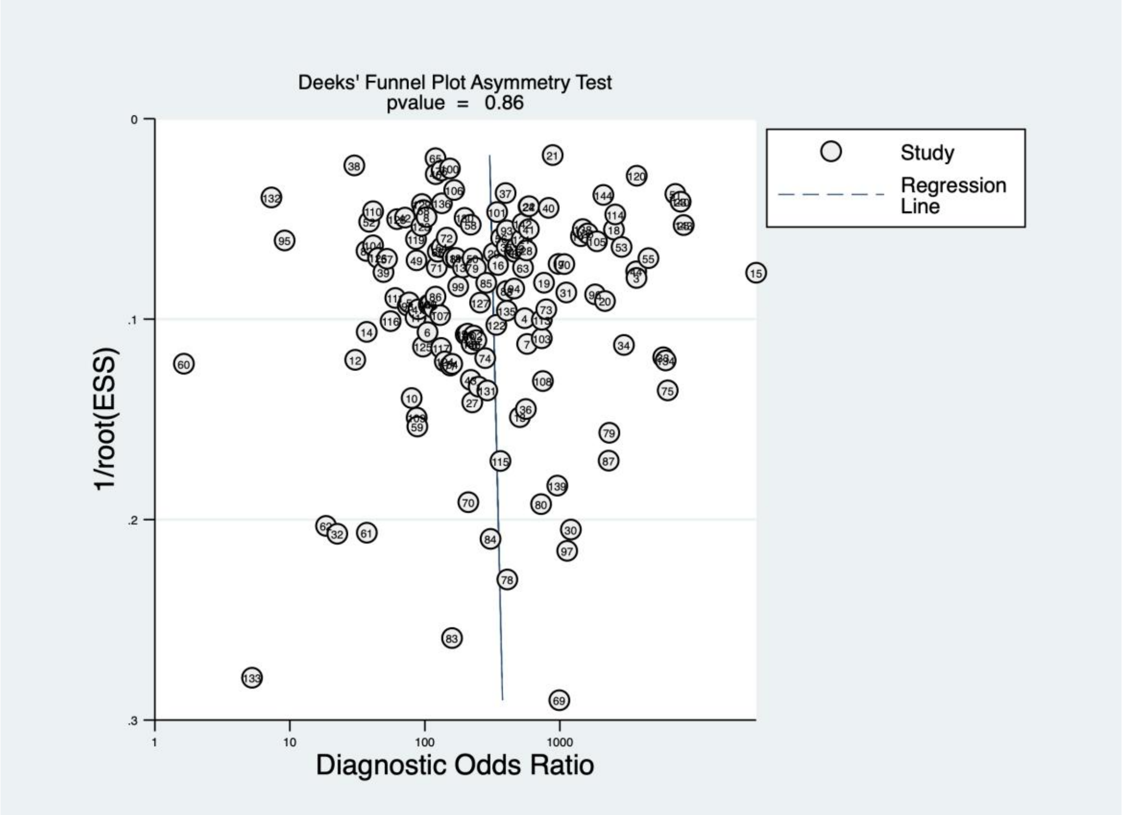

Figure S31. Publication bias - Ultra

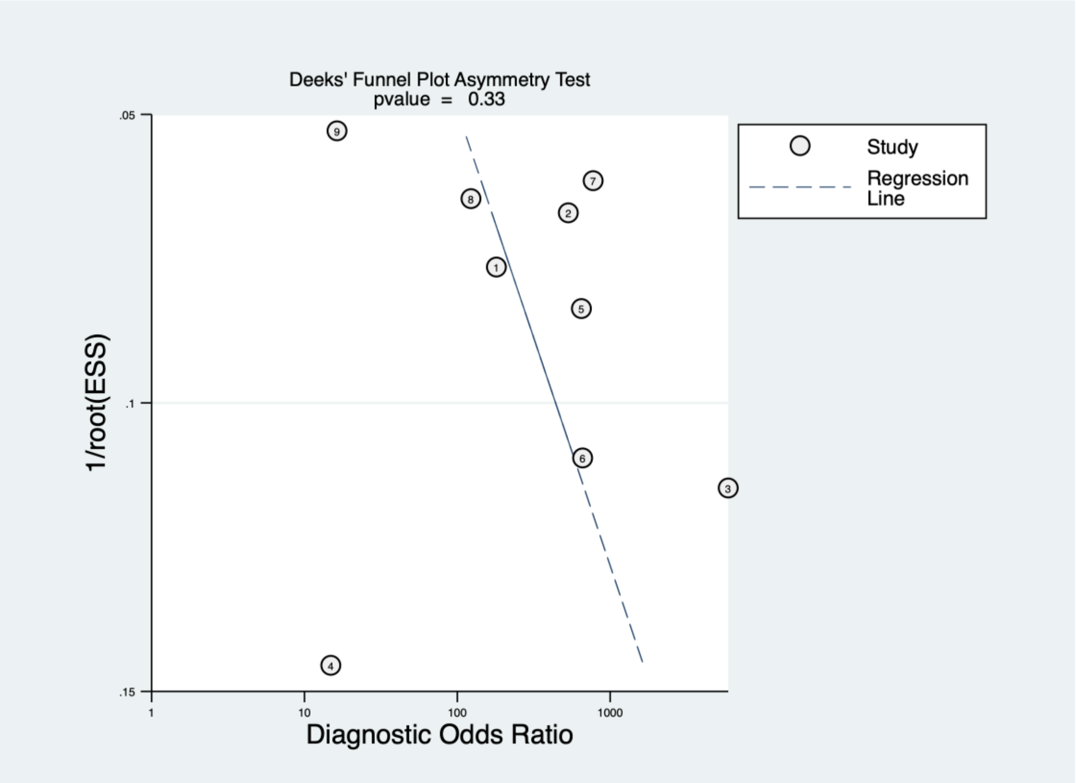

Figure S32. Publication bias - LAMP

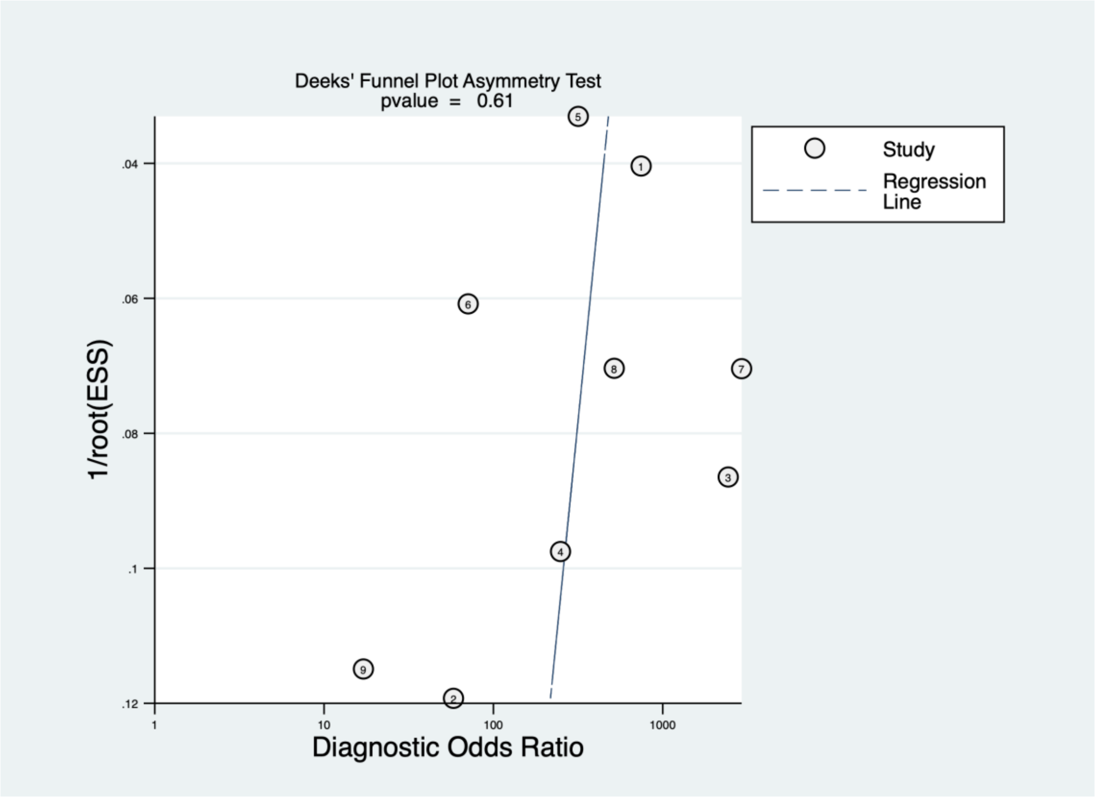

Figure S33. Publication bias - SAT

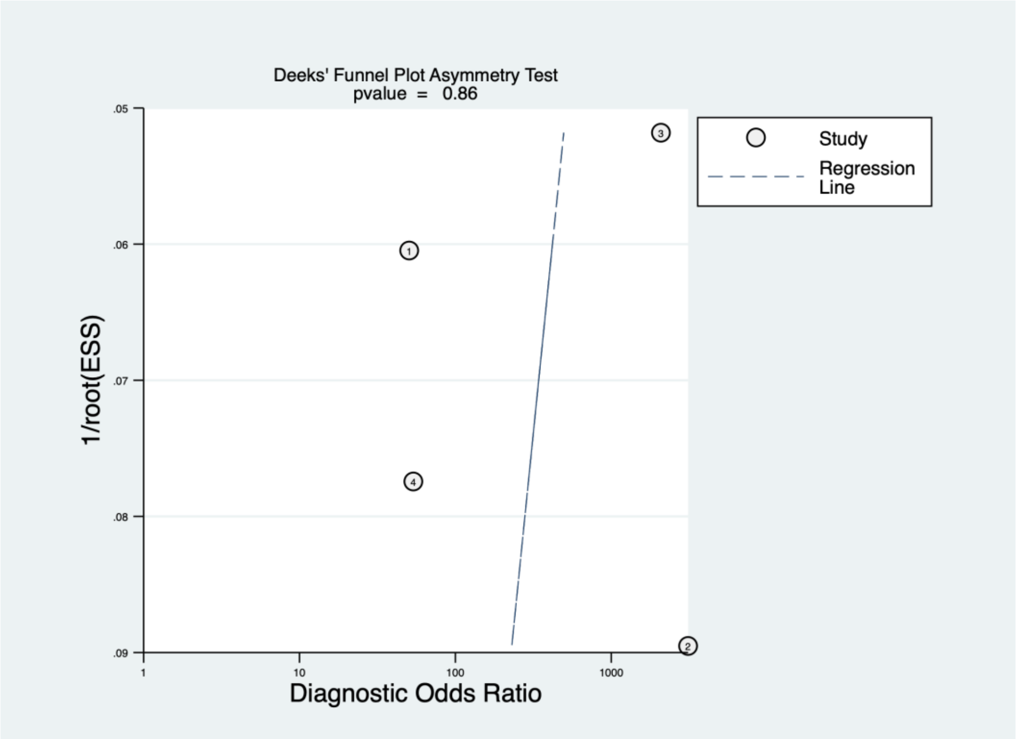

Figure S34. Publication bias – 2wk cough

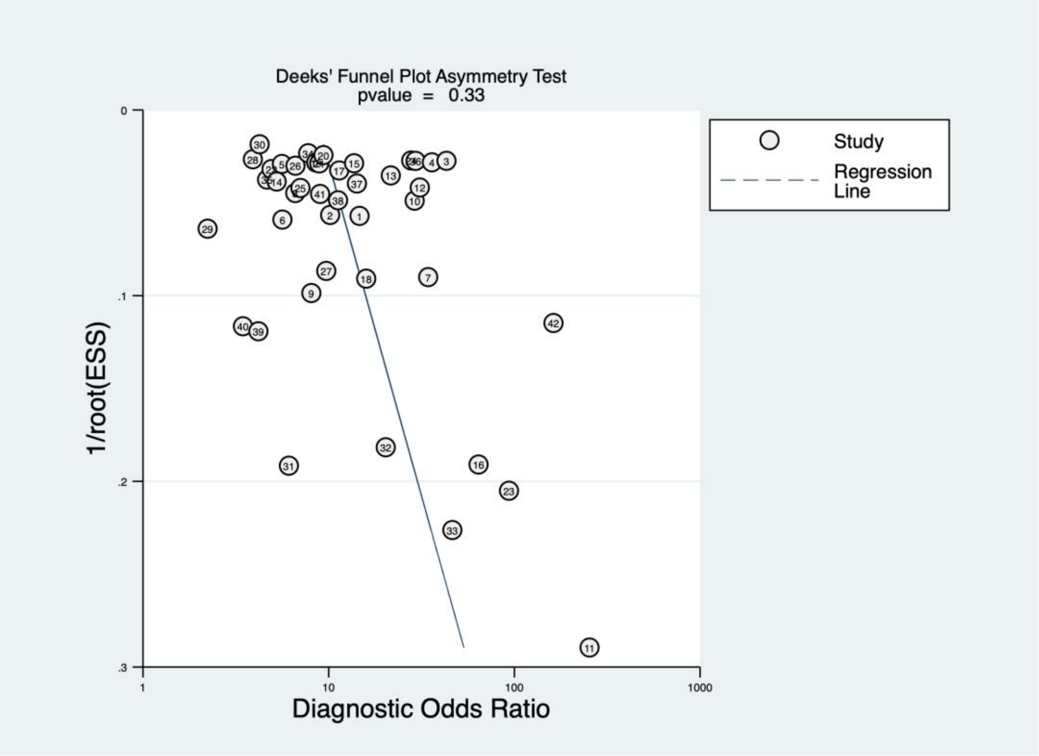

Figure S35. Publication bias – any cough

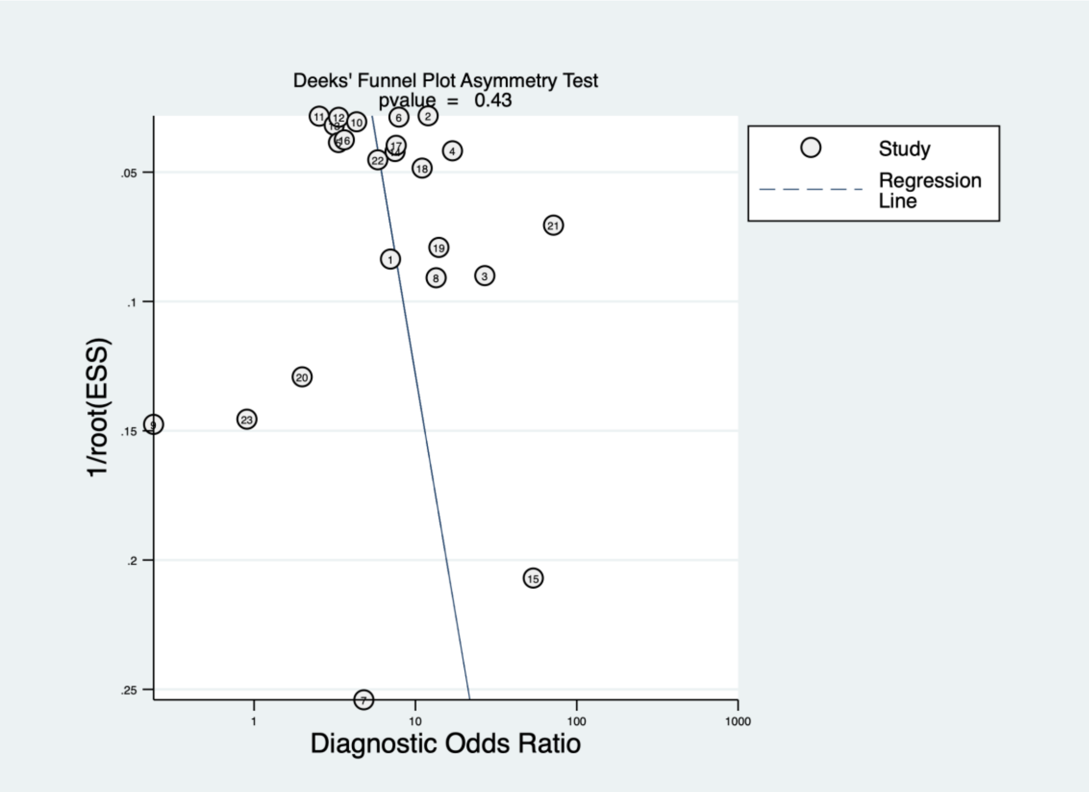

Figure S36. Publication bias – CXR (any abnormality)

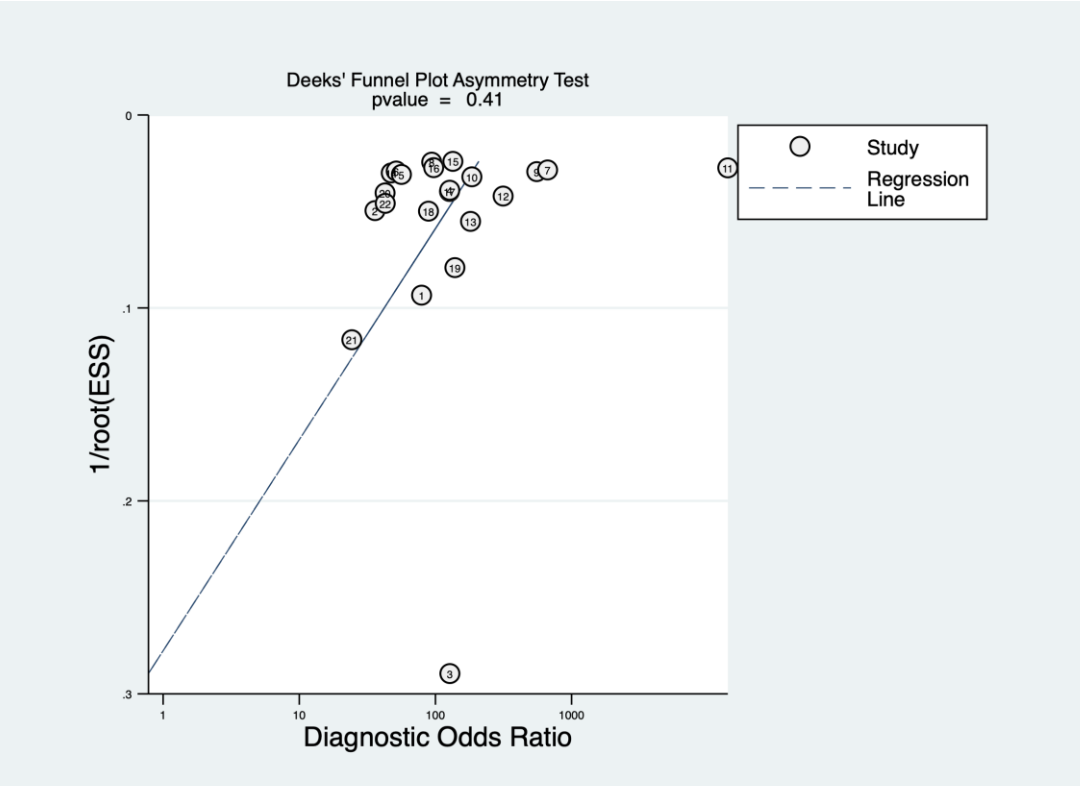

Figure S37. Publication bias – CXR (TB)

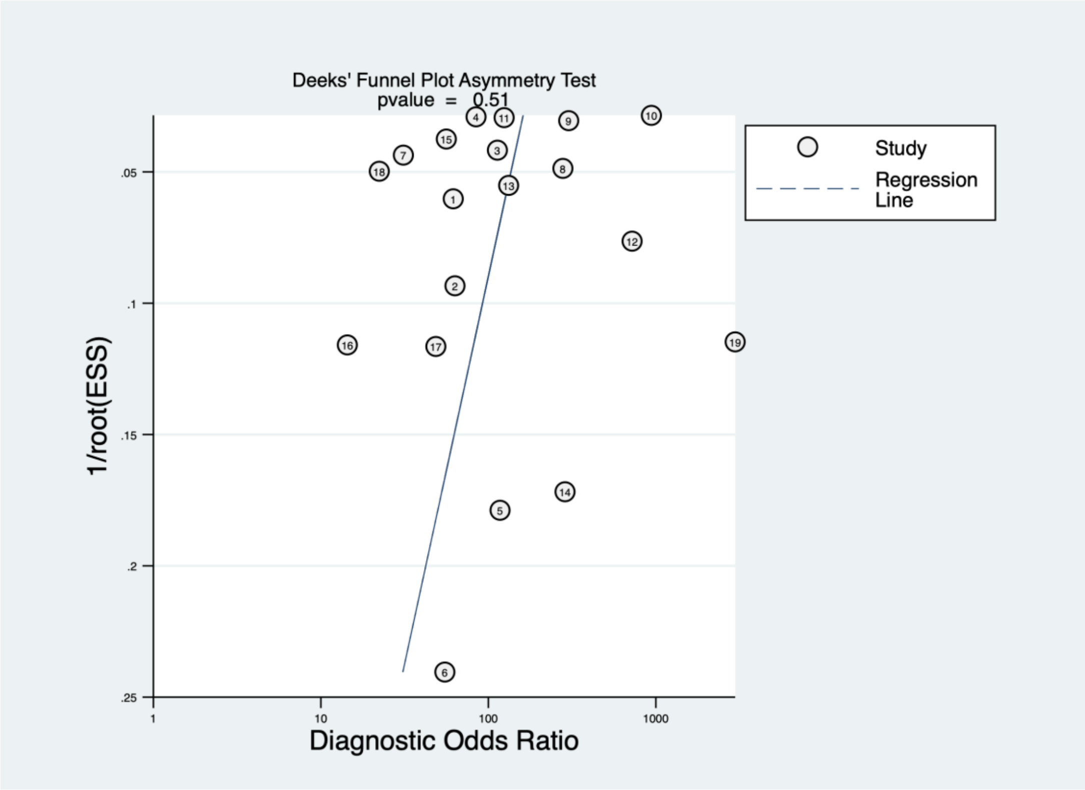

Figure S38. Publication bias – parallel CXR and symptoms

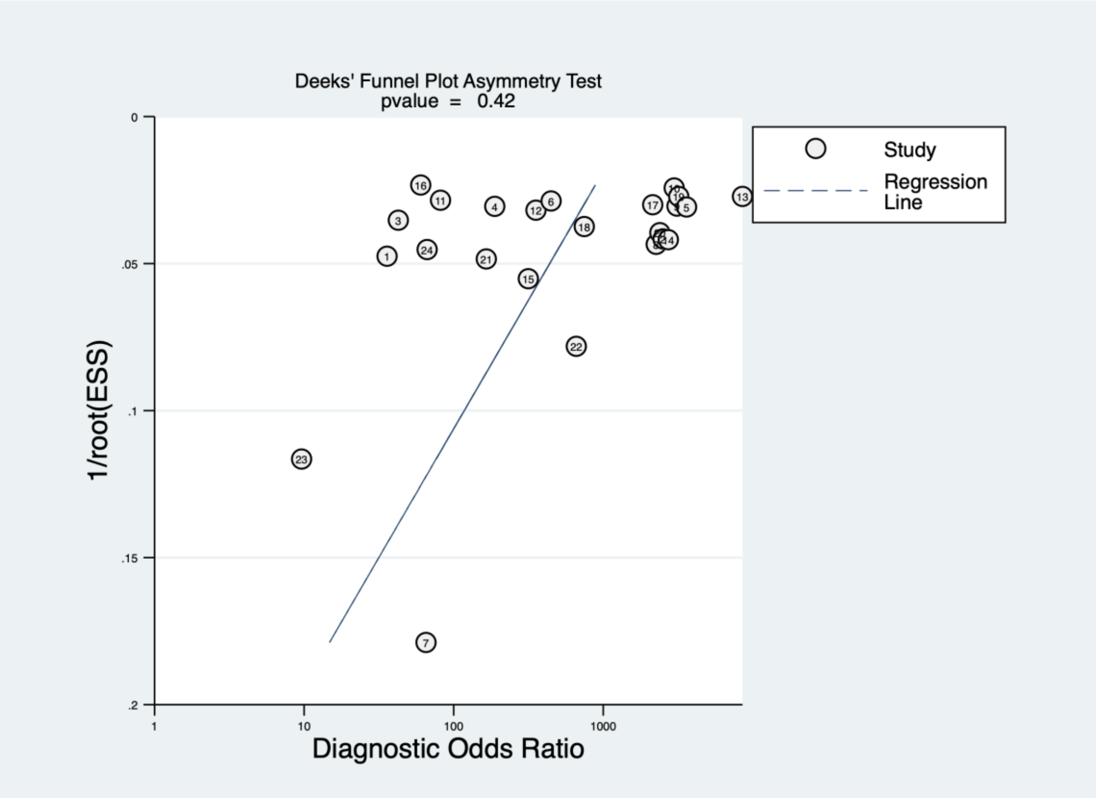

Figure S39. Publication bias – any TB symptoms

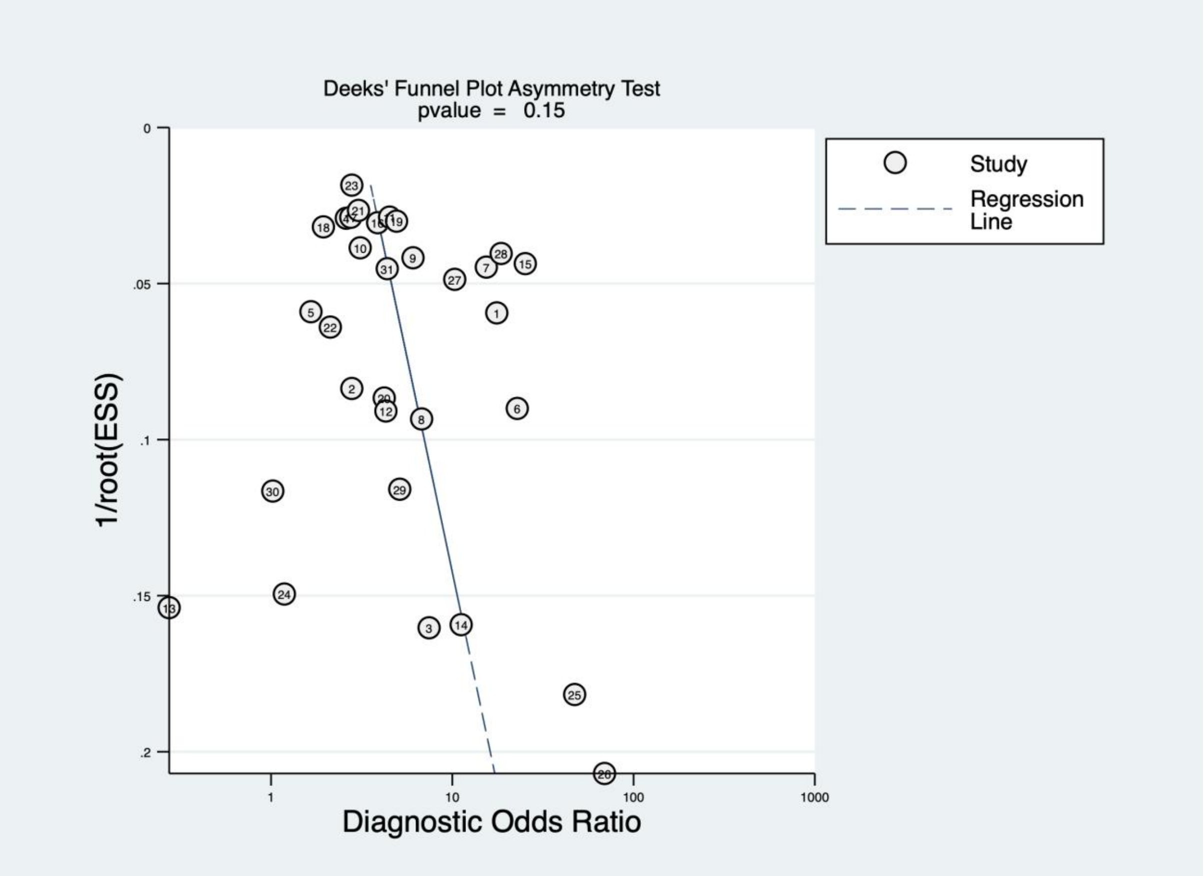

Pooled Test results – stratified analysis

Sensitivity	Studies (n)	Estimate	95% CI lower	95% CI upper	I^2
cough >2 weeks	41	0.41	0.35	0.47	74.11
Any cough	21	0.5	0.42	0.59	79.02
CXR (any abnormality)	24	0.94	0.91	0.96	52.26
CXR (TB abnormality)	18	0.87	0.78	0.92	67.91
Cough and CXR (parallel)	24	1	0.98	1	5.32
Any TB symptom	32	0.71	0.63	0.78	81.33
Xpert	110	0.87	0.85	0.9	72.89
Ultra	6	0.91	0.75	0.97	72.52
LAMP	12	0.91	0.85	0.95	79.5
SAT	4	0.98	0.88	1	52.59
TST	14	0.67	0.52	0.8	93.20
QuantiFERON	31	0.78	0.72	0.83	78.67
T-Spot.TB	34	0.87	0.84	0.91	68.91
**Specificity**					
cough >2 weeks	41	0.94	0.93	0.96	98
Any cough	21	0.87	0.81	0.91	99.28
CXR (any abnormality)	24	0.89	0.86	0.92	99.57
CXR (TB abnormality)	18	0.95	0.92	0.97	92.53
Cough and CXR (parallel)	24	0.84	0.81	0.87	99.38
Any TB symptom	32	0.68	0.57	0.78	99.54
Xpert	110	0.98	0.97	0.98	47.87
Ultra	6	0.95	0.83	0.99	69.9
LAMP	12	0.95	0.91	0.98	73.41
SAT	4	0.9	0.79	0.96	84.47
TST	14	0.69	0.55	0.79	93.77
QuantiFERON	31	0.76	0.67	0.83	84.2
T-Spot.TB	34	0.83	0.78	0.87	78.14

Supplementary table S2: Pooled test property estimates for HIV negative populations in **high incidence settings** (n=376 papers) with number of studies, 95% confidence intervals, and the I^2 statistic as measure of heterogeneity

Sensitivity	Studies (n)	Estimate	95% CI lower	95% CI upper	I^2
cough >2 weeks	insufficient data				
Any cough	insufficient data				
CXR (any abnormality)	insufficient data				
CXR (TB abnormality)	insufficient data				
Cough and CXR (parallel)	insufficient data				
Any TB symptom	insufficient data				
Xpert	42	0.88	0.84	0.91	63.31
Ultra	4	0.95	0.87	0.98	57.6
LAMP	2	0.9	0.86	0.93	
SAT	insufficient data				
TST	14	0.74	0.6	0.84	82.93
QuantiFERON	40	0.86	0.83	0.9	57.28
T-Spot.TB	19	0.89	0.84	0.93	48.99
**Specificity**					
cough >2 weeks	insufficient data				
Any cough	insufficient data				
CXR (any abnormality)	insufficient data				
CXR (TB abnormality)	insufficient data				
Cough and CXR (parallel)	insufficient data				
Any TB symptom	insufficient data				
Xpert	42	0.99	0.98	0.99	32.71
Ultra	4	0.98	0.96	0.99	2.18
LAMP	2	0.93	0.88	0.96	
SAT	insufficient data				
TST	14	0.71	0.53	0.84	93.86
QuantiFERON	40	0.95	0.9	0.97	44.17
T-Spot.TB	19	0.77	0.67	0.85	84.53

Supplementary table S3: Pooled test property estimates for HIV negative populations in **low incidence settings** (n=206 papers) with number of studies, 95% confidence intervals, and the I^2 statistic as measure of heterogeneity

Sensitivity	Studies (n)	Estimate	95% CI lower	95% CI upper	I^2
cough >2 weeks	46	0.41	0.35	0.46	72.47
Any cough	24	0.52	0.44	0.6	76.12
CXR (any abnormality)	24	0.94	0.91	0.96	52.26
CXR (TB abnormality)	20	0.86	0.78	0.91	69.29
Cough and CXR (parallel)	24	1	0.98	1	5.32
Any TB symptom	34	0.71	0.63	0.77	81.52
Xpert	175	0.87	0.85	0.89	70.85
Ultra	11	0.91	0.85	0.95	62.85
LAMP	16	0.9	0.83	0.94	82.93
SAT	4	0.98	0.88	1	52.59
TST	35	0.67	0.58	0.75	87.65
QuantiFERON	70	0.83	0.79	0.86	67.73
T-Spot.TB	47	0.88	0.84	0.9	66.42

Specificity					
cough >2 weeks	46	0.95	0.93	0.96	97.97
Any cough	24	0.87	0.82	0.91	98.95
CXR (any abnormality)	24	0.89	0.86	0.92	99.57
CXR (TB abnormality)	20	0.95	0.93	0.97	92.81
Cough and CXR (parallel)	24	0.84	0.81	0.87	99.38
Any TB symptom	34	0.7	0.6	0.79	99.55
Xpert	175	0.98	0.97	0.98	50.62
Ultra	11	0.95	0.89	0.98	75.4
LAMP	16	0.96	0.93	0.98	71
SAT	4	0.9	0.79	0.96	84.47
TST	35	0.72	0.64	0.79	91.05
QuantiFERON	70	0.9	0.85	0.94	68.73
T-Spot.TB	47	0.83	0.78	0.87	81.82

Supplementary table S4: Pooled test property estimates for **all populations (HIV negative and HIV positive)** with number of studies, 95% confidence intervals, and the I^2 statistic as measure of heterogeneity. TST remains unchanged as no added studies.

Figure S40. Summary receiver operating characteristic for test combinations.

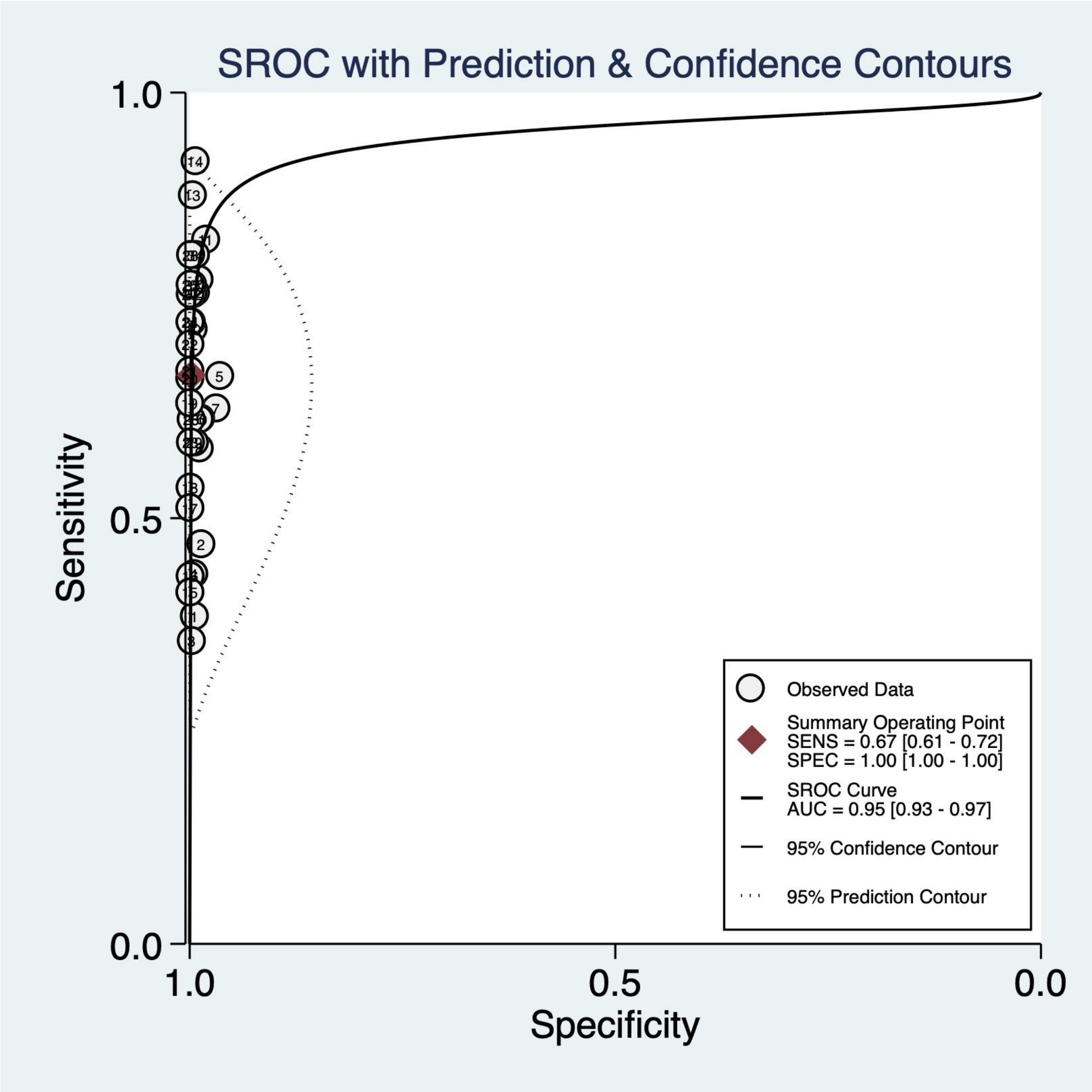

Combined Test properties

test combinations	TP	TN	FP	FN	PPV	NPV	sensitivity	specificity
Cough (2 wk) & CXR (any)	385	98456	545	615	0.414	0.994	0.385	0.995
Cough (any) & CXR (any)	470	97693	1307	530	0.265	0.995	0.470	0.987
Cough (2 wk) & CXR (TB)	357	98802	198	643	0.643	0.994	0.357	0.998
Cough (any) & CXR (TB)	435	98525	475	565	0.478	0.994	0.435	0.995
Any symptoms & CXR (any)	667	95515	3485	333	0.161	0.997	0.667	0.965
Any symptoms & CXR (TB)	618	97733	1267	382	0.328	0.996	0.618	0.987
TST & CXR (any changes)	590	95951	3049	370	0.171	0.996	0.630	0.969
TST & CXR (TB)	583	97891	1109	417	0.345	0.996	0.583	0.989
QFT & CXR (any)	780	97911	1089	220	0.417	0.998	0.780	0.989
QFT & CXR (TB)	722	98604	396	278	0.646	0.997	0.722	0.996
T-Spot.TB & CXR (any)	827	97149	1851	173	0.309	0.998	0.827	0.981
T-Spot.TB & CXR (TB)	766	98327	673	234	0.532	0.998	0.766	0.993
parallel (cough & CXR) & Xpert	880	98683	317	120	0.735	0.999	0.880	0.997
parallel (cough & CXR) & Ultra	920	98366	634	80	0.592	0.999	0.920	0.994
Cough (any) & CXR (any) & Xpert	414	98974	26	586	0.941	0.994	0.414	1.000
Cough (any) & CXR (any) & Ultra	432	98948	52	568	0.892	0.994	0.432	0.999
TST & CXR (TB) & Xpert	513	98978	22	487	0.959	0.995	0.513	1.000
TST & CXR (TB) & Ultra	536	98956	44	464	0.924	0.995	0.536	1.000
QFT & CXR (TB) & Xpert	635	98992	8	365	0.988	0.996	0.635	1.000
QFT & CXR (TB) & Ultra	664	98984	16	336	0.977	0.997	0.664	1.000
T-Spot.TB & CXR (TB) & Xpert	674	98987	13	326	0.980	0.997	0.674	1.000
T-Spot.TB & CXR (TB) & Ultra	704	98973	27	296	0.963	0.997	0.704	1.000
TST & parallel (cough & CXR) & Xpert	590	98911	89	410	0.869	0.996	0.590	0.999
QFT & parallel (cough & CXR) & Xpert	730	98968	32	270	0.958	0.997	0.730	1.000
T-Spot.TB & parallel (cough & CXR) & Xpert	774	98946	54	226	0.935	0.998	0.774	0.999
TST & parallel (cough & CXR) & Ultra	616	98823	177	384	0.777	0.996	0.616	0.998
QFT & parallel (cough & CXR) & Ultra	764	98937	63	236	0.923	0.998	0.764	0.999
T-Spot.TB & parallel (cough & CXR) & Ultra	810	98892	108	190	0.883	0.998	0.810	0.999
TST & Xpert	590	98446	554	410	0.515	0.996	0.590	0.994
TST & Ultra	616	97891	1109	384	0.357	0.996	0.616	0.989
QFT & Xpert	730	98802	198	270	0.787	0.997	0.730	0.998
QFT & Ultra	764	98604	396	236	0.659	0.998	0.764	0.996
T-Spot.TB & Xpert	774	98663	337	226	0.697	0.998	0.774	0.997
T-Spot.TB & Ultra	810	98327	673	190	0.546	0.998	0.810	0.993

Supplementary table S5: Estimated pooled test properties for two-test and three-test combinations. TP: true positives, TN: true negatives, FP: false positives, FN: False negatives, PPV: positive predictive value, NPV: negative predictive value. Assumed prevalence scenario 1,000 per 100,000. All test algorithms are sequential, unless indicated by adding “parallel”.

**testcombinations**	**categories**	**dOR**	**lower CI**	**upper CI**
Cough (2 wk) & CXR (any)	symptom-CXR 2 test combi	113.5	97.4	132.2
Cough (any) & CXR (any)	symptom-CXR 2 test combi	66.3	57.9	76.0
Cough (2 wk) & CXR (TB)	symptom-CXR 2 test combi	276.3	228.4	334.2
Cough (any) & CXR (TB)	symptom-CXR 2 test combi	159.7	136.9	186.3
Any symptoms & CXR (any)	symptom-CXR 2 test combi	55.1	48.1	63.1
Any symptoms & CXR (TB)	symptom-CXR 2 test combi	124.6	108.4	143.2
TST & CXR (any changes)	TBI 2 test combinations	53.5	46.8	61.1
TST & CXR (TB)	TBI 2 test combinations	123.3	107.3	141.7
QFT & CXR (any)	TBI 2 test combinations	320.2	272.5	376.3
QFT & CXR (TB)	TBI 2 test combinations	649.0	547.5	769.4
T-Spot.TB & CXR (any)	TBI 2 test combinations	252.4	212.8	299.3
T-Spot.TB & CXR (TB)	TBI 2 test combinations	477.6	405.0	563.3
parallel (cough & CXR) & Xpert	symptom-CXR 3 test combi	2290.1	1837.1	2854.8
parallel (cough & CXR) & Ultra	symptom-CXR 3 test combi	1787.1	1403.7	2275.1
Cough (any) & CXR (any) & Xpert	symptom-CXR 3 test combi	2682.9	1790.2	4020.5
Cough (any) & CXR (any) & Ultra	symptom-CXR 3 test combi	1449.8	1074.8	1955.6
TST & CXR (TB) & Xpert	TBI 3 test combinations	4729.9	3058.6	7314.4
TST & CXR (TB) & Ultra	TBI 3 test combinations	2603.6	1889.4	3587.7
QFT & CXR (TB) & Xpert	TBI 3 test combinations	24670.3	11630.8	52328.7
QFT & CXR (TB) & Ultra	TBI 3 test combinations	13079.7	7753.9	22063.5
T-Spot.TB & CXR (TB) & Xpert	TBI 3 test combinations	15719.1	8983.5	27504.9
T-Spot.TB & CXR (TB) & Ultra	TBI 3 test combinations	9084.4	6042.4	13657.8
TST & parallel (cough & CXR) & Xpert	TBI parallel 3 test combi	1614.7	1265.0	2061.1
QFT & parallel (cough & CXR) & Xpert	TBI parallel 3 test combi	8663.7	5931.8	12653.8
T-Spot.TB & parallel (cough & CXR) & Xpert	TBI parallel 3 test combi	6422.2	4722.1	8734.3
TST & parallel (cough & CXR) & Ultra	TBI parallel 3 test combi	898.0	738.9	1091.3
QFT & parallel (cough & CXR) & Ultra	TBI parallel 3 test combi	5077.2	3810.8	6764.5
T-Spot.TB & parallel (cough & CXR) & Ultra	TBI parallel 3 test combi	3935.3	3074.6	5036.8
TST & Xpert	TBI 2 test combinations	255.3	219.5	297.0
TST & Ultra	TBI 2 test combinations	142.1	123.5	163.6
QFT & Xpert	TBI 2 test combinations	1354.2	1111.5	1649.8
QFT & Ultra	TBI 2 test combinations	805.0	675.0	960.2
T-Spot.TB & Xpert	TBI 2 test combinations	1010.1	841.2	1213.0
T-Spot.TB & Ultra	TBI 2 test combinations	622.1	522.1	741.2

Supplementary table S6: Estimated diagnostic odds ratios (dOR) with 95% confidence intervals for two-test and three-test combinations. Assumed prevalence scenario 1,000 per 100,000. All test algorithms are sequential, unless indicated by adding “parallel”.

PPVs and NNS for different prevalence scenarios

test combinations	2.0%	1.0%	0.5%	0.2%	0.1%
QFT & CXR (TB) & Xpert	0.994	0.988	0.976	0.941	0.888
T-Spot.TB & CXR (TB) & Xpert	0.990	0.980	0.961	0.908	0.832
QFT & CXR (TB) & Ultra	0.988	0.977	0.954	0.893	0.806
T-Spot.TB & CXR (TB) & Ultra	0.981	0.963	0.929	0.838	0.722
TST & CXR (TB) & Xpert	0.979	0.959	0.920	0.821	0.696
QFT & parallel (cough & CXR) & Xpert	0.979	0.958	0.920	0.821	0.696
Cough (any) & CXR (any) & Xpert	0.970	0.941	0.887	0.758	0.611
T-Spot.TB & parallel (cough & CXR) & Xpert	0.967	0.935	0.877	0.740	0.588
TST & CXR (TB) & Ultra	0.961	0.924	0.857	0.706	0.545
QFT & parallel (cough & CXR) & Ultra	0.961	0.923	0.857	0.705	0.544
Cough (any) & CXR (any) & Ultra	0.944	0.892	0.805	0.621	0.450
T-Spot.TB & parallel (cough & CXR) & Ultra	0.938	0.883	0.789	0.599	0.427
TST & parallel (cough & CXR) & Xpert	0.931	0.869	0.768	0.569	0.397
QFT & Xpert	0.882	0.787	0.647	0.423	0.268
TST & parallel (cough & CXR) & Ultra	0.875	0.777	0.633	0.408	0.256
parallel (cough & CXR) & Xpert	0.849	0.735	0.580	0.355	0.216
T-Spot.TB & Xpert	0.823	0.697	0.534	0.313	0.186
QFT & Ultra	0.796	0.659	0.490	0.277	0.160
QFT & CXR (TB)	0.787	0.646	0.476	0.266	0.153
Cough (2 wk) & CXR (TB)	0.784	0.643	0.473	0.263	0.151
parallel (cough & CXR) & Ultra	0.746	0.592	0.419	0.224	0.126
T-Spot.TB & Ultra	0.708	0.546	0.374	0.193	0.106
T-Spot.TB & CXR (TB)	0.697	0.532	0.361	0.184	0.101
TST & Xpert	0.682	0.515	0.346	0.174	0.095
Cough (any) & CXR (TB)	0.649	0.478	0.313	0.154	0.083
QFT & CXR (any)	0.591	0.417	0.263	0.124	0.066
Cough (2 wk) & CXR (any)	0.588	0.414	0.260	0.123	0.066
TST & Ultra	0.529	0.357	0.217	0.099	0.052
TST & CXR (TB)	0.515	0.345	0.207	0.094	0.050
Any symptoms & CXR (TB)	0.496	0.328	0.195	0.088	0.046
T-Spot.TB & CXR (any)	0.474	0.309	0.182	0.081	0.042
Cough (any) & CXR (any)	0.421	0.265	0.152	0.067	0.034
TST & CXR (any changes)	0.294	0.171	0.093	0.039	0.020
Any symptoms & CXR (any)	0.279	0.161	0.087	0.037	0.019

Supplementary table S7: Positive predictive values (PPV) for different prevalence scenarios

test combinations	2.0%	1.0%	0.5%	0.2%	0.1%
parallel (cough & CXR) & Ultra	54	109	217	543	1087
parallel (cough & CXR) & Xpert	57	114	227	568	1136
T-Spot.TB & CXR (any)	60	121	242	604	1209
T-Spot.TB & parallel (cough & CXR) & Ultra	62	124	247	618	1235
T-Spot.TB & Ultra	62	124	247	618	1235
QFT & CXR (any)	64	128	256	641	1282
T-Spot.TB & parallel (cough & CXR) & Xpert	65	129	258	646	1291
T-Spot.TB & Xpert	65	129	258	646	1291
T-Spot.TB & CXR (TB)	65	131	261	653	1306
QFT & parallel (cough & CXR) & Ultra	65	131	262	655	1310
QFT & Ultra	65	131	262	655	1310
QFT & parallel (cough & CXR) & Xpert	68	137	274	685	1369
QFT & Xpert	68	137	274	685	1369
QFT & CXR (TB)	69	138	277	692	1385
T-Spot.TB & CXR (TB) & Ultra	71	142	284	710	1420
T-Spot.TB & CXR (TB) & Xpert	74	148	297	742	1484
Any symptoms & CXR (any)	75	150	300	749	1498
QFT & CXR (TB) & Ultra	75	151	301	753	1505
QFT & CXR (TB) & Xpert	79	157	315	787	1574
Any symptoms & CXR (TB)	81	162	324	809	1619
TST & parallel (cough & CXR) & Ultra	81	162	324	811	1622
TST & Ultra	81	162	324	811	1622
TST & CXR (any changes)	85	170	339	848	1696
TST & parallel (cough & CXR) & Xpert	85	170	339	848	1696
TST & Xpert	85	170	339	848	1696
TST & CXR (TB)	86	172	343	858	1716
TST & CXR (TB) & Ultra	93	186	373	932	1865
TST & CXR (TB) & Xpert	97	195	390	975	1950
Cough (any) & CXR (any)	106	213	426	1064	2128
Cough (any) & CXR (TB)	115	230	460	1149	2299
Cough (any) & CXR (any) & Ultra	116	231	463	1156	2313
Cough (any) & CXR (any) & Xpert	121	242	484	1209	2418
Cough (2 wk) & CXR (any)	130	259	519	1297	2595
Cough (2 wk) & CXR (TB)	140	280	561	1402	2803

Supplementary table S8: Numbers needed to screen (NNS) for different prevalence scenarios (top row)

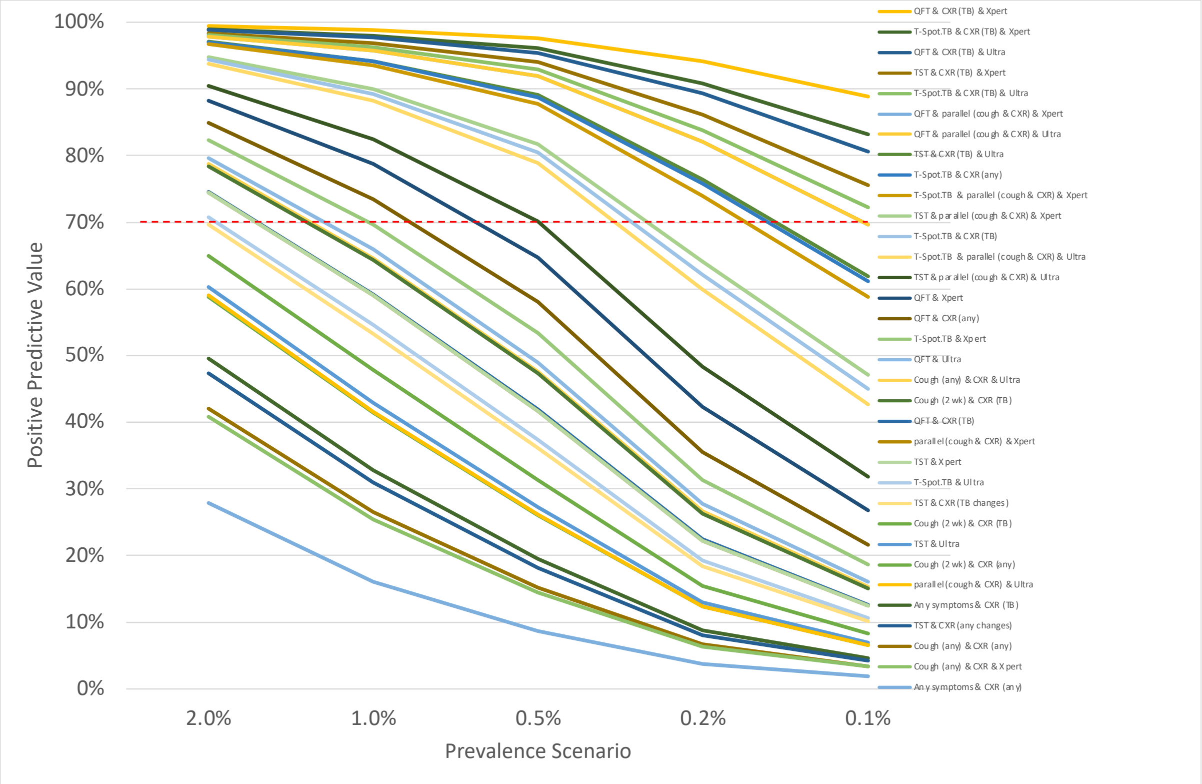

Figure S41. Positive Predictive Values (PPVs) for different prevalence scenarios; chart and legend ordered by PPV at prevalence 0.001 (100 per 100,000). CXR: Chest X-Ray, Xpert: Gene Xpert®, Ultra: Gene Xpert® Ultra, TST: Tuberculin Skin Test, QFT: QuantiFERON. The dashed red line indicates a PPV of 70%

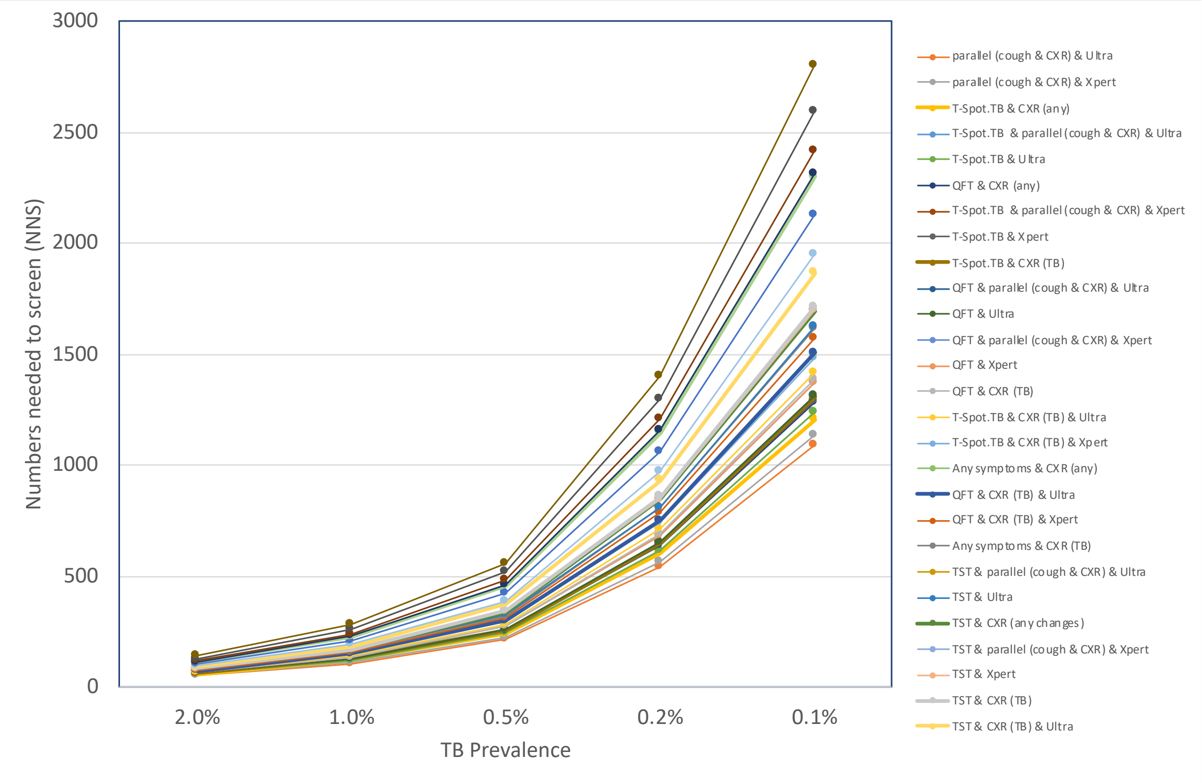

Figure S42. Numbers needed to screen (NNS) for different prevalence scenarios; chart and legend ordered by PPV at prevalence 0.001 (100 per 100,000). CXR: Chest X-Ray, Xpert: Gene Xpert®, Ultra: Gene Xpert® Ultra, TST: Tuberculin Skin Test, QFT: QuantiFERON.

Probabilistic Sensitivity analysis

test combinations	TP	TN	FP	FN	dOR
Cough (2 wk) & CXR (any)	336	98384	470	562	125.3
Cough (any) & CXR (any)	414	97569	1199	480	70.3
Cough (2 wk) & CXR (TB)	305	98735	148	589	345.0
Cough (any) & CXR (TB)	386	98452	415	506	180.9
Any symptoms & CXR (any)	621	95349	3296	278	64.6
Any symptoms & CXR (TB)	559	97624	1160	335	140.4
TST & CXR (any changes)	579	95763	2863	328	59.1
TST & CXR (TB)	528	97781	991	372	139.9
QFT & CXR (any)	739	97778	1000	181	399.2
QFT & CXR (TB)	670	98542	334	232	851.0
T-Spot.TB & CXR (any)	786	97013	1703	134	333.2
T-Spot.TB & CXR (TB)	725	98247	596	195	613.0
parallel (cough & CXR) & Xpert	837	98624	270	91	3372.3
parallel (cough & CXR) & Ultra	888	98280	550	56	2848.0
Cough (any) & CXR (any) & Xpert	388	98964	5	415	17708.5
Cough (any) & CXR (any) & Ultra	411	98945	5	415	18758.7
TST & CXR (TB) & Xpert	468	98959	10	442	10399.2
TST & CXR (TB) & Ultra	492	98930	26	420	4391.8
QFT & CXR (TB) & Xpert	585	98972	4	280	50955.5
QFT & CXR (TB) & Ultra	614	98968	6	284	35128.9
T-Spot.TB & CXR (TB) & Xpert	627	98972	4	280	54664.1
T-Spot.TB & CXR (TB) & Ultra	662	98949	14	252	19201.0
TST & parallel (cough & CXR) & Xpert	538	98877	58	362	2536.1
QFT & parallel (cough & CXR) & Xpert	684	98944	17	229	16995.0
T-Spot.TB & parallel (cough & CXR) & Xpert	727	98919	33	187	11568.5
TST & parallel (cough & CXR) & Ultra	567	98779	138	331	1226.0
QFT & parallel (cough & CXR) & Ultra	719	98905	43	194	8479.3
T-Spot.TB & parallel (cough & CXR) & Ultra	757	98859	80	152	6141.1
TST & Xpert	530	98376	488	358	298.2
TST & Ultra	573	97790	1010	341	162.7
QFT & Xpert	680	98749	147	224	2038.1
QFT & Ultra	719	98517	336	187	1131.5
T-Spot.TB & Xpert	733	98612	280	188	1375.8
T-Spot.TB & Ultra	767	98252	594	149	848.3

Supplementary table S9: Probabilistic sensitivity analysis – minimum estimates for true positives (TP), true negatives (TN), FP: false positives (FP), false negatives (FN) and diagnostic Odds Ratios (dORs)

test combinations	TP	TN	FP	FN	dOR
Cough (2 wk) & CXR (any)	436	98528	620	671	103.1
Cough (any) & CXR (any)	532	97790	1412	584	63.1
Cough (2 wk) & CXR (TB)	409	98846	251	696	231.3
Cough (any) & CXR (TB)	478	98598	550	614	139.4
Any symptoms & CXR (any)	709	95670	3662	393	47.1
Any symptoms & CXR (TB)	663	97858	1388	439	106.6
TST & CXR (any changes)	688	96106	3216	422	48.7
TST & CXR (TB)	632	98002	1196	468	110.7
QFT & CXR (any)	819	98002	1193	256	263.3
QFT & CXR (TB)	764	98668	462	340	479.9
T-Spot.TB & CXR (any)	863	97274	1986	209	202.4
T-Spot.TB & CXR (TB)	805	98404	756	278	376.7
parallel (cough & CXR) & Xpert	908	98736	372	152	1583.2
parallel (cough & CXR) & Ultra	949	98439	709	111	1187.0
Cough (any) & CXR (any) & Xpert	564	98993	31	612	2898.8
Cough (any) & CXR (any) & Ultra	564	98993	55	589	1736.3
TST & CXR (TB) & Xpert	561	98988	40	550	2538.4
TST & CXR (TB) & Ultra	578	98971	67	525	1636.3
QFT & CXR (TB) & Xpert	717	98995	25	370	7748.6
QFT & CXR (TB) & Ultra	715	98993	43	384	4299.3
T-Spot.TB & CXR (TB) & Xpert	717	98995	25	370	7748.6
T-Spot.TB & CXR (TB) & Ultra	746	98987	45	340	4832.0
TST & parallel (cough & CXR) & Xpert	644	98939	119	460	1161.3
QFT & parallel (cough & CXR) & Xpert	774	98986	56	319	4281.0
T-Spot.TB & parallel (cough & CXR) & Xpert	819	98965	83	270	3637.1
TST & parallel (cough & CXR) & Ultra	659	98860	218	430	694.0
QFT & parallel (cough & CXR) & Ultra	807	98965	97	283	2915.8
T-Spot.TB & parallel (cough & CXR) & Ultra	844	98923	145	229	2523.8
TST & Xpert	643	98519	647	455	215.2
TST & Ultra	659	97984	1213	441	120.6
QFT & Xpert	772	98847	249	312	982.0
QFT & Ultra	818	98665	470	293	585.8
T-Spot.TB & Xpert	823	98717	398	262	780.5
T-Spot.TB & Ultra	850	98406	754	235	472.5

Supplementary table S10: Probabilistic sensitivity analysis – maximum estimates for true positives (TP), true negatives (TN), FP: false positives (FP), false negatives (FN) and diagnostic Odds Ratios (dORs)

test combinations	TP	TN	FP	FN	dOR
QFT & CXR (TB) & Xpert	635	98979	21	365	8378.511
T-Spot.TB & CXR (TB) & Xpert	674	98974	26	336	7600.743
QFT & CXR (TB) & Ultra	664	98959	41	336	4755.555
T-Spot.TB & CXR (TB) & Ultra	704	98948	52	336	3972.067
TST & CXR (TB) & Xpert	521	98971	29	479	3667.750
Cough (any) & CXR (any) & Xpert	414	98974	26	586	2670.964
QFT & parallel (cough & CXR) & Xpert	730	98918	82	336	2613.160
QFT & parallel (cough & CXR) & Ultra	730	98918	82	336	2613.160
parallel (cough & CXR) & Xpert	880	98683	317	120	2284.333
T-Spot.TB & parallel (cough & CXR) & Xpert	774	98895	105	336	2182.391
TST & CXR (TB) & Ultra	544	98941	59	456	2016.190
parallel (cough & CXR) & Ultra	920	98366	634	80	1785.375
TST & parallel (cough & CXR) & Xpert	598	98883	117	336	1503.888
Cough (any) & CXR (any) & Ultra	432	98948	52	568	1442.049
T-Spot.TB & parallel (cough & CXR) & Ultra	810	98791	209	336	1139.590
TST & parallel (cough & CXR) & Ultra	626	98766	234	336	785.191
QFT & Xpert	730	98485	515	336	416.278
T-Spot.TB & Xpert	774	98347	653	336	347.245
Cough (2 wk) & CXR (TB)	357	98802	198	643	276.688
QFT & CXR (TB)	722	97970	1030	278	247.249
T-Spot.TB & CXR (TB)	766	97693	1307	234	244.174
TST & Xpert	598	98267	733	336	239.125
QFT & Ultra	764	97970	1030	336	216.462
TST & CXR (any changes)	598	98267	733	402	199.867
T-Spot.TB & Ultra	810	97693	1307	336	180.308
Cough (any) & CXR (TB)	435	98525	475	565	159.628
T-Spot.TB & CXR (any)	827	95406	3594	173	127.087
Any symptoms & CXR (TB)	618	97733	1267	382	124.614
TST & Ultra	626	97535	1465	336	124.065
Cough (2 wk) & CXR (any)	385	98456	545	615	113.386
TST & CXR (TB)	592	97535	1465	408	96.428
QFT & CXR (any)	780	94971	4029	220	83.664
Cough (any) & CXR (any)	470	97693	1307	530	66.294
Any symptoms & CXR (any)	667	95515	3485	333	54.999

Supplementary table S11: Sensitivity analysis accounting for potentially very low specificity of TBI tests in high TBI prevalence scenarios (migrants). Specificity for TST, T-Spot.TB and QFT have been artificially lowered by 16% in keeping with TBI positivity findings in migrant TBI screening in England. Specificity values amended for TST 0.79→ 0.63, T-Spot.TB 0.83→ 0.67, QFT 0.9→ 0.74. (TP), true negatives (TN), FP: false positives (FP), false negatives (FN) and diagnostic Odds Ratios (dORs)

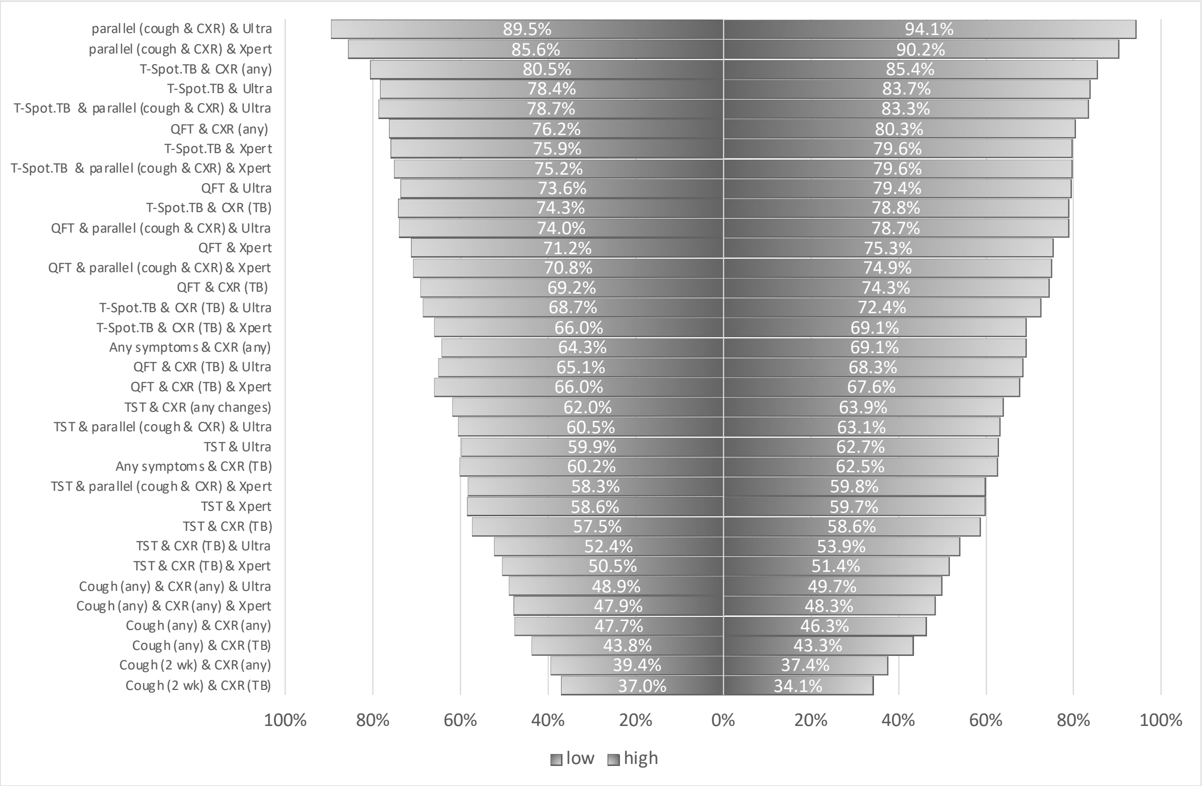

Figure S43. Tornado plot showing the range of values which overall sensitivity could take, assuming worst- and best-case scenarios for test algorithms (percentage ranges on x axis), calculated from the probabilistic distribution and taking minimum and maximum values of TP and FN. CXR: Chest X-Ray, Xpert: Gene Xpert®, Ultra: Gene Xpert® Ultra, TST: Tuberculin Skin Test, QFT: QuantiFERON.

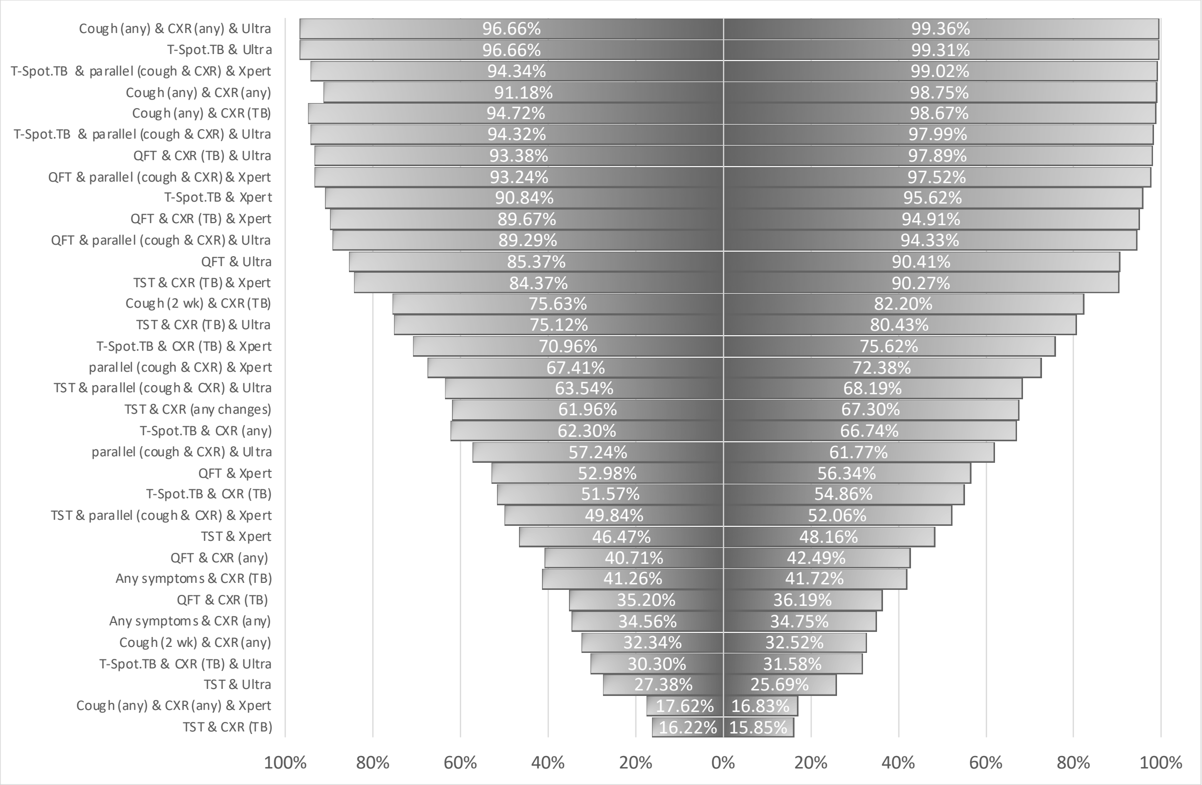

Figure S44. Tornado plot of minimum and maximum probabilistic values of PPV for test algorithms. CXR: Chest X-Ray, Xpert: Gene Xpert®, Ultra: Gene Xpert® Ultra, TST: Tuberculin Skin Test, QFT: QuantiFERON

specificity	low	high
Cough (2 wk) & CXR (any)	99.52%	99.37%
Cough (any) & CXR (any)	98.79%	98.58%
Cough (2 wk) & CXR (TB)	99.85%	99.75%
Cough (any) & CXR (TB)	99.58%	99.44%
Any symptoms & CXR (any)	96.66%	96.31%
Any symptoms & CXR (TB)	98.83%	98.60%
TST & CXR (any changes)	97.10%	96.76%
TST & CXR (TB)	99.00%	98.79%
QFT & CXR (any)	98.99%	98.80%
QFT & CXR (TB)	99.66%	99.53%
T-Spot.TB & CXR (any)	98.27%	98.00%
T-Spot.TB & CXR (TB)	99.40%	99.24%
parallel (cough & CXR) & Xpert	99.73%	99.63%
parallel (cough & CXR) & Ultra	99.44%	99.28%
Cough (any) & CXR (any) & Xpert	99.99%	99.97%
Cough (any) & CXR (any) & Ultra	99.99%	99.94%
TST & CXR (TB) & Xpert	99.99%	99.96%
TST & CXR (TB) & Ultra	99.97%	99.93%
QFT & CXR (TB) & Xpert	100.00%	99.97%
QFT & CXR (TB) & Ultra	99.99%	99.96%
T-Spot.TB & CXR (TB) & Xpert	100.00%	99.97%
T-Spot.TB & CXR (TB) & Ultra	99.99%	99.95%
TST & parallel (cough & CXR) & Xpert	99.94%	99.88%
QFT & parallel (cough & CXR) & Xpert	99.98%	99.94%
T-Spot.TB & parallel (cough & CXR) & Xpert	99.97%	99.92%
TST & parallel (cough & CXR) & Ultra	99.86%	99.78%
QFT & parallel (cough & CXR) & Ultra	99.96%	99.90%
T-Spot.TB & parallel (cough & CXR) & Ultra	99.92%	99.85%
TST & Xpert	99.51%	99.35%
TST & Ultra	98.98%	98.78%
QFT & Xpert	99.85%	99.75%
QFT & Ultra	99.66%	99.53%
T-Spot.TB & Xpert	99.72%	99.60%
T-Spot.TB & Ultra	99.40%	99.24%

Supplementary table S12: Minimum and maximum probabilistic values of specificity for test algorithms. CXR: Chest X-Ray, Xpert: Gene Xpert®, Ultra: Gene Xpert® Ultra, TST: Tuberculin Skin Test, QFT: QuantiFERON.
